# Discovery of Indeno[1,2-*c*]quinoline Derivatives as Potent Dual Antituberculosis and Anti-Inflammatory Agents

**DOI:** 10.3390/molecules22061001

**Published:** 2017-06-16

**Authors:** Chih-Hua Tseng, Chun-Wei Tung, Chen-Hsin Wu, Cherng-Chyi Tzeng, Yen-Hsu Chen, Tsong-Long Hwang, Yeh-Long Chen

**Affiliations:** 1 School of Pharmacy, College of Pharmacy, Kaohsiung Medical University, Kaohsiung 807, Taiwan; chihhua@kmu.edu.tw (C.-H.T.); cwtung@kmu.edu.tw (C.-W.T.); 2 Department of Fragrance and Cosmetic Science, College of Pharmacy, Kaohsiung Medical University, Kaohsiung 807, Taiwan; 3 Research Center for Natural Products and Drug Development, Kaohsiung Medical University, Kaohsiung 807, Taiwan; tzengch@kmu.edu.tw; 4 Center for Infectious Disease and Cancer Research, Kaohsiung Medical University, Kaohsiung 807, Taiwan; 5 Department of Medical Research, Kaohsiung Medical University-Hospital, Kaohsiung 807, Taiwan; 6 Ph.D. Program in Toxicology, Kaohsiung Medical University, Kaohsiung City 807, Taiwan; 7 Department of Medicinal and Applied Chemistry, College of Life Science, Kaohsiung Medical University, Kaohsiung 807, Taiwan; creator770326@hotmail.com; 8 Division of Infectious Diseases, Department of Internal Medicine, Kaohsiung Medical University-Hospital, Kaohsiung Medical University, Kaohsiung 807, Taiwan; 9 School of Medicine, College of Medicine, Kaohsiung Medical University, Kaohsiung 807, Taiwan; 10 Department of Laboratory Medicine, Kaohsiung Medical University-Hospital, Kaohsiung Medical University, Kaohsiung 807, Taiwan; 11 Center for Dengue Fever Control and Research, Kaohsiung Medical University, Kaohsiung 807, Taiwan; 12 Graduate Institute of Natural Products, College of Medicine, and Chinese Herbal Medicine Research Team, Healthy Aging Research Center, Chang Gung University, Taoyuan 333, Taiwan; 13 Research Center for Chinese Herbal Medicine, Research Center for Food and Cosmetic Safety, and Graduate Institute of Health Industry Technology, College of Human Ecology, Chang Gung University of Science and Technology, Taoyuan 333, Taiwan; 14 Department of Anesthesiology, Chang Gung Memorial Hospital, Taoyuan 333, Taiwan

**Keywords:** indeno[1,2-*c*]quinoline, antimycobacterial activity, anti-inflammatory activity

## Abstract

A series of indeno[1,2-*c*]quinoline derivatives were designed, synthesized and evaluated for their anti-tuberculosis (anti-TB) and anti-inflammatory activities. The minimum inhibitory concentration (MIC) of the newly synthesized compound was tested against *Mycobacterium tuberculosis* H_37_R_V_. Among the tested compounds, (*E*)-*N*′-[6-(4-hydroxypiperidin-1-yl)-11*H*-indeno[1,2-*c*]quinolin-11-ylidene]isonicotino-hydrazide (**12**), exhibited significant activities against the growth of *M. tuberculosis* (MIC values of 0.96 μg/mL) with a potency approximately equal to that of isoniazid (INH), an anti-TB drug. Important structure features were analyzed by quantitative structure–activity relationship (QSAR) analysis to give better insights into the structure determinants for predicting the anti-TB activity. The anti-inflammatory activity was induced by superoxide anion generation and neutrophil elastase (NE) release using the formyl-l-methionyl-l-leucyl-l-phenylalanine (fMLF)-activated human neutrophils method. Results indicated that compound **12** demonstrated a potent dual inhibitory effect on NE release and superoxide anion generation with IC_50_ values of 1.76 and 1.72 μM, respectively. Our results indicated that compound **12** is a potential lead compound for the discovery of dual anti-TB and anti-inflammatory drug candidates. In addition, 6-[3-(hydroxymethyl)piperidin-1-yl]-9-methoxy-11*H*-indeno[1,2-*c*]quinolin-11-one (**4g**) showed a potent dual inhibitory effect on NE release and superoxide anion generation with IC_50_ values of 0.46 and 0.68 μM, respectively, and is a potential lead compound for the discovery of anti-inflammatory drug candidates.

## 1. Introduction

Tuberculosis (TB) is an infectious disease caused by *Mycobacterium tuberculosis* which primarily affects the lungs, causing an intense local inflammatory response that is critical to the pathogenesis of tuberculosis [1,2]. Therefore, TB is a chronic inflammatory condition in which regulatory and pro-inflammatory processes occur that contribute to severe lung pathology, leading to lung tissue necrosis and the promotion of mycobacterial dissemination and transmission [3]. Anti-inflammatory drugs, especially corticosteroids, are currently used for adjunctive therapy in most severe life-threatening forms of tuberculosis, such as meningitis and pericarditis [4]. The importance of developing new drugs with dual anti-inflammatory and antimycobacterial activities is amplified by the emergence of multi-drug resistant (MDR) strains and the global human immunodeficiency virus (HIV) pandemic [5,6,7]. The anti-leprosy drug clofazimine [8] exhibited dual anti-inflammatory and antimycobacterial activities. However, its potency is not sufficient, motivating the design and search for novel agents.

Quinoline-based anti-TB compound bedaquiline is a highly potent anti-TB agent, has a novel mode of action and was recently approved for the treatment of multidrug-resistant tuberculosis (MDR-TB) [9]. Currently there is considerable interest in the synthesis of indeno[2,1-c]quinoline derivatives, because of their antimycobacterial activities [10,11,12]. However, the isomeric indeno[1,2-*c*]quinoline derivatives have attracted only limited attention. Over the past few years, we have been particularly interested in the synthesis of fluoroquinolone and benzofuroquinoline derivatives for anti-TB evaluations [13,14,15,16,17]. We have also synthesized certain polycyclic heterocycles such as 9-anilinoacridine, 9-phenoxyacridine, 4-phenoxyfuro[2,3-*b*]quinoline, quinolin-2(1*H*)-one, furo[3,2:3,4]naphtha[1,2-*d*]imidazole and benzo[f]indole-4,9-dione derivatives for the evaluation of their anti-inflammatory activities [18,19,20,21,22,23,24,25,26,27,28]. In continuation of our efforts to identify potential anti-TB agents with novel types of structures which are distinct from those of existing drugs, we herein describe the synthesis of certain indeno[1,2-*c*]quinoline derivatives for anti-inflammatory and anti-TB evaluations. The aim of our current study is to identify novel skeletons that exhibit dual anti-inflammatory and antimycobacterial activities with low cytotoxicities.

## 2. Results and Discussion

### 2.1. Chemistry

**Scheme 1:**
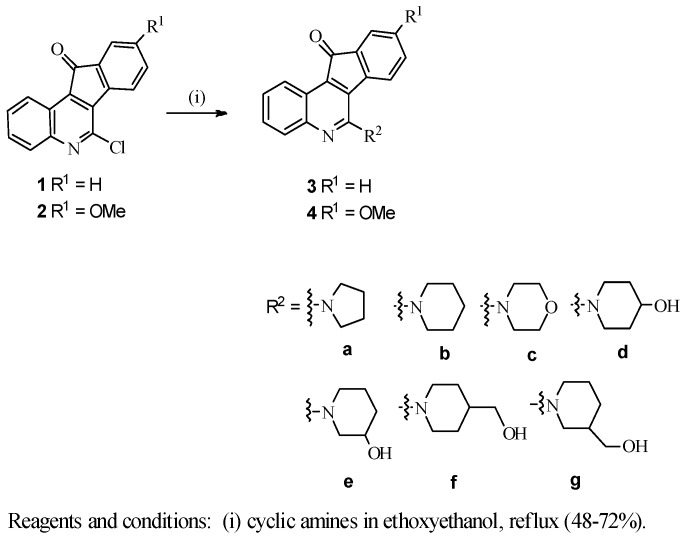
Synthesis of indeno[1,2-*c*]quinoline derivatives **3a**–**4g**.

According to our previously reported procedures [29], 2-hydroxy-3-phenylquinoline-4-carboxylic acid was treated with POCl_3_ to afford 6-chloro-11*H*-indeno[1,2-*c*]quinolin-11-one (**1**), which was then reacted with cyclic amines to give 6-cycloamino-11*H*-indeno[1,2-*c*]quinolin-11-ones (**3a**–**3g**). Accordingly, compounds **4a**–**4g** were prepared from the known 6-chloro-9-methoxy-11H-indeno [1,2-*c*]quinolin-11-one (**2**) [29] under similar reaction conditions as outlined in Scheme 1.

**Scheme 2:**
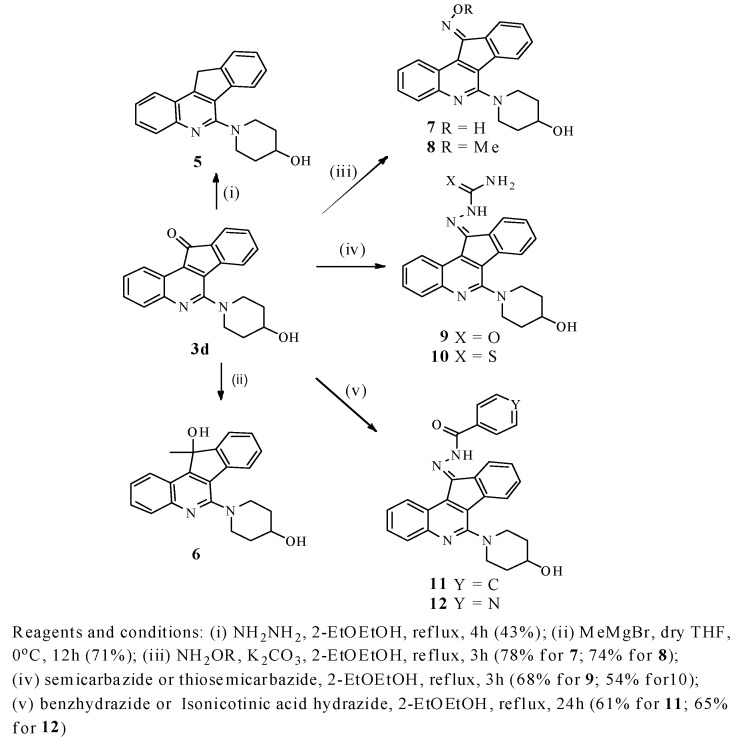
Synthesis of indeno[1,2-*c*]quinoline derivatives **5**–**12**.

Among these compounds, 6-(4-hydroxypiperidin-1-yl)-11*H*-indeno[1,2-*c*]quinolin-11-one (**3d**) exhibited the most potent activity against the growth of *Mycobacterium tuberculosis* (minimum inhibitory concentration (MIC) value of 2.09 μg/mL), and therefore, we decided to select **3d** as a lead compound for structural optimization as described in Scheme 2. The Wolff–Kishner reduction of **3d** with hydrazine afforded 1-(11*H*-indeno[1,2-*c*]quinolin-6-yl)piperidin-4-ol (**5**) in 43% yield. Treatment of **3d** with methyl magnesium bromide (Grignard reagent) afforded 6-(4-hydroxypiperidin-1-yl)-11-methyl-11*H*-indeno[1,2-*c*]quinolin-11-ol (**6**) in 71% yield. (*E*)-6-(4-hydroxypiperidin-1-yl)-11*H*-indeno[1,2-*c*]quinolin-11-one oxime (**7**) and (*E*)-6-(4-hydroxypiperidin-1-yl)-11*H*-indeno[1,2-*c*]quinolin-11-one *O*-methyl oxime (**8**) were obtained from **3d** by the treatment with NH_2_OH and NH_2_OMe, respectively. (*E*)-2-[6-(4-hydroxypiperidin-1-yl)-11*H*-indeno[1,2-*c*]quinolin-11-ylidene]hydrazinecarboxamide (**9**) and (*E*)-2-(6-(4-hydroxypiperidin-1-yl)-11*H*-indeno[1,2-*c*]quinolin-11-ylidene)hydrazinecarbothioamide (**10**) were obtained from **3d** by the treatment with semicarbazide and thiosemicarbazide, respectively. (*E*)-*N*′-[6-(4-hydroxypiperidin-1-yl)-11*H*-indeno[1,2-*c*]quinolin-11-ylidene]benzohydrazide (**11**) and (*E*)-*N*′-(6-(4-hydroxypiperidin-1-yl)-11H-indeno[1,2-c]quinolin-11-ylidene)isonicotinohydrazide (**12**) were obtained from **3d** by the treatment with benhydrazide and isonicotinic acid hydrazide, respectively.

### 2.2. Results and Discussion

The anti-TB activities of indeno[1,2-*c*]quinoline derivatives are summarized in Table 1. The 6-Cycloamino-11*H*-indeno[1,2-*c*]quinolin-11-ones (**3a**–**3g**) were found to be significantly inhibit the growth of *M. tuberculosis*, with MICs ranging from 2.09 to 4.97 μg/mL with >87% survival rate of Vero cells at a concentration of 20 μg/mL. Among them, 6-(4-hydroxypiperidin-1-yl)-11*H*-indeno[1,2-*c*]quinolin-11-one (**3d**) was the most active, with MIC of 2.09 μg/mL. However, their respective 9-methoxy counterparts **4a**–**4g** were inactive (MIC > 17 μg/mL) with an exception of 9-methoxy-6-(4-hydroxypiperidin-1-yl)-11*H*-indeno[1,2-*c*]quinolin-11-one (**4d**) which exhibited the MIC of 7.74 μg/mL. Further structural derivatization of **3d** resulted in two potential anti-TB agents, (*E*)-*N*′-[6-(4-hydroxypiperidin-1-yl)-11*H*-indeno[1,2-*c*]quinolin-11-ylidene]benzohydrazide (**11**) and (*E*)-*N′*-(6-(4-hydroxypiperidin-1-yl)-11*H*-indeno[1,2-*c*]quinolin-11-ylidene)isonicotinohydrazide (**12**) with MIC of 1.98 and 0.96 μg/mL. The anti-TB activity of **12** is equally active to the positive isoniazid (INH) with less cytotoxicity to the Vero cell. In addition, our results indicated that it is a feasible approach for the discovery of potential anti-TB agents through condensation of INH with an appropriate ketone.

These indeno[1,2-*c*]quinolin-11-one derivatives were also evaluated for their inhibitory effects on superoxide anion generation and neutrophil elastase (NE) release in formyl-l-methionyl-l-leucyl-l-phenylalanine (fMLF)-activated human neutrophils and results are shown in Table 2. Compounds **3a**–**3c** were inactive while **3d**–**3g** exhibited a potent dual inhibitory effect on neutrophil elastase (NE) release and superoxide anion generation with IC_50_ value in a range of 1.78 and 4.50 μM. These results indicated that the hydroxyl group substituted at the C6-piperidine ring is crucial for anti-inflammatory activities. Among them, 6-(4-hydroxypiperidin-1-yl)-11*H*-indeno[1,2-c]quinolin-11-one (**3d**) was the most potent dual inhibitor on NE release and superoxide anion generation with IC_50_ values of 2.20 and 1.78 μM, respectively.

The same structure–activity relationships were observed for C9-methoxy derivatives in which compounds **4a**–**4c** were inactive, while their respective C6-hydroxypiperidine derivative **4d**–**4g** exhibited a potent dual inhibitory effect on NE release and superoxide anion generation. Among them, 9-methoxy-6-(3-piperidinemethano-1-yl)-11*H*-indeno[1,2-*c*]quinolin-11-one (**4g**) was the most potent dual inhibitor of NE release and superoxide anion generation, with IC_50_ values of 0.46 and 0.68 μM, respectively. Compound **5** was only weakly active, while compound **6** was inactive indicated the reduction of the keto group is unfavorable. The keto group of **3d** can be converted into its oxime derivatives, compounds **7** and **8**, and retain comparable anti-inflammatory activity. However, the semicarbazone derivatives, compounds **9** and **10**, selectively inhibit superoxide anion generation with IC_50_ values of 3.64 and 2.41 μM, respectively. The benzohydrazide derivative 11 was capable of inhibiting superoxide anion generation with IC_50_ value of 1.67 μM but was inactive in the inhibition of NE release. *(E)-N*′-(6-(4-hydroxypiperidin-1-yl)-11*H*-indeno[1,2-*c*]quinolin-11-ylidene)isonicotino hydrazide (**12**) was found to be a potent dual inhibitor of NE release and superoxide anion generation, with IC_50_ values of 1.76 and 1.72 μM, respectively, approximately equal potent to the positive LY294002.

To provide a better understanding of the relationship between the structure descriptors and anti-TB activities, a quantitative structure–activity relationship (QSAR) model was constructed to analyze important features and evaluate prediction performance. First, a total of 13 chemicals with determined MIC values as shown in Table 1 were randomly divided into a training dataset for model construction and a test dataset for independent test of the constructed model. The training and test datasets consist of 9 (**12**, **11**, **3g**, **3c**, **3e**, **3a**, **4d**, **4a** and **5**) and 4 (**3d**, **3f**, **3b** and **4e**) chemicals, respectively. Subsequently, each chemical was transformed into a vector of 1648 descriptors representing its structure features. The sequential feature selection algorithms developed by our group [30,31] were applied to simultaneously select a minimum number of relevant descriptors maximizing the *R*^2^ performance and develop multiple regression models for QSAR analysis. Please note that only the three most important features were considered due to the small number of chemicals in the dataset. In the QSAR analysis, pMIC values were utilized in that the MIC values in μg/mL were converted toμM/mL and then converted to −log MIC (pMIC). The model is expected to be useful for chemicals within the pMIC range from 1.24 to 2.67. The final QSAR model with high performance with *R*^2^ = 0.96, *R*^2^_adj_ = 0.94, root-mean-squared error (RMSE) = 0.085, and *Q*^2^ = 0.86 could well explain the variation of pMIC values representing the anti-TB activity. The constructed QSAR model and three important descriptors are shown in Table 3. SCH-6 (simple chain, order 6) epresents the Chi chain descriptor of order 6 [32] that directly correlates with pMIC values with a high correlation coefficient of 0.83 for the 13 chemicals. CrippenLogP is the Crippen’s LogP descriptor [33]. The hmax (representing the maximum hydrogen E-State value in a molecule) belongs to the descriptors of atom type electrotopological state [34,35,36].

While CrippenLogP and hmax show only a mild negative correlation to pMIC values with correlation coefficient values of −0.13 and −0.01, respectively, for the 13 chemicals, the combination of all three descriptors largely enhances the prediction performance of the QSAR model with a 15% improvement on *R*^2^. The observed and predicted pMIC values on the training dataset are shown in Table 4. To further evaluate the prediction ability of the QSAR model, the model was applied to predict the 4 chemicals in the test dataset. As shown in Table 5, the predicted and observed values for chemicals with exact MIC values are well correlated with a high correlation coefficient of 0.98. Altogether, the good performance of the QSAR models shows the importance of the three descriptors for determining the anti-TB activity. The model could be further tested and improved when more data are available.

## 3. Experimental Section

### 3.1. General

Melting points were determined on a Electrothermal IA9100 melting point apparatus and are uncorrected. Nuclear magnetic resonance (^1^H and ^13^C) spectra were recorded on a Varian Gemini 200 spectrometer or Varian-Unity-400 spectrometer. Chemical shifts were expressed in parts per million (δ) with tetramethylsilane (TMS) as an internal standard and coupling constant (*J*) in hertz (Hz). Thin-layer chromatography was performed on silica gel 60 F-254 plates purchased from E. Merck and Co. IR spectra were measured using a FTIR Perkin-Elmer system-2000 spectrometer (Perkin-Elmer, Waltham, MA, USA). The elemental analyses were performed in the Instrument Center of National Science Council at National Cheng-Kung University using Heraeus CHN-O Rapid EA (Heraeus Co., Harau, Germany), and all values are within ± 0.4% of the theoretical compositions.

### 3.2. General Procedure for the Preparation of 6-Substituted-11H-indeno[1,2-c]quinolin-11-one Compounds ***3a**–**g**, **4a**, **4d**–**g***

A mixture of 6-chloro-11*H*-indeno[1,2-*c*]quinolin-11-one (**1**) or 6-chloro-9-methoxy-11*H*-indeno [1,2-*c*]quinolin-11-one (**2**), cyclic amine (3.0 mmol), and 2-ethoxyethanol (30 mL) was heated at reflux for 3–6 h (TLC monitoring). The solvent was removed in vacuo and the residue was suspended in H_2_O (20 mL). The resulting precipitate that separated was collected, washed with H_2_O, and dried to give a crude solid. The crude product was purified by crystallization from EtOH to afford compounds **3a**–**g, 4a, 4d**–**g**. Preparation of compounds **4b** and **4c** had been described in our previous report [29].

#### 3.2.1. 6-(Pyrrolidin-1-yl)-11*H*-indeno[1,2-*c*]quinolin-11-one (**3a**)

Yield: 78%. m.p.: 163–164 °C. IR (KBr, cm^−1^): 2956, 1710. ^1^H-NMR (400 MHz, CDCl_3_) δ 1.98–2.01 (*m*, 4H, pyrrolidinyl-H), 3.58–3.62 (*m*, 4H, pyrrolidinyl-H), 7.23–7.27 (*m*, 1H, 9-H), 7.35–7.39 (*m*, 1H, 8-H), 7.44–7.50 (*m*, 2H, 3-, 10-H), 7.53–7.57 (*m*, 1H, 2-H), 7.64 (*d*, 1H, *J* = 7.6 Hz, 7-H), 7.78 (*d*, 1H, *J* = 8.8 Hz, 4-H), 8.71 (*dd*, 1H, *J* = 1.6, 8.8 Hz, 1-H). ^13^C-NMR (100 MHz, CDCl_3_) δ 24.93 (2C), 50.16 (2C), 120.10, 123.63, 123.97, 124.24, 125.66, 127.23, 128.29, 129.85, 130.60, 133.22, 134.52, 136.17, 143.95, 149.18, 155.44, 195.74. Anal. calcd. for C_20_H_16_N_2_O: C 79.98, H 5.37, N 9.33; found: C 79.93, H 5.39, N 9.28.

#### 3.2.2. 6-(Piperidin-1-yl)-11*H*-indeno[1,2-*c*]quinolin-11-one (**3b**)

Yield: 75%. m.p.: 165–166 °C. IR (KBr, cm^−1^): 2934, 1715. ^1^H-NMR (400 MHz, CDCl_3_) δ 1.71 (*br s*, 2H, piperidinyl-H), 1.83–1.88 (*m*, 4H, piperidinyl-H), 3.34 (*br s*, 4H, piperidinyl-H), 7.26–7.30 (*m*, 1H, 9-H), 7.42–7.46 (*m*, 1H, 8-H), 7.48–7.52 (*m*, 1H, 3-H), 7.56–7.60 (*m*, 1H, 2-H), 7.64 (*d*, 1H, *J* = 7.2 Hz, 10-H), 7.72 (*d*, 1H, *J* = 7.2 Hz, 7-H), 7.84 (*d*, 1H, *J* = 8.4 Hz, 4-H), 8.74 (*dd*, 1H, *J* = 1.2, 8.0 Hz, 1-H). ^13^C-NMR (100 MHz, CDCl_3_) δ 24.23, 25.87 (2C), 51.10 (2C), 120.67, 123.22, 123.99, 124.19, 126.60, 127.84, 128.70, 129.77, 132.31, 133.07, 134.72, 136.16, 143.59, 149.27, 158.27, 195.64. Anal. calcd. for C_21_H_18_N_2_O**∙**0.1 H_2_O: C 79.77, H 5.80, N 8.86; found: C 79.77, H 5.74, N 8.75.

#### 3.2.3. 6-(Morpholino-1-yl)-11*H*-indeno[1,2-*c*]quinolin-11-one (**3c**)

Yield: 62%. m.p.: 171–173 °C. IR (KBr, cm^−1^): 2838, 1719. ^1^H-NMR (400 MHz, CDCl_3_) δ 3.41–3.44 (*m*, 4H, morpholinyl-H), 3.98–4.01 (*m*, 4H, morpholinyl-H), 7.28–7.32 (*m*, 1H, 9-H), 7.45–7.52 (*m*, 2H, 3-, 8-H), 7.58–7.63 (*m*, 1H, 2-H), 7.65–7.68 (*m*, 2H, 7-, 10- H), 7.86 (*ddd*, 1H, *J* = 0.8, 1.2, 8.4 Hz, 4-H), 8.75 (*ddd*, 1H, *J* = 0.8, 1.6, 8.4 Hz, 1-H). ^13^C-NMR (100 MHz, CDCl_3_) δ 50.24 (2C), 66.79 (2C), 120.89, 123.18, 124.03, 124.48, 127.04, 127.98, 128.92, 130.02, 131.67, 133.13, 134.80, 136.44, 143.06, 149.16, 157.04, 195.24. Anal. calcd. for C_20_H_16_N_2_O_2_: C 75.93, H 5.10, N, 8.86; found: C 76.33, H 5.15, N, 8.82.

#### 3.2.4. 6-(4-Hydroxypiperidin-1-yl)-11*H*-indeno[1,2-*c*]quinolin-11-one (**3d**)

Yield: 68%. m.p.: 208-208 °C. IR (KBr, cm^−1^): 3542, 2919, 1710. ^1^H-NMR (400 MHz, CDCl_3_) δ 1.83–1.92 (*m*, 2H, piperidinyl-H), 2.13–2.17 (*m*, 2H, piperidinyl-H), 3.16–3.18 (*m*, 2H, piperidinyl-H), 3.70–3.75 (*m*, 2H, piperidinyl-H), 3.93–4.02 (*m*, 1H, piperidinyl-H), 7.27–7.31 (*m*, 1H, 9-H), 7.43–7.52 (*m*, 1H, 3-, 8-H), 7.57–7.61 (*m*, 1H, 2-H), 7.64–7.70 (*m*, 2H, 7-, 10- H), 7.86 (*d*, 1H, *J* = 8.4 Hz, 4-H), 8.73 (*dd*, 1H, *J* = 0.8, 8.4 Hz, 1-H). ^13^C-NMR (100 MHz, CDCl_3_) δ 34.37 (2C), 47.81 (2C), 67.90, 120.76, 123.06, 124.01, 124.36, 126.85, 127.76, 128.87, 129.96, 132.08, 133.02, 134.82 ,136.34, 143.32, 149.02, 157.35, 195.41. Anal. calcd. for C_21_H_18_N_2_O_2_: C 76.34, H 5.49, N 8.48; found: C 76.09, H 5.56, N 8.36.

#### 3.2.5. 6-(3-Hydroxypiperidin-1-yl)-11*H*-indeno[1,2-*c*]quinolin-11-one (**3e**)

Yield: 62%. m.p.: 211–212 °C. IR (KBr, cm^−1^): 3383, 2932, 1717. ^1^H-NMR (400 MHz, DMSO-*d*_6_) δ 1.37–1.44 (*m*, 1H, piperidinyl-H), 1.71–1.80 (*m*, 1H, piperidinyl-H), 1.88–1.92 (m, 1H, piperidinyl-H), 1.99–2.01 (*m*, 1H, piperidinyl-H), 2.80 (*br s*, 1H, piperidinyl-H), 2.92 (*br s*, 1H, piperidinyl-H), 3.48 (*d*, 1H, *J* = 12.8 Hz, piperidinyl-H), 3.66 (*d*, 1H, *J* = 12.4 Hz, piperidinyl-H), 3.85 (*br s*, 1H, piperidinyl-H), 4.99 (*br s*, 1H, -OH), 7.37–7.41 (*m*, 1H, 9-H), 7.48–7.52 (*m*, 1H, 8-H), 7.61–7.70 (*m*, 4H, 2-, 3-, 7-, 10-H), 7.80 (*d*, 1H, *J* = 8.4 Hz, 4-H), 8.58 (*dd*, 1H, *J* = 0.8, 8.0 Hz, 1-H). ^13^C-NMR (100 MHz, DMSO-*d*_6_) δ 22.65, 32.81, 49.87, 56.95, 65.55, 119.89, 123.24, 123.49, 124.13, 126.77, 127.58, 129.22, 130.12, 131.57, 132.31, 135.47 ,135.54 , 142.55, 148.44, 157.10, 194.83. Anal. calcd. for C_21_H_18_N_2_O_2_**∙**1.2H_2_O: C 71.65, H 5.84, N 7.96; found: C 71.49, H 5.77, N 7.90.

#### 3.2.6. 6-(4-Piperidinemethano-1-yl)-11*H*-indeno[1,2-*c*]quinolin-11-one (**3f**)

Yield: 64%. m.p.: 223–224 °C. IR (KBr, cm^−1^): 3394, 2928, 1712. ^1^H-NMR(400 MHz, CDCl_3_) δ 1.58–1.62 (*m*, 4H, piperidinyl-H), 1.80 (*br s*, 1H, OH), 1.97 (*d*, 2H, *J* = 11.2 Hz, piperidinyl-H), 2.99 (*m*, 2H, piperidinyl-H), 3.66 (*d*, 2H, *J* = 6.4 Hz, 4′-CH_2_OH), 3.83 (*d*, 2H, *J* = 13.2Hz, piperidinyl-H), 7.27–7.31 (*m*, 1H, 9-H), 7.43–7.52 (*m*, 2H, 3-, 8-H), 7.56–7.61 (*m*, 1H, 2-H), 7.64–7.69 (*m*, 2H, 7-, 10-H), 7.85 (*d*, 1H, *J* = 8.4 Hz, 4-H), 8.74 (*dd*, 1H, *J* = 1.6, 8.0 Hz, 1-H). ^13^C-NMR (100 MHz, CDCl_3_) δ 28.72 (2C), 38.45, 50.09 (2C), 67.79, 120.72, 123.15, 124.00, 124.27, 126.71, 127.82, 128.78, 129.85, 132.20, 133.05, 134.76, 136.21, 143.46, 149.20, 157.10, 195.55. Anal. calcd. for C_22_H_20_N_2_O_2_**∙**0.2H_2_O: C 75.93, H 5.91, N 8.05; found: C 76.13, H 5.91, N 7.86.

#### 3.2.7. 6-(3-Piperidinemethano-1-yl)-11*H*-indeno[1,2-*c*]quinolin-11-one (**3g**)

Yield: 54%. m.p.: 220–221 °C. IR (KBr, cm^−1^): 3355, 2936, 1715. ^1^H-NMR (400MHz, CDCl_3_) δ 1.31–1.40 (*m*, 1H, piperidinyl-H), 1.75–1.88 (*m*, 2H, piperidinyl-H), 1.92–1.99 (*m*, 1H, piperidinyl-H), 2.10–2.18 (*m*, 1H, piperidinyl-H), 2.52 (*br s*, 1H, 3′-CH_2_OH), 3.05–3.20 (*m*, 2H, piperidinyl-H), 3.59–3.66 (*m*, 3H, piperidinyl-H), 3.75–3.79 (*m*, 1H, piperidinyl-H), 7.25–7.29 (*m*, 1H, 9-H), 7.40–7.44 (*m*, 1H, 8-H), 7.46–7.51 (*m*, 1H, 3-H), 7.55–7.59 (*m*, 1H, 2-H), 7.62–7.65 (*m*, 2H, 7-, 10-H), 7.81 (*d*, 1H, *J* = 8.4 Hz, 4-H), 8.72 (*dd*, 1H, *J* = 1.2, 8.8 Hz, 1-H). ^13^C-NMR (100 MHz, CDCl_3_) δ 24.43, 26.73, 38.34, 51.58, 51.89, 65.23, 120.51, 123.18, 124.01, 124.28, 126.52, 127.38, 128.73, 129.97, 131.95, 132.97, 134.73, 136.46, 143.42, 149.03, 157.40, 195.46. Anal. calcd. for C_22_H_20_N_2_O_2_: C 76.72, H 5.85, N 8.13; found: C, 76.66, H 5.86, N 8.00.

#### 3.2.8. 9-Methoxy-6-(pyrrolidin-1-yl)-11*H*-indeno[1,2-*c*]quinolin-11-one (**4a**)

Yield: 79%. m.p.: 155–156 °C. IR (KBr, cm^−1^): 2934, 1715. ^1^H-NMR (400 MHz, CDCl_3_) δ 1.99 (*m*, 4H, pyrrolidinyl-H), 3.57 (*m*, 4H, pyrrolidinyl-H), 3.86 (*s*, 3H, 9-OMe), 6.92 (*dd*, 1H, *J* = 2.4, 8.4 Hz, 8-H), 7.20 (*d*, 1H, *J* = 2.4 Hz, 10-H), 7.34–7.39 (*m*, 2H, 2-, 7-H), 7.50–7.54 (*m*, 1H, 3-H), 7.76 (*d*, 1H, *J* = 8.4 Hz, 4-H), 8.66 (*dd*, 1H, *J* = 1.6, 8.4 Hz, 1-H). ^13^C-NMR (100 MHz, CDCl_3_) δ 24.84 (2C), 49.98 (2C), 55.72, 110.71, 118.59, 120.28, 123.67, 124.60, 125.67, 127.23, 129.33, 131.68, 135.19, 135.75, 135.91, 148.56, 155.25, 160.28, 195.55. Anal. calcd. for C_21_H_18_N_2_O_2_: C 76.34, H 5.49, N 8.48; found: C 76.04, H 5.56, N 8.38.

#### 3.2.9. 6-(4-Hydroxypiperidin-1-yl)-9-methoxy-11*H*-indeno[1,2-*c*]quinolin-11-one (**4d**)

Yield: 72%. m.p.: 197–198 °C. IR (KBr, cm^−1^): 3295, 2939, 1713. ^1^H-NMR (400 MHz, CDCl_3_) δ 1.81–1.90 (*m*, 2H, piperidinyl-H), 2.12–2.16 (*m*, 2H, piperidinyl-H), 3.09–3.15 (*m*, 2H, piperidinyl-H), 3.67–3.72 (*m*, 2H, piperidinyl-H), 3.87 (*s*, 3H, 9-OMe), 3.95–3.99 (*m*, 1H, piperidinyl-H), 6.94 (*dd*, 1H, *J* = 2.4, 8.4 Hz, 8-H), 7.21 (*d*, 1H, *J* = 2.4 Hz, 10-H), 7.41–7.45 (*m*, 1H, 2-H), 7.53–7.57 (m, 2H, 3-, 7-H), 7.81 (*d*, 1H, *J* = 8.0 Hz, 4-H), 8.67–8.70 (m, 1H, 1-H). ^13^C-NMR (100 MHz, CDCl_3_) δ 34.41 (2C), 47.62 (2C), 55.77, 67.94, 110.78, 118.84, 120.93, 123.70, 124.08, 126.78, 127.86, 129.38, 132.99, 135.04, 135.33, 135.86, 148.59, 157.14, 160.68, 195.30. Anal. calcd. for C_22_H_20_N_2_O_3_: C 73.32, H 5.59, N 7.77; found: C 73.18, H, 5.80, N 7.52.

#### 3.2.10. 6-(3-Hydroxypiperidin-1-yl)-9-methoxy-11*H*-indeno[1,2-*c*]quinolin-11-one (**4e**)

Yield: 66%. m.p.: 191–192 °C. IR (KBr, cm^−1^): 3408, 2937, 1714. ^1^H-NMR (400 MHz, CDCl_3_) δ 1.58 (*br s*, 1H, piperidinyl-H), 1.83–1.85 (*m*, 3H, piperidinyl-H), 3.36–3.52 (*m*, 3H, piperidinyl-H), 3.83 (*s*, 3H, 9-OMe), 3.94–3.97 (*m*, 1H, piperidinyl-H), 4.10 (*s*, 1H, piperidinyl-H), 6.87–6.92 (*m*, 1H, 10-H), 7.14–7.17 (*m*, 1H, 8-H), 7.37–7.44 (*m*, 2H, 2-, 3-H), 7.51–7.54 (*m*, 1H, 7-H), 7.74–7.77 (*m*, 1H, 4-H), 8.61–8.65 (*m*, 1H, 1-H). ^13^C-NMR (100 MHz, CDCl_3_) δ 20.26, 32.35, 51.75, 54.10, 55.70, 65.46, 110.69, 118.87, 120.85, 123.64, 124.29, 126.83, 127.93, 129.78, 133.39, 134.60, 134.80, 134.83, 147.44, 156.07, 160.67, 194.81. Anal. calcd. for C_22_H_20_N_2_O_3_**∙**1.5H_2_O: C 68.20, H 5.99, N 7.23; found: C 68.23, H 5.77, N 7.11.

#### 3.2.11. 6-[4-(Hydroxymethyl)piperidin-1-yl]-9-methoxy-11*H*-indeno[1,2-*c*]quinolin-11-one (**4f**)

Yield: 68%. m.p.: 218–219 °C. IR (KBr, cm^−1^): 3352, 2928, 1711. ^1^H-NMR (400 MHz, DMSO-*d*_6_) δ 1.40–1.49 (*m*, 2H, piperidinyl-H), 1.62 (*m*, 1H, piperidinyl-H), 1.86 (*d*, 2H, *J* = 11.2 Hz, piperidinyl-H), 2.81–2.87 (*m*, 2H, piperidinyl-H), 3.39–3.44 (*m*, 2H, piperidinyl-H), 3.64 (*d*, 2H, *J* = 11.2 Hz, piperidinyl-H), 3.83 (*s*, 3H, 9-OMe), 4.59 (*t*, 1H, *J* = 5.2 Hz, -OH), 7.10–7.13 (*m*, 2H, 3-, 8-H), 7.44–7.49 (*m*, 2H, 2-, 7-H), 7.57–7.61 (*m*, 1H, 10-H), 7.74 (*d*, 1H, *J* = 8.0 Hz, 4-H), 8.50 (*dd*, 1H, *J* = 0.8, 8.4 Hz, 1-H). ^13^C-NMR (100 MHz, DMSO-*d*_6_) δ 28.49, 38.10, 49.68 (2C), 55.75 (2C), 65.84, 110.79, 118.93, 120.01, 122.92, 124.29, 126.70, 127.58, 129.50, 132.58, 134.31, 134.36, 134.91, 147.83, 157.18, 160.38, 194.52. Anal. calcd. for C_23_H_22_N_2_O_3_**∙**0.25H_2_O: C 72.89, H 6.00, N 7.39; found: C 72.54, H 5.96, N 7.33.

#### 3.2.12. 6-[3-(Hydroxymethyl)piperidin-1-yl]-9-methoxy-11*H*-indeno[1,2-*c*]quinolin-11-one (**4g**)

Yield: 67%. m.p.: 243–244 °C. IR (KBr, cm^−1^): 3401, 2929, 1714. ^1^H-NMR (400 MHz, DMSO-*d*_6_) δ 1.11–1.17 (*m*, 1H, piperidinyl-H), 1.78–1.92 (*m*, 4H, piperidinyl-H), 2.58–2.63 (*m*, 1H, piperidinyl-H), 2.77–2.82 (*m*, 1H, piperidinyl-H), 3.28–3.32 (*m*, 2H, piperidinyl-H), 3.57 (*d*, 1H, *J* = 12.4 Hz, piperidinyl-H), 3.75 (*d*, 1H, *J* = 11.2 Hz, piperidinyl-H), 3.84 (*s*, 3H, 9-OMe), 4.58 (*t*, 1H, *J* = 4.8 Hz, -OH), 7.10–7.14 (*m*, 2H, 3-, 8-H), 7.45–7.53 (*m*, 2H, 2-, 7-H), 7.58–7.62 (*m*, 1H, 10-H), 7.75–7.77 (*m*, 1H, 4-H), 8.51–8.54 (*m*, 1H, 1-H). ^13^C-NMR (100 MHz, DMSO-*d*_6_) δ 24.44, 26.74, 50.61, 53.40, 55.78 (2C), 64.07, 110.79, 119.03, 120.03, 122.95, 124.54, 126.74, 127.60, 129.55, 132.67, 134.35 (2C), 134.97, 147.88, 157.34, 160.40, 194.59. Anal. calcd. for C_23_H_22_N_2_O_3_∙0.25H_2_O: C 72.89, H 6.00, N 7.39; found: C 72.94, H 5.96, N 7.40.

#### 3.2.13. 1-(11*H*-Indeno[1,2-*c*]quinolin-6-yl)piperidin-4-ol (**5**)

A mixture of **3d** (0.25 g, 1.0 mmol), and 80% hydrazine monohydrate (1 mL) in 2-ethoxyethanol (30 mL) was refluxed for 4 h (TLC monitoring). The solvent was removed in vacuo, and the residue was poured into H_2_O (100 mL). The resulting precipitate that separated was collected and crystallized from EtOH to give **5** (0.26 g, 82%). m.p.: 211–212 °C. IR (KBr, cm^−1^): 3384, 2926, 2928, 1573. ^1^H-NMR (400 MHz, CDCl_3_) δ 1.88–1.97 (*m*, 2H, piperidinyl-H), 2.14–2.19 (*m*, 2H, piperidinyl-H), 3.14 (*br s*, 2H, piperidinyl-H), 3.76–3.80 (*m*, 2H, piperidinyl-H), 3.97 (*br s*, 1H, piperidinyl-H), 4.18 (s, 2H, 11-CH_2_), 7.32–7.36 (*m*, 1H, 9-H), 7.40–7.47 (*m*, 2H, 2-, 8-H), 7.57–7.64 (*m*, 2H, 3-, 10-H), 7.89–8.00 (*m*, 3H, 1-, 4-, 7-H). ^13^C-NMR (100 MHz, CDCl_3_) δ 34.63 (2C), 35.96, 47.77 (2C), 68.41, 122.61, 123.58, 123.74, 124.51 (2C), 126.21, 126.96, 128.30, 128.39, 128.80, 140.65, 142.63, 146.07, 151.69, 157.95. Anal. calcd. for C_21_H_20_N_2_O**∙**0.1H_2_O : C 79.27, H 6.40, N 8.80; found: C 79.24, H 6.48, N 8.89.

#### 3.2.14. 6-(4-Hydroxypiperidin-1-yl)-11-methyl-11*H*-indeno[1,2-*c*]quinolin-11-ol (**6**)

A mixture of **3d** (0.25 g, 1.0 mmol), dry THF (20 mL) and 2 M methyl magnesium bromide (2 mL) was stirred at 0 °C for 12 h (TLC monitoring). The solvent was removed in vacuo, and the residue was poured into H_2_O (100 mL). The resulting precipitate that separated was collected and crystallized from EtOH to give **6** (0.25 g, 71%). m.p.: 146–147 °C. IR (KBr, cm^−1^): 3351, 2926, 1372. ^1^H-NMR (400 MHz, CDCl_3_) δ 1.76–1.99 (*m*, 2H, piperidinyl-H), 1.92 (*s*, 3H, 11-CH_3_), 2.10–2.19 (*m*, 2H, piperidinyl-H), 3.04–3.27 (m, 2H, piperidinyl-H), 3.75–3.78 (m, 2H, piperidinyl-H), 3.96 (*m*, 1H, piperidinyl-H), 7.36–7.48 (*m*, 3H, 2-, 8-, 9-H), 7.61–7.67 (*m*, 2H, 3-, 10-H), 7.87(*d*, 1H, *J* = 7.2 Hz, 7-H), 7.98 (*br s*, 1H, 4-H), 8.35–8.37 (*d*, 1H, *J* = 8.0 Hz, 1-H). ^13^C-NMR (100 MHz, CDCl_3_) δ 26.83, 34.51 (2C), 47.58 (2C), 67.98, 81.34, 118.87, 122.48 (2C), 122.85 (2C), 124.34 (2C), 124.67, 127.59, 128.74, 128.99 (2C), 129.26, 150.83. Anal. calcd. for C_22_H_22_N_2_O_2_**∙**0.7H_2_O: C 73.60, H 6.57, N 7.80; found: C 73.90, H 6.78, N 7.43.

#### 3.2.15. (*E*)-6-(4-Hydroxypiperidin-1-yl)-11*H*-indeno[1,2-*c*]quinolin-11-one Oxime (**7**)

A mixture of **3d** (0.27 g, 1.0 mmol), potassium carbonate (0.56 g，4.00 mmol) and NH_2_OH·HCl (0.14 g, 2.0 mmol) in 2-ethoxyethanol (30 mL) was refluxed for 3 h (TLC monitoring). The solvent was removed in vacuo, and the residue was poured into H_2_O (100 mL). The resulting precipitate that separated was collected and crystallized from EtOH to give **11** (0.27 g, 78%). m.p.: 155–156 °C. IR (KBr, cm^−1^): 3329, 2942. ^1^H-NMR (400 MHz, DMSO-*d*_6_) δ 1.68–1.76 (*m*, 2H, piperidinyl-H), 1.98–2.00 (*m*, 2H,piperidinyl-H), 2.99 (*br s*, 2H, piperidinyl-H), 3.55–3.58 (*m*, 2H,piperidinyl-H), 3.74 (*br s*, 1H, piperidinyl-H), 4.83 (*br s*, 1H, -OH), 7.40–7.49 (*m*, 2H, 8-, 9-H), 7.57–7.64 (*m*, 2H, 2-, 3-H), 7.80–7.90 (*m*, 2H, 7-, 10-H), 8.43 (*d*, 1H, *J* = 7.6 Hz, 4-H), 8.79 (*dd*, 1H, *J* = 1.2, 8.4 Hz, 1-H), 13.39 (*s*, 1H, NOH). ^13^C-NMR (100 MHz, DMSO-*d*_6_) δ 34.17 (2C), 48.04 (2C), 67.98, 120.79, 122.37, 125.30, 125.56, 126.19, 128.09, 128.25, 128.41, 128.87, 129.17, 130.87, 138.25, 139.59,146.94, 153.91, 157.40. Anal. calcd. for C_21_H_19_N_3_O_2_**∙**0.2H_2_O: C 72.27, H 5.60, N 12.04; found: C 72.67, H 5.72, N 11.63.

#### 3.2.16. (*E*)-6-(4-Hydroxypiperidin-1-yl)-11*H*-indeno[1,2-*c*]quinolin-11-one *O*-methyl Oxime (**8**)

Compound **8** was prepared from **3d** and NH_2_OMe∙HCl by the same procedure as described for the preparation of **7**. Yield: 74%. m.p.: 183–184 °C. IR (KBr, cm^−1^): 3350, 2938, 1025. ^1^H-NMR (400 MHz, DMSO-*d*_6_) δ 1.66–1.74 (*m*, 2H, piperidinyl-H), 1.96–1.99 (*m*, 2H, piperidinyl-H), 2.96 (*br s*, 2H, piperidinyl-H), 3.51–3.54 (*m*, 2H, piperidinyl-H), 3.72 (*br s*, 1H, piperidinyl-H), 4.32 (*s*, 3H, =NOCH_3_), 4.83 (*br s*, 1H, -OH), 7.35–7.48 (*m*, 2H, 8-, 9-H), 7.54–7.63 (*m*, 2H, 2-, 3-H), 7.77–7.81 (*m*, 2H, 7-, 10-H), 8.22 (*d*, 1H, *J* = 7.6Hz, 4-H), 8.72 (*dd*, 1H, *J* = 0.8, 8.4 Hz, 1-H). ^13^C-NMR (100 MHz, DMSO-*d*_6_) δ 34.19 (2C), 48.01 (2C), 64.39, 66.62, 120.60, 122.57, 125.21, 125.79, 126.62, 128.09, 128.34, 128.68, 129.33, 130.00, 131.54, 138.73,138.77, 147.05, 153.78, 157.28. Anal. calcd. for C_22_H_21_N_3_O_2_: C 73.52, H 5.89, N 11.69; found: C 73.24, H 5.93, N 11.49.

#### 3.2.17. *(E)-*2-[6-(4-Hydroxypiperidin-1-yl)-11*H*-indeno[1,2-*c*]quinolin-11-ylidene]hydrazine Carboxamide (**9**)

A mixture of **3d** (0.27 g, 1.0 mmol) and semicarbazide (0.15 g, 2.0 mmol) in 2-ethoxyethanol (30 mL) was refluxed for 3 h (TLC monitoring). The solvent was removed in vacuo, and the residue was poured into H_2_O (100 mL). The resulting precipitate that separated was collected and crystallized from EtOH to give **9** (0.27 g, 68%). m.p.: 289–290 °C. IR (KBr, cm^−1^): 3468, 1725. ^1^H-NMR (400 MHz, DMSO-*d*_6_) δ 1.69–1.78 (*m*, 2H, piperidinyl-H), 1.99–2.02 (*m*, 2H, piperidinyl-H), 3.03 (*br s*, 2H, piperidinyl-H), 3.59–3.61 (*m*, 2H, piperidinyl-H), 3.76 (*br s*, 1H, OH), 7.00 (*br s*, 2H, NH_2_), 7.45–7.51 (*m*, 2H, 8-, 9-H), 7.58–7.65 (*m*, 2H, 2-, 3-H), 7.82 (*d*, 1H, *J* = 8.8 Hz, 10-H), 7.94 (*d*, 1H, *J* = 7.6 Hz, 7-H), 8.30 (*d*, 1H, *J* = 7.6 Hz, 4-H), 9.15 (*d*, 1H, *J* =8.4 Hz, 1-H), 10.45 (*br s*, 1H, NH). ^13^C-NMR (100 MHz, DMSO-*d*_6_) δ 34.04 (2C), 47.79 (2C), 65.71, 120.65, 122.49, 124.98, 125.75, 125.87, 126.86, 127.80, 128.18, 129.44, 130.39, 138.37, 141.52 ,143.90, 145.40, 155.74, 156.57, 181.34. Anal. calcd. for C_22_H_21_N_5_O_2_**∙**0.5H_2_O: C 66.65, H 5.59, N 17.67; found: C 66.69, H, 5.70, N 17.31.

#### 3.2.18. *(E)*-2-[6-(4-Hydroxypiperidin-1-yl)-11*H*-indeno[1,2-*c*]quinolin-11-ylidene]hydrazine Carbothioamide (**10**)

Compound **10** was prepared from **3d** and thiosemicarbazide by the same procedure as described for the preparation of **9**. Yield: 54%. m.p.: 304–305 °C. IR (KBr, cm^−1^): 3408, 1601. ^1^H-NMR (400 MHz, DMSO-*d*_6_) δ 1.68–1.77 (*m*, 2H, piperidinyl-H), 1.99–2.01 (*m*, 2H, piperidinyl-H), 3.03 (*br s*, 2H, piperidinyl-H), 3.57–3.60 (*m*, 3H, OH, piperidinyl-H), 3.75 (*br s*, 1H, piperidinyl-H), 7.46–7.50 (*m*, 3H, 8-, 9-H), 7.59–7.65 (*m*, 2H, 2-, 3-H), 7.81 (*d*, 1H, *J* = 8.0 Hz, 10-H), 7.91 (*d*, 1H, *J* = 7.2 Hz, 7-H), 8.10 (*d*, 1H, *J* = 7.6 Hz, 4-H), 8.25 (*br* *s*, 1H, NH), 8.99 (*br* *s*, 1H, NH), 9.07 (*d*, 1H, *J* = 7.6 Hz, 1-H), 11.30 (*br s*, 1H, NH). ^13^C-NMR (100 MHz, DMSO-*d*_6_) δ 34.45 (2C), 48.09 (2C), 65.56, 121.45, 121.91, 125.09, 125.50, 127.17, 127.64, 128.62, 130.25, 140.06, 156.12, 156.59, 157.61, 173.59. Anal. calcd. for C_22_H_21_N_5_OS**∙**0.6H_2_O: C 63.78, H 5.40, N 16.90; found: C 63.90, H 5.35, N 16.50.

#### 3.2.19. *(E)-N*′-[6-(4-Hydroxypiperidin-1-yl)-11*H*-indeno[1,2-*c*]quinolin-11-ylidene]benzohydrazide (**11**)

A mixture of **3d** (0.27 g, 1.0 mmol) and benzohydrazide (0.27 g, 2.0 mmol) in 2-ethoxyethanol (30 mL) was refluxed for 24 h (TLC monitoring). The solvent was removed in vacuo, and the residue was poured into H_2_O (100 mL). The resulting precipitate that separated was collected and crystallized from EtOH to give **11** (0.28 g, 61%). m.p.: 258–259 °C. IR (KBr, cm^−1^): 3422, 2937, 1660. ^1^H-NMR (400 MHz, DMSO-*d*_6_) δ 1.70–1.78 (*m*, 2H, piperidinyl-H), 1.99–2.02 (*m*, 2H, piperidinyl-H), 3.03 (*br s*, 2H, piperidinyl-H), 3.58–3.61 (*m*, 2H, piperidinyl-H), 3.76 (*br s*, 1H, piperidinyl-H), 4.83 (*br s*, 1H, -OH), 7.45–7.52 (*m*, 2H, NH, 9-H), 7.61–7.72 (*m*, 5H, Ar-H), 7.81 (*d*, 1H, *J* = 8.4 Hz, 10-H), 7.93 (*d*, 1H, *J* = 7.6 Hz, 7-H), 8.07 (*m*, 2H, Ar-H), 8.26 (*d*, 1H, *J* = 8.0 Hz, 4-H), 9.17 (*br s*, 1H, 1-H), 12.09 (*s*, 1H, NH). ^13^C-NMR (100 MHz, DMSO-*d*_6_) δ 34.20 (2C), 48.00 (2C), 65.71, 120.88, 122.53, 125.39, 125.76, 126.52, 126.94, 127.45, 127.98, 128.11, 128.27 (2C), 128.55, 128.69 (2C), 129.34, 131.48, 132.25, 133.05, 139.60, 140.64, 147.26, 157.17. Anal. calcd. for C_28_H_24_N_4_O_2_**∙**0.6H_2_O: C 73.22, H 5.53, N 12.20; found: C 73.09, H 5.43, N 12.21.

#### 3.2.20. *(E)-N*′-[6-(4-Hydroxypiperidin-1-yl)-11*H*-indeno[1,2-*c*]quinolin-11-ylidene]isonicotino Hydrazide (**12**)

Compound **12** was prepared from **3d** and isonicotinic acid hydride by the same procedure as described for the preparation of **11**. Yield: 65%. m.p.: 282–283 °C. IR (KBr, cm^−1^): 3365, 2948, 1649. ^1^H-NMR (400 MHz, DMSO-*d*_6_) δ 1.68–1.77 (*m*, 2H, piperidinyl-H), 1.99–2.01 (*m*, 2H, piperidinyl-H), 3.02 (*br s*, 2H, piperidinyl-H), 3.57–3.59 (*m*, 2H, piperidinyl-H), 3.75 (*br s*, 1H, piperidinyl-H), 4.84 (*br s*, 1H, -OH), 7.47–7.51 (*m*, 2H, 8-, 9-H), 7.61–7.65 (*m*, 2H, 2-, 3-H), 7.80 (*d*, 1H, *J* = 8.4 Hz, 10-H), 7.90–7.95 (*m*, 3H, Ar-H), 8.27 (*d*, 1H, *J* = 7.2 Hz, 4-H), 8.86 (*d*, 2H, *J* = 5.6 Hz, pyridinyl-H), 9.21 (*br s*, 1H, 1-H), 12.30 (s, 1H, NH). ^13^C-NMR (100 MHz, DMSO-*d*_6_) δ 34.17 (2C), 48.05 (2C), 65.54, 120.79, 122.00 (2C), 122.57, 125.18, 125.80, 126.75, 127.74, 128.00, 128.15, 128.41, 129.39, 131.77, 139.76, 140.47, 147.29, 150.39 (2C), 157.14. Anal. calcd. for C_27_H_23_N_5_O_2_**∙**1.8H_2_O: C 67.29, H 5.56, N 14.53; found: C 67.14, H 5.57, N 14.40.

### 3.3. Anti-Mycobacterium Activity

Primary screening is conducted against *M. tuberculosis* H_37_Rv (ATCC 27294) in BACTEC 12B medium (BD Microbiology Systems, Franklin Lakes, NJ, USA) using a broth microdilution assay to determine the actual minimum inhibitory concentration (MIC) using the Microplate Alamar Blue Assay (MABA) [37]. The MIC is defined as the lowest concentration effecting a reduction in fluorescence of 90% relative to controls. Vero cell (normal cell) viability is assessed on the basis of cellular conversion of 3-(4,5-dimethylthiazol-2-yl)-2,5-diphenyltetrazolium bromide (MTT), into a formazan product using the Promega Cell Titer 96 Non-radioactive Cell Proliferation Assay (Promega, Madison, WI, USA).

### 3.4. Superoxide Generation and Elastase Release

Superoxide generation and elastase release were carried out according to the procedures described previously [38]. Neutrophils (6 × 10^5^/mL) were equilibrated at 37 °C for 2 min and incubated with compounds for 5 min. Neutrophils were then activated by fMLF (100 nM) in the pretreatment of cytochalasin B (1 µg/mL for superoxide generation and 0.5 µg/mL for elastase release) for 10 min. Superoxide anion production was assayed by monitoring the superoxide dismutase-inhibitable reduction of ferricytochrome C. Elastase release was performed using MeO-Suc-Ala-Ala-Pro-Valp-nitroanilide as the elastase substrate.

### 3.5. Quantitative Structure–Activity Relationship (QSAR)

Structure features of 1648 1D and 2D descriptors and PubChem fingerprints for chemicals were firstly generated by using the PaDEL-Descriptor [39] software (http://padel.nus.edu.sg/software/padeldescriptor). The calculations are mainly based on the Chemistry Development Kit [40] with some additional descriptors and fingerprints including atom-type electrotopological state descriptors, McGowan volume, molecular linear free energy relation descriptors, ring counts, count of chemical substructures, binary fingerprints and count of chemical substructures. It has been extensively utilized in many QSAR studies such as non-genotoxic hepatotoxicity prediction [41] and inhibitor design [42]. Sequential feature selection algorithms developed by our group were subsequently applied to simultaneously identify important features and build multiple [30,31] regression models. For each run, the descriptor with highest *R^2^* performance was iteratively appended into the multiple regression model. To avoid overfitting problems, only descriptors with a maximum correlation coefficient to selected descriptors ranging from −0.5 to 0.5 were considered. Due to the small number of chemicals, only the best three descriptors were considered. The descriptor set with the highest *R*^2^ performance is utilized to construct the final multiple regression model for QSAR analysis. The measurements of *R*^2^, *R*_adj_^2^, root-mean-squared error (RMSE), and leave-one-out cross-validated *Q*^2^ were applied to evaluate the performance of the QSAR model.

## 4. Conclusions

We have synthesized certain indeno[1,2-*c*]quinoline derivatives for anti-TB and anti-inflammatory evaluations. Among them, (*E*)-*N*′-[6-(4-hydroxypiperidin-1-yl)-11*H*-indeno[1,2-*c*]quinolin-11-ylidene]-isonicotinohydrazide (**12**), exhibited significant activities against the growth of *M. tuberculosis* (MIC values of 0.96 μg/mL) with a potency approximately equal to that of INH. Compound **12** has also demonstrated a potent dual inhibitory effect on NE release and superoxide anion generation with IC_50_ values of 1.76 and 1.72 μM, respectively. These results indicated that compound **12** is a potential lead compound for the discovery of dual anti-TB and anti-inflammatory drug candidates. In addition, 6-[3-(hydroxymethyl)piperidin-1-yl]-9-methoxy-11*H*-indeno[1,2-*c*]quinolin-11-one (**4g**) showed a potent dual inhibitory effect on NE release and superoxide anion generation with IC_50_ value of 0.46 and 0.68 μM respectively, and is a potential lead compound for the discovery of anti-inflammatory drug candidates. Further studies on the structural optimization and the molecular pharmacological mechanism of compounds **12** and **4g** are ongoing.

## Figures and Tables

**Table 1:** Anti-*Mycobacterium tuberculosis* H_37_Rv activities of indeno[1,2-*c*]quinoline derivatives. Results expressed as minimum inhibitory concentration (MIC) compared to isoniazid (INH).

Compounds	*R*^1^	*R*^2^	MIC (μg/ mL)	Survival Rate of Vero Cells @ 20 μg/mL (%)
**3a**	H	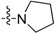	4.56	88.61
**3b**	H	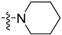	4.93	96.18
**3c**	H	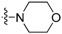	3.12	91.19
**3d**	H	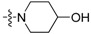	2.09	91.23
**3e**	H	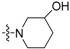	4.97	87.78
**3f**	H	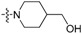	3.98	91.11
**3g**	H	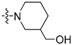	3.07	92.25
**4a**	OMe	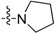	17	89.62
**4b**	OMe	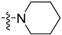	>20	99.55
**4c**	OMe	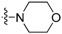	>20	94.47
**4d**	OMe	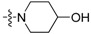	7.74	84.72
**4e**	OMe	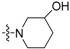	19.10	87.59
**4f**	OMe	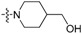	>20	90.26
**4g**	OMe	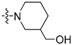	>20	87.60
**5**			18.0	85.41
**6**			>20	89.83
**7**			>20	84.14
**8**			>20	91.48
**9**			>20	81.20
**10**			>20	80.28
**11**			1.98	89.55
**12**			0.96	94.25
**INH**			0.8–1.2	84.80

**Table 2:** Anti-inflammatory activities of indeno[1,2-*c*]quinoline derivatives in formyl-Lmethionyl-l-leucyl-l-phenylalanine (fMLF)-activated human neutrophils (IC_50_ in μM) ^a^.

Compounds	Superoxide Anion	Elastase Release
**3a**	>10	>10
**3b**	>10	>10
**3c**	>10	>10
**3d**	1.78 ± 0.44	2.20 ± 0.69
**3e**	2.90 ± 0.11	2.80 ± 0.09
**3f**	3.37 ± 0.17	4.50 ± 1.32
**3g**	2.77 ± 0.47	2.12 ± 1.19
**4a**	>10	>10
**4b**	>10	>10
**4c**	>10	>10
**4d**	2.82 ± 0.30	2.05 ± 0.24
**4e**	1.89 ± 0.65	2.93 ± 0.70
**4f**	1.93 ± 0.92	0.88 ± 0.30
**4g**	0.68 ± 0.14	0.46 ± 0.08
**5**	4.02 ± 1.08	5.29 ± 0.48
**6**	>10	>10
**7**	0.83 ± 0.47	1.39 ± 0.64
**8**	2.68 ± 0.95	2.13 ± 0.92
**9**	3.64 ± 1.43	>10
**10**	2.41 ± 0.36	>10
**11**	1.67 ± 0.87	>10
**12**	1.72 ± 0.23	1.76 ± 0.49
**LY294002 ^b^**	1.36 ± 0.33	2.21 ± 0.45

^a^ Concentration necessary for 50% inhibition (IC_50_); Results are presented as mean ± SEM (*n =* 3). ^b^ LY294002 (a phosphatidylinositol-3-kinase inhibitor) was used as a positive control for superoxide anion generation and elastase release.

**Table 3:** The quantitative structure–activity relationship (QSAR) analysis result for pMIC values of anti-tuberculosis (TB) activity. hmax.

	Coefficient	Estimate Std. Error	*t*-Value	Pr (>|*t*|)	
(Intercept)	0.5035	1.1585	0.435	0.681929	
SCH-6	11.463	1.0533	10.883	0.000114	***
CrippenLogP	−0.5711	0.1296	−4.407	0.006977	**
hmax	−3.8978	1.4043	−2.776	0.039106	*

*** *p* < 0.001, ** *p* < 0.01, * *p* < 0.05

**Table 4:** The comparison of observed and predicted values for the training dataset.

Compound	Observed pMIC	Predicted pMIC	Error
**12**	2.67	2.64	0.03
**11**	2.35	2.37	−0.02
**3g**	2.05	1.86	0.19
**3c**	2.01	2.08	−0.08
**3e**	1.82	1.80	0.02
**3a**	1.82	1.88	−0.06
**4d**	1.67	1.73	−0.06
**4a**	1.29	1.38	−0.10
**5**	1.24	1.18	0.07

**Table 5:** The comparison of observed and predicted values for the test dataset.

Compound	Observed pMIC	Predicted pMIC	Error
**3d**	2.20	2.00	0.20
**3f**	1.94	1.95	−0.01
**3b**	1.80	1.87	−0.07
**4e**	1.28	1.58	−0.30

## Supplementary Materials

Supplementary File 1
