# Supplementary material for: Discovery of Indeno[1,2-c]quinoline Derivatives as Potent Dual Antituberculosis and Anti-Inflammatory Agents
Source: Molecules. 2017 Jun 16;22(6):1001. doi: 10.3390/molecules22061001 (PMC6152673; doi:10.3390/molecules22061001)

H,H-6-pyrro

Mercury-400BB "Mercuryplus400"
Date: Mar 28 2012
Solvent: CDCl3
Ambient temperature
Total 32 repetitions

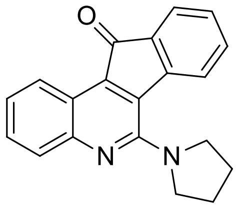

3a

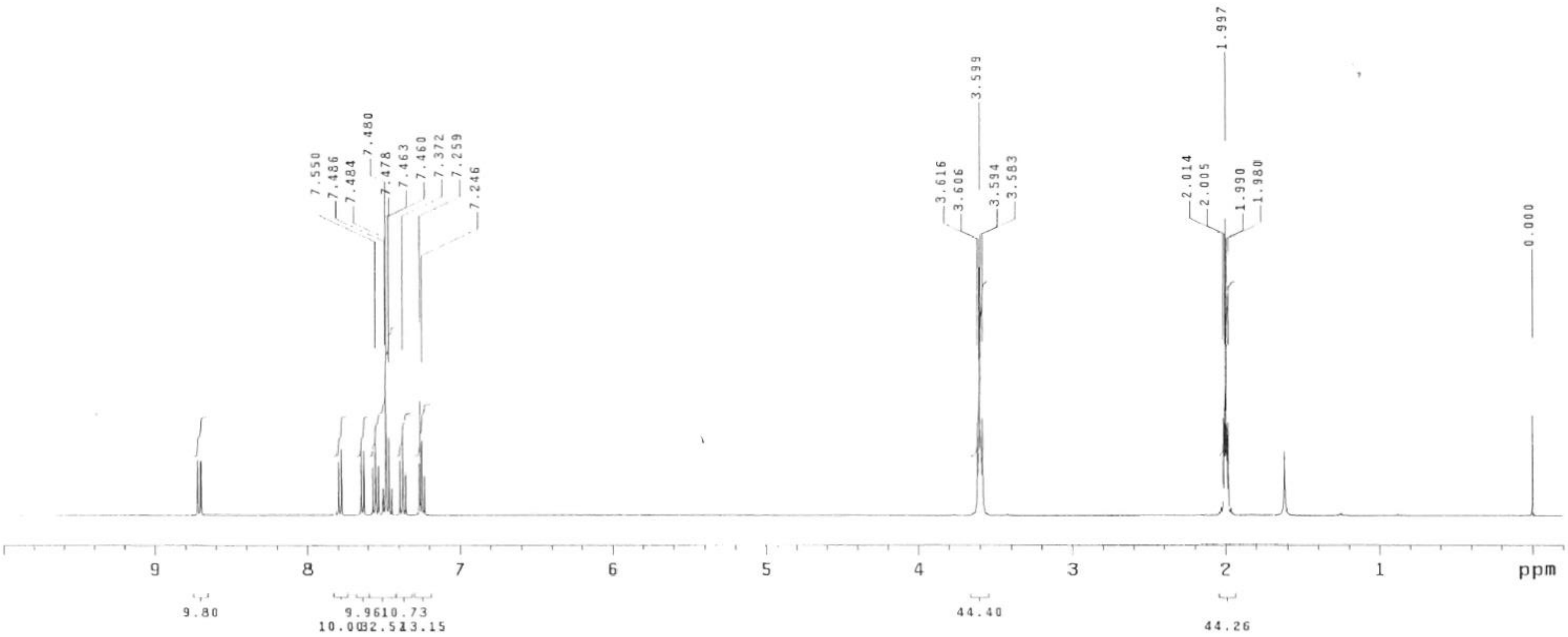

H,H-6-pyrro

Mercury-400BB "Mercuryplus400"
Date: Mar 28 2012
Solvent: CDCl3
Ambient temperature
Total 4448 repetitions

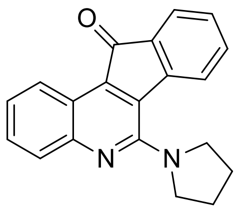

3a

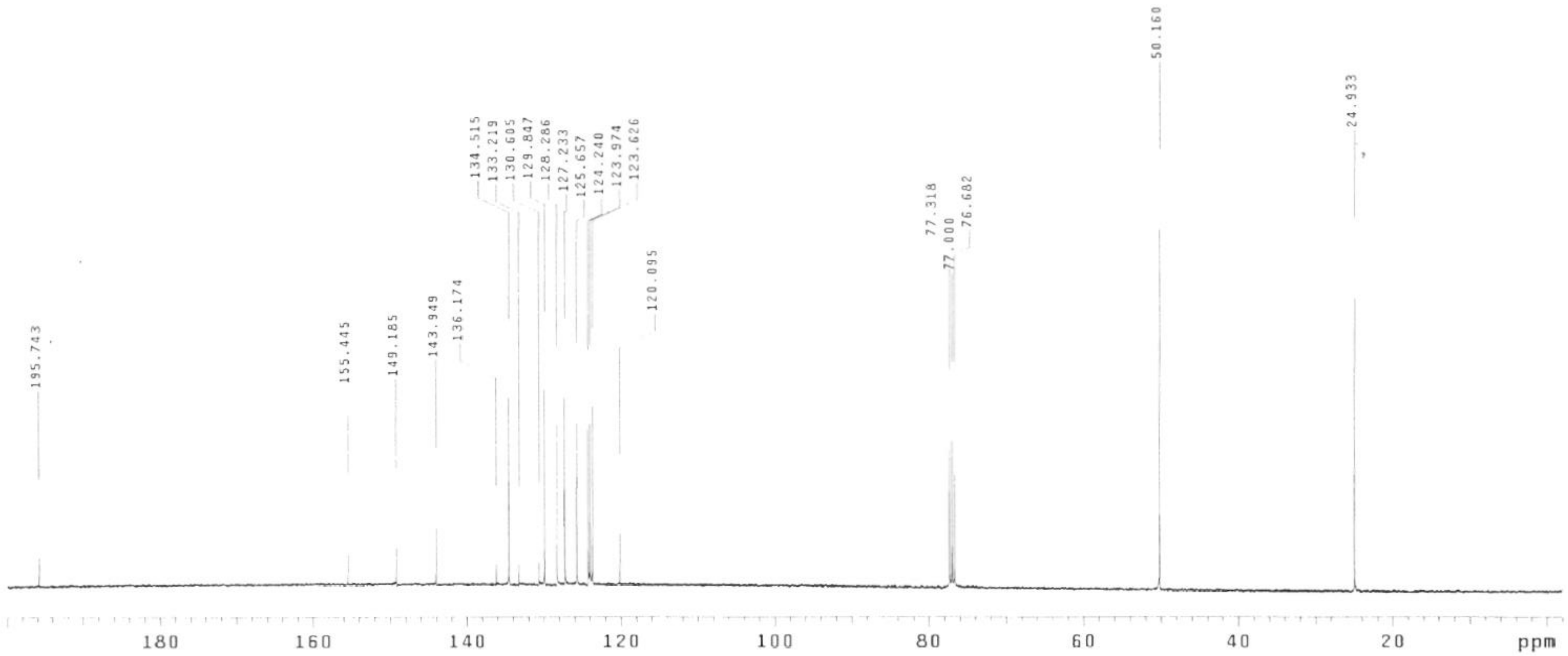

H,H-6-piperidine

Mercury-400BB "Mercuryplus400"
Date: Mar 27 2012
Solvent: CDCl3
Ambient temperature
Total 64 repetitions

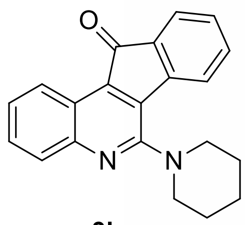

3b

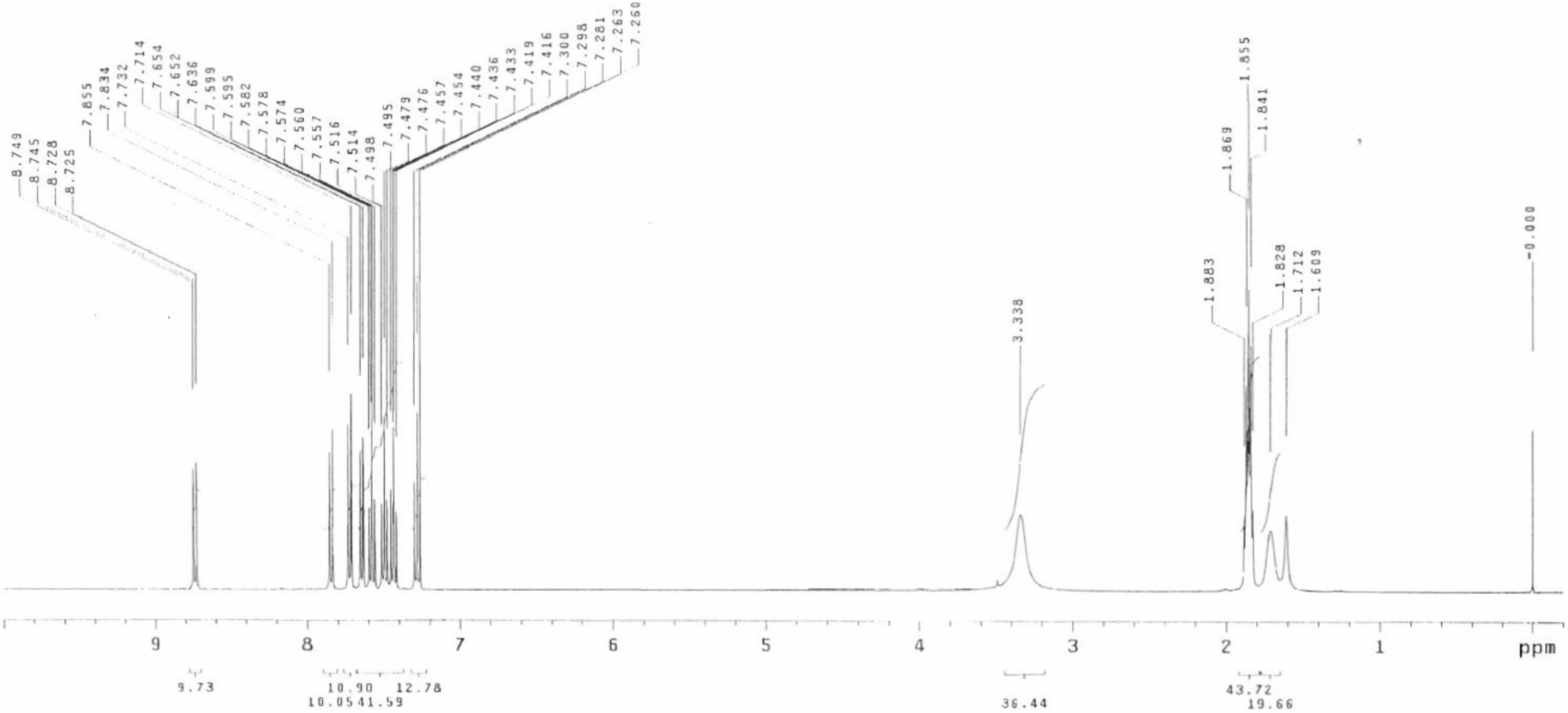

H,H-6-piperidine

Mercury-400BB "Mercuryplus400"
Date: Mar 27 2012
Solvent: CDCl3
Ambient temperature
Total 3072 repetitions

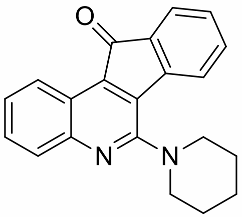

3b

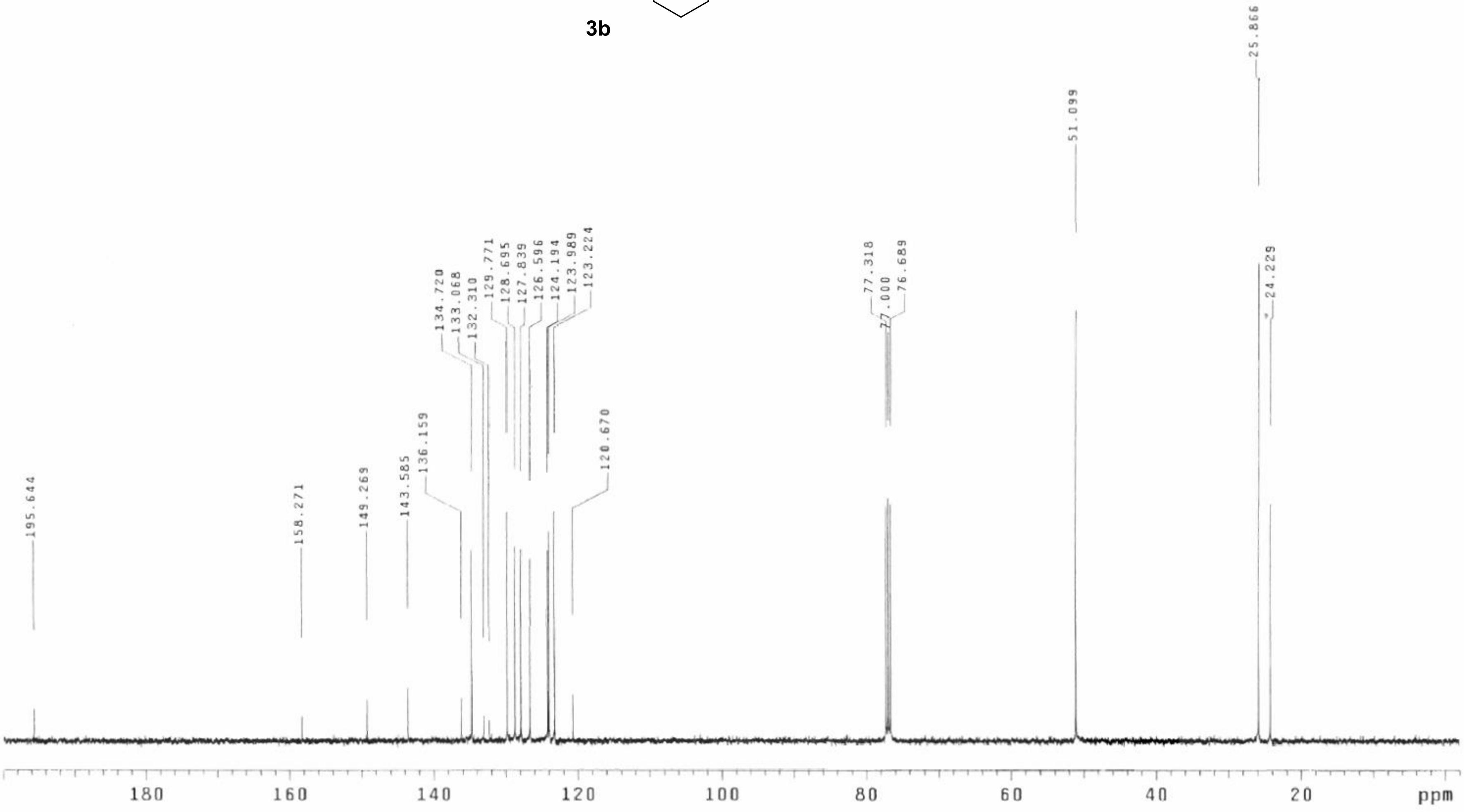

H,H-6-morphline

Mercury-400BB "Mercuryplus400"
Date: Mar 27 2012
Solvent: CDCl3
Ambient temperature
Total 64 repetitions

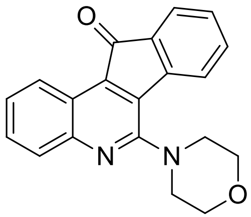

3c

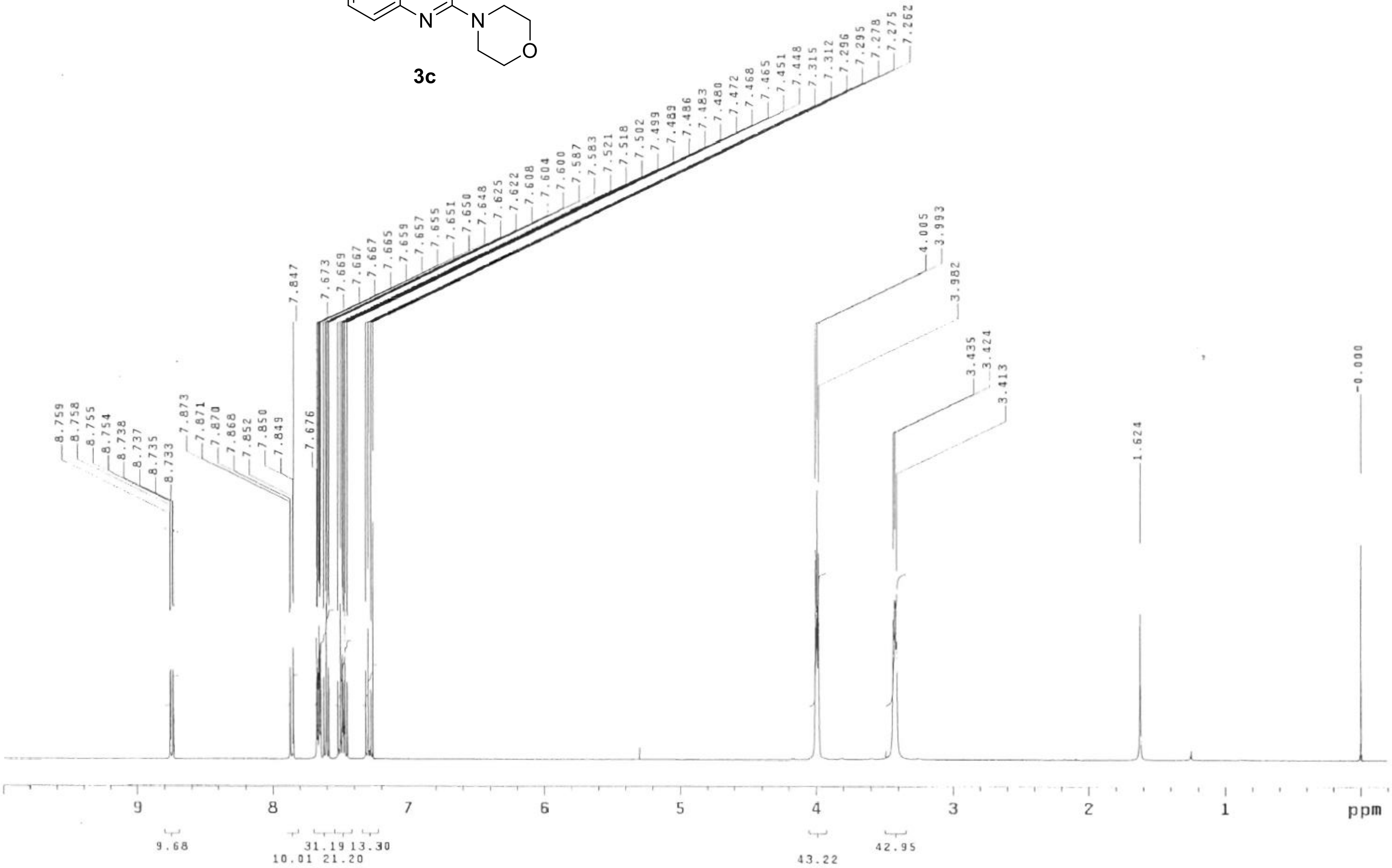

H,H-6-morphline

Mercury-400BB "Mercuryplus400"
Date: Mar 27 2012
Solvent: CDCl3
Ambient temperature
Total 1024 repetitions

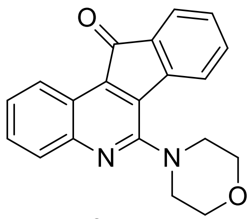

3c

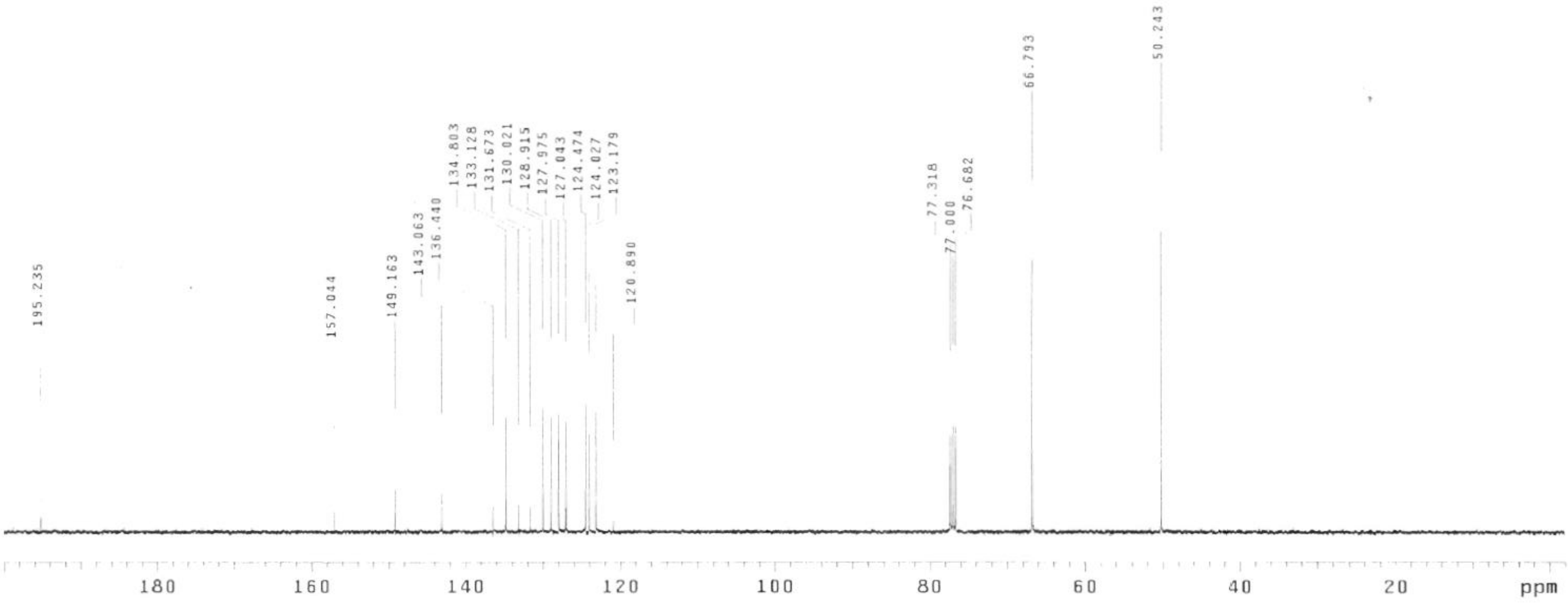

H-4-oH-piper

Mercury-400BB "Mercuryplus400"
Date: Nov 2 2007
Solvent: CDCl3
Ambient temperature
Total 32 repetitions

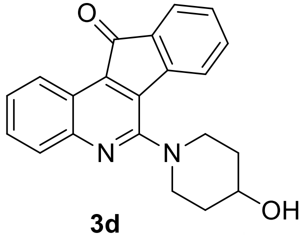

3d

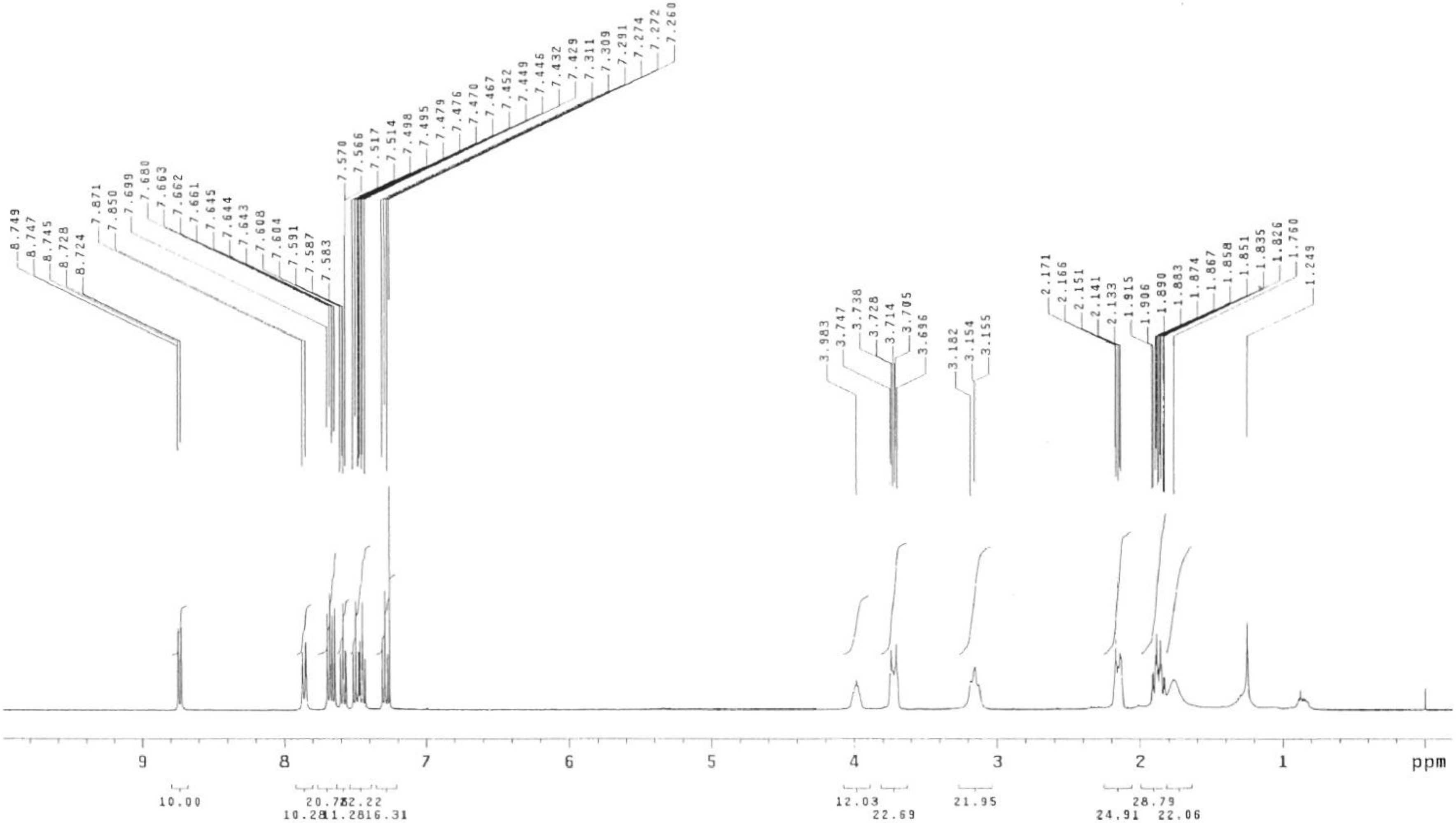

H-4-oH-piper

Mercury-400BB "Mercuryplus400"
Date: Nov 2 2007
Solvent: CDCl3
Ambient temperature
Total 2944 repetitions

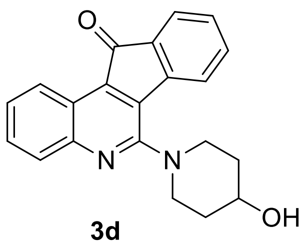

3d

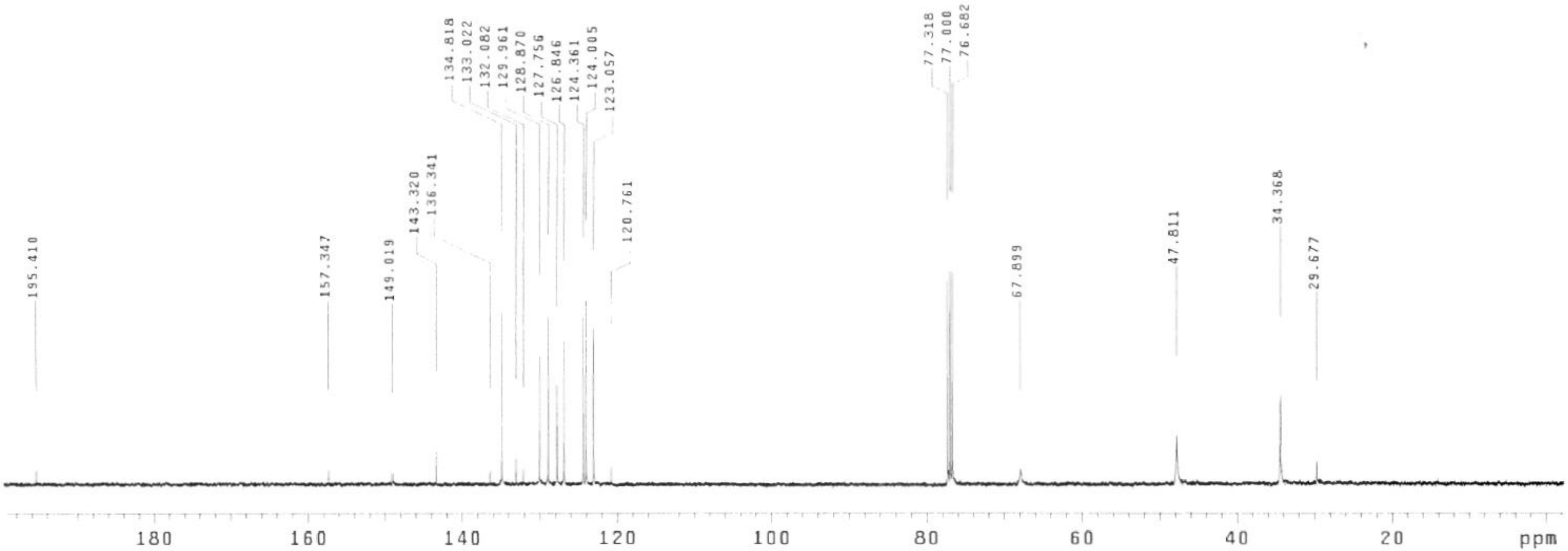

H.H-6-3OH-piper

Pulse Sequence: s2pul

UNITYplus-400 "unity400"
Date: Feb 21 2012
Solvent: DMSO
Ambient temperature
Total 64 repetitions

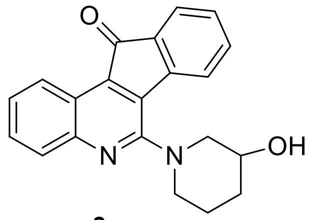

3e

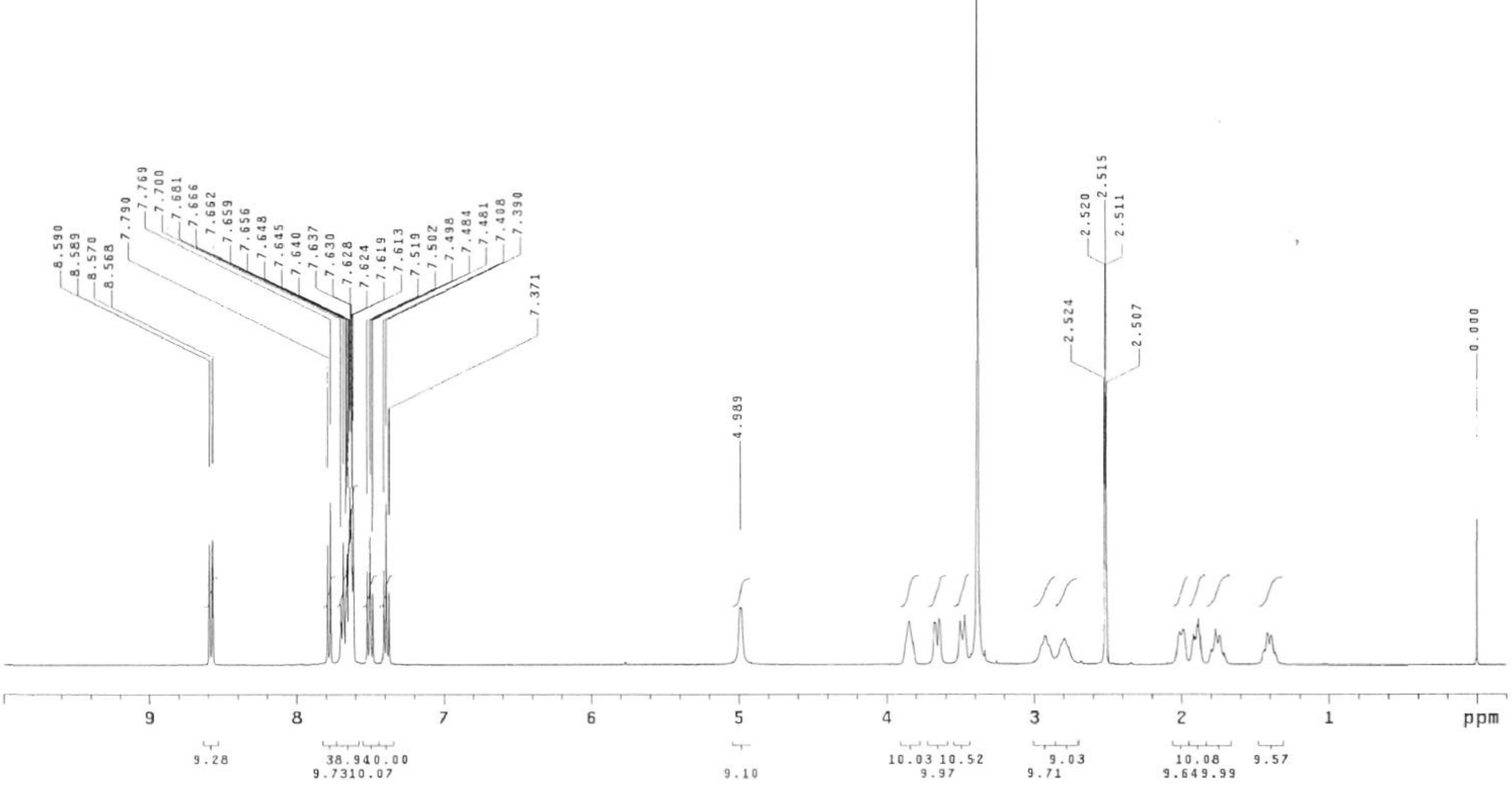

H.H-6-3OH-piper

Pulse Sequence: s2pul

UNITYplus-400 "unity400"
Date: Feb 21 2012
Solvent: DMSO
Ambient temperature
Total 4896 repetitions

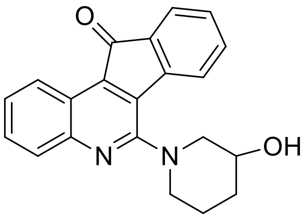

3e

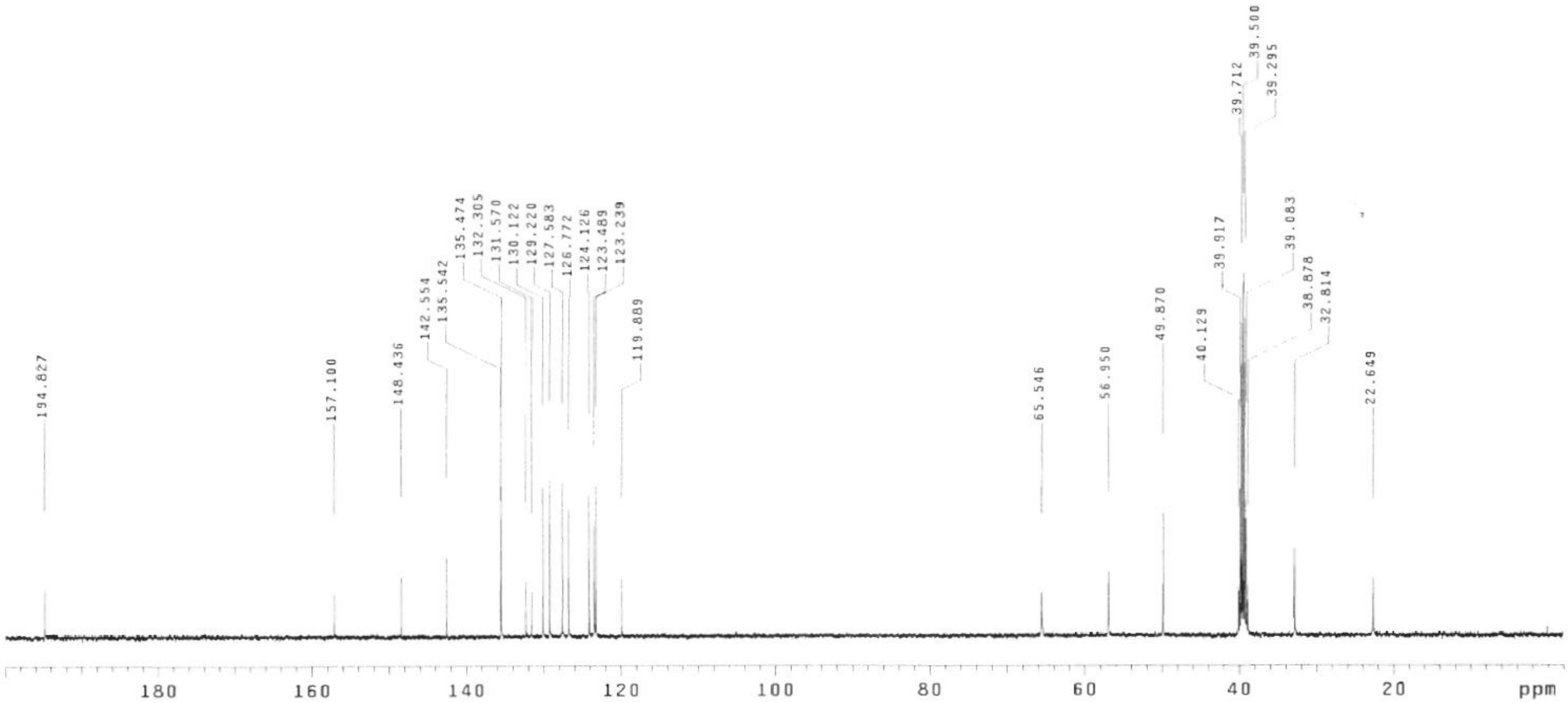

H,H-6-4-piperMeOH

Mercury-400BB "Mercuryplus400"
Date: Mar 27 2012
Solvent: CDCl3
Ambient temperature
Total 32 repetitions

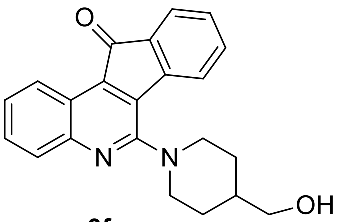

3f

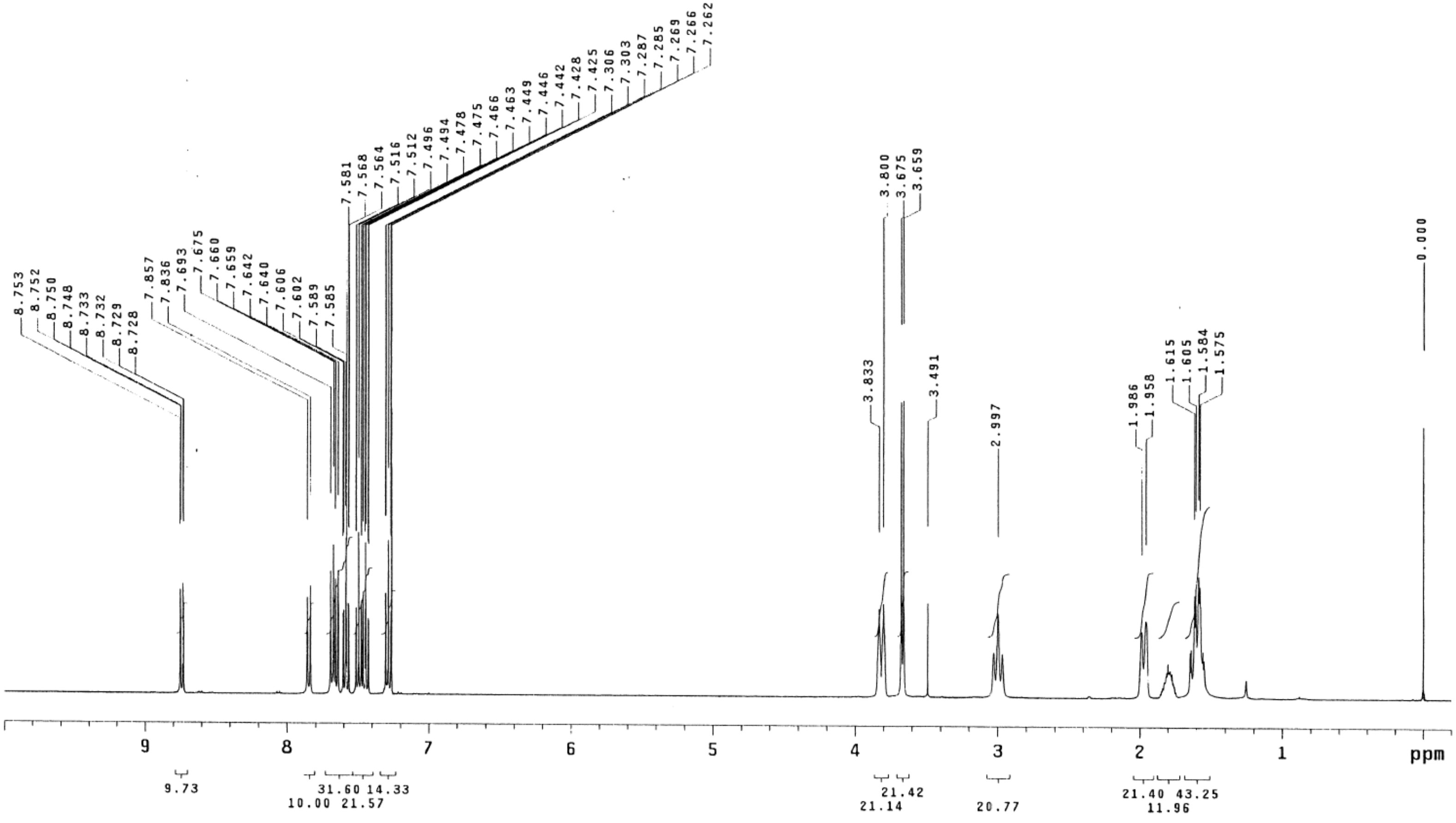

H,H-6-4-piperMeOH

Mercury-400BB "Mercuryplus400"
Date: Mar 27 2012
Solvent: CDCl3
Ambient temperature
Total 2048 repetitions

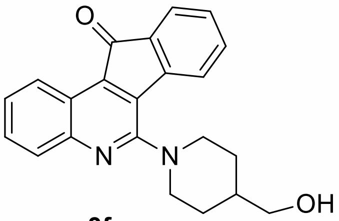

3f

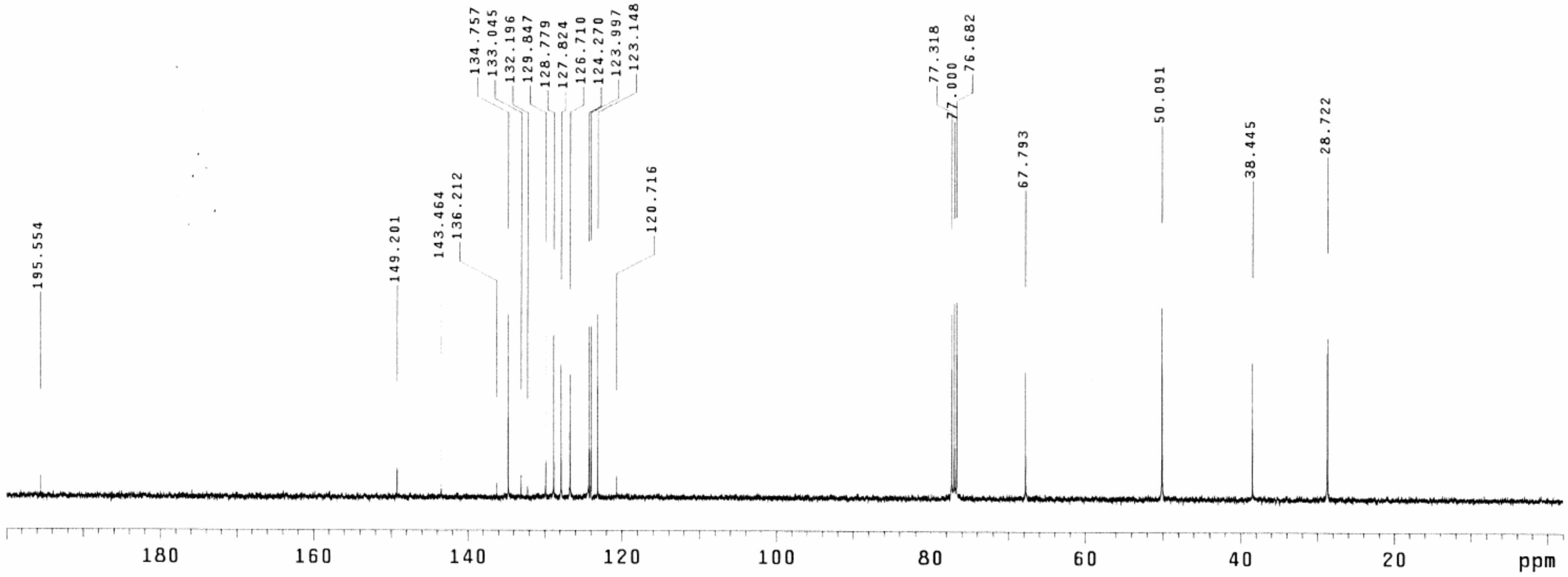

H,H-6-3-piperMeOH

Mercury-400BB "Mercuryplus400"
Date: Mar 28 2012
Solvent: CDCl3
Ambient temperature
Total 64 repetitions

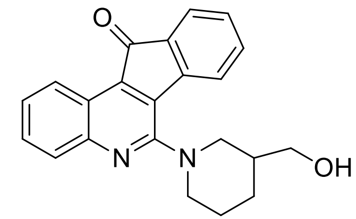

3g

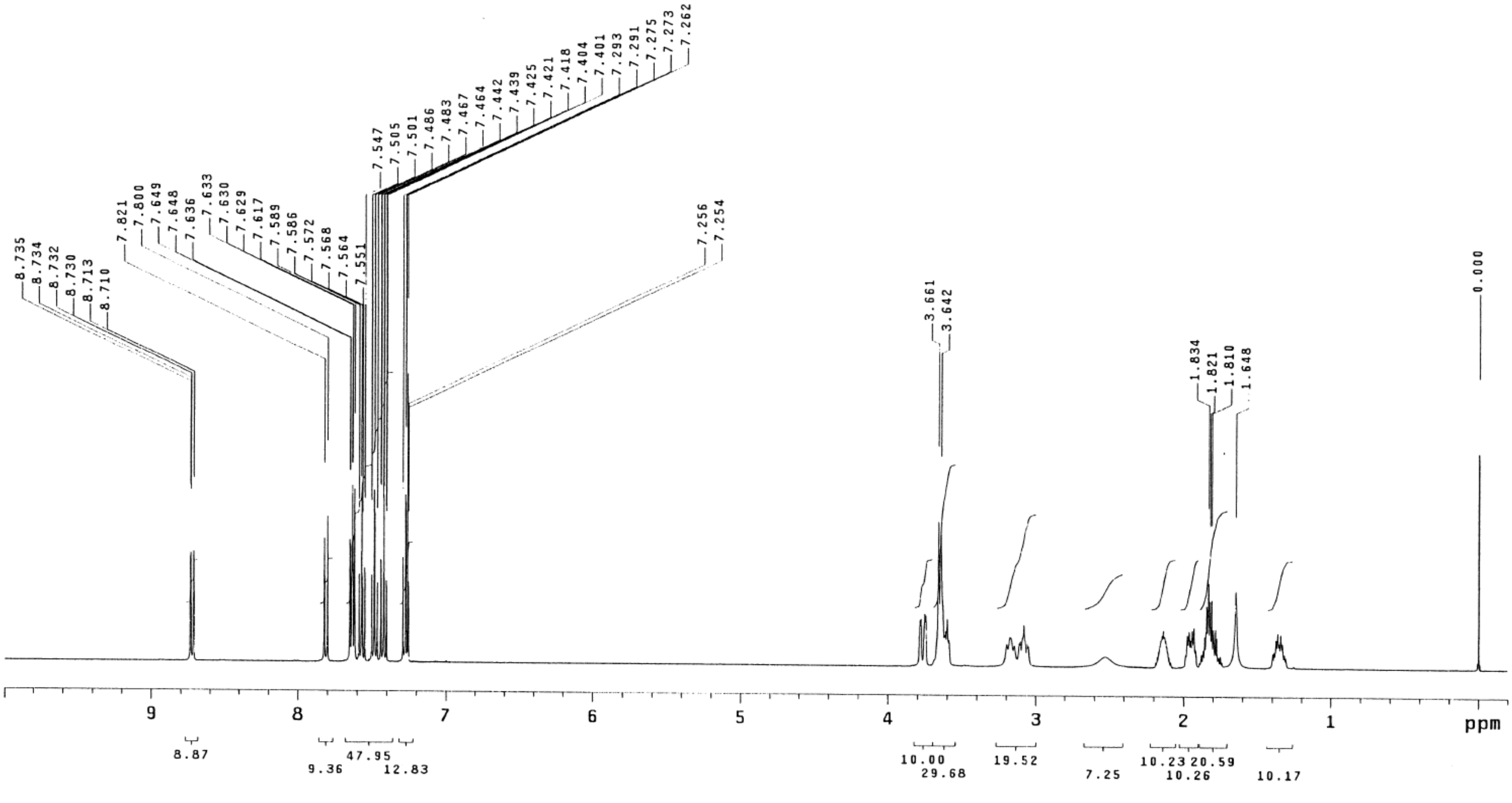

H,H-6-3-piperMeOH

Mercury-400BB "Mercuryplus400"
Date: Mar 28 2012
Solvent: CDCl3
Ambient temperature
Total 4496 repetitions

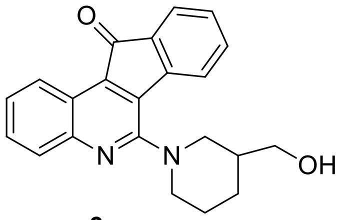

3g

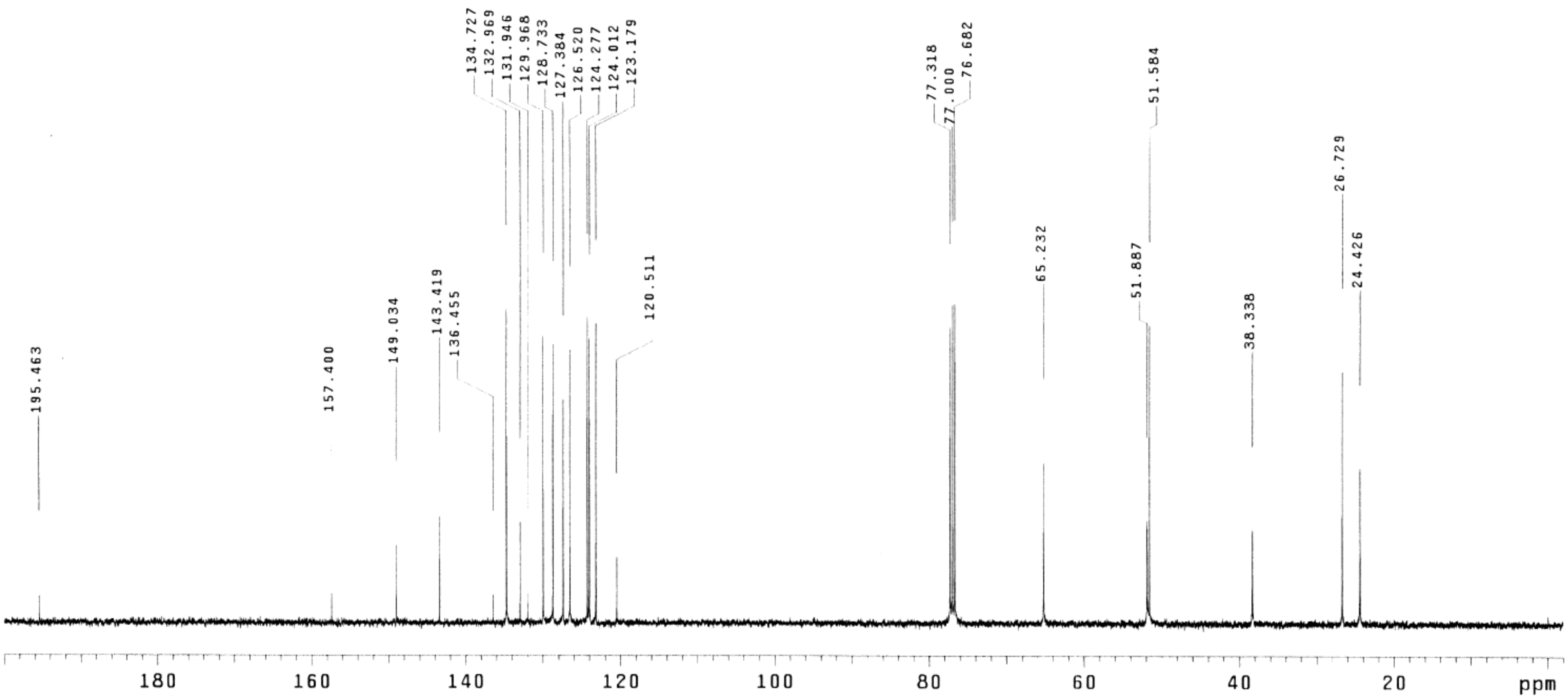

H1Pyrrolidine

Pulse Sequence: s2pul
Solvent: CDCl3
Ambient temperature
UNITYplus-400 "unityplus400"

Pulse 39.9 degrees
Acq. time 3.200 sec
Width 6000.6 Hz
32 repetitions
OBSERVE H1, 400.2874120 MHz
DATA PROCESSING
FT size 65536
Total time 1 min, 42 sec

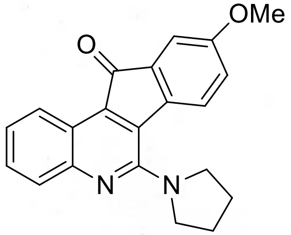

4a

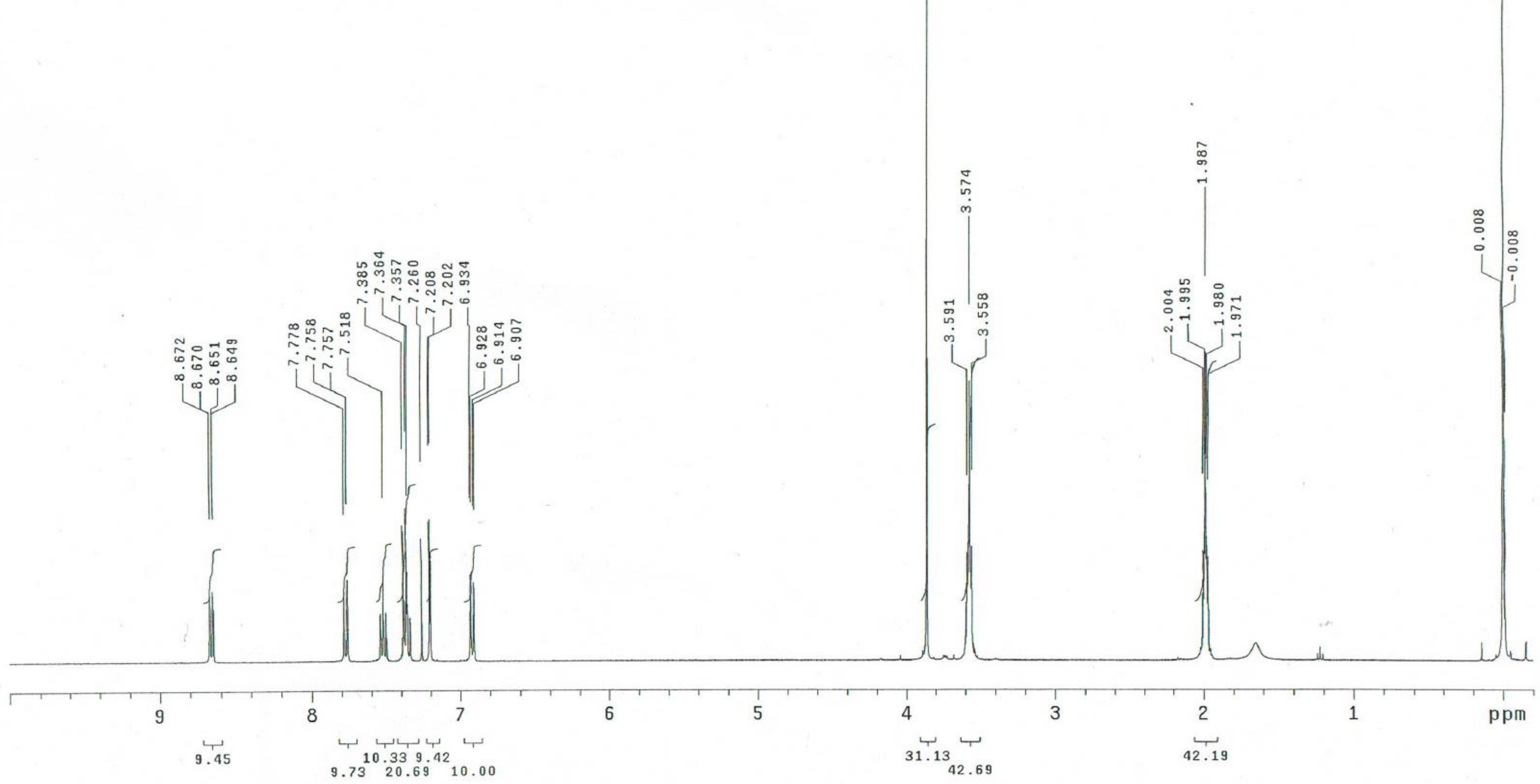

H1Pyrrolidine

Pulse Sequence: s2pul
Solvent: CDCl3
Ambient temperature
UNITYplus-400 "unityplus400"

Pulse 69.5 degrees
Acq. time 1.000 sec
Width 25000.0 Hz
4176 repetitions
OBSERVE C13, 100.6523528 MHz
DECOUPLE H1, 400.2894267 MHz
Power 44 dB
continuously on
WALTZ-16 modulated
DATA PROCESSING
Line broadening 1.0 Hz
FT size 65536
Total time 5 hr, 36 min, 56 sec

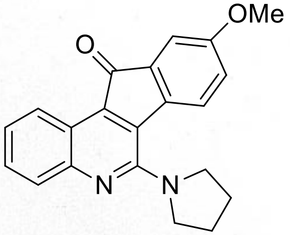

4a

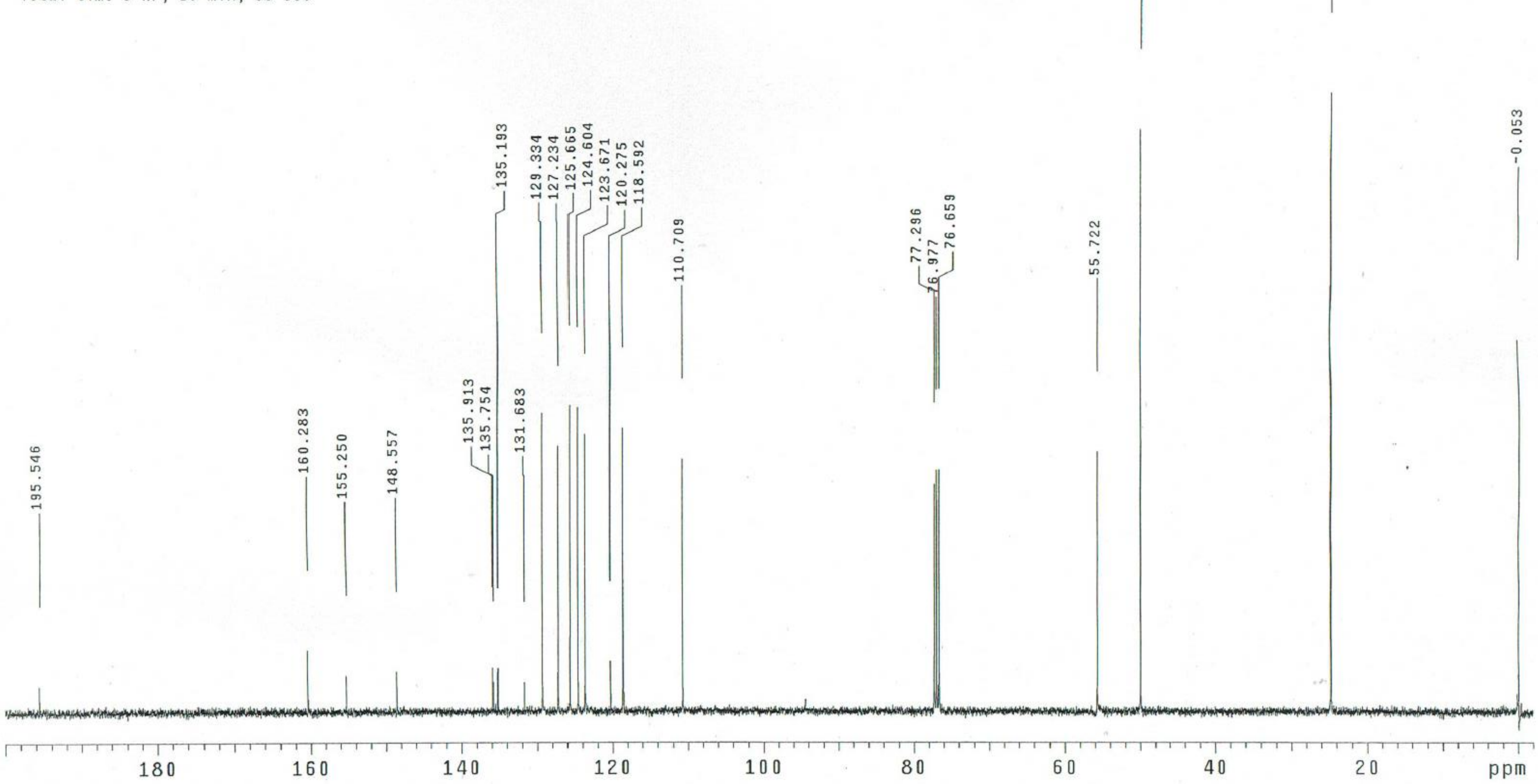

H,OMe-4OH-piper

Mercury-400BB "Mercuryplus400"
Date: Apr 12 2012
Solvent: CDCl3
Ambient temperature
Total 56 repetitions

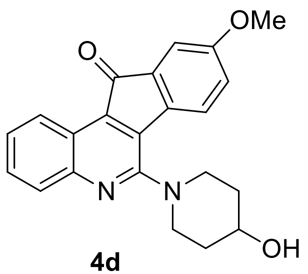

4d

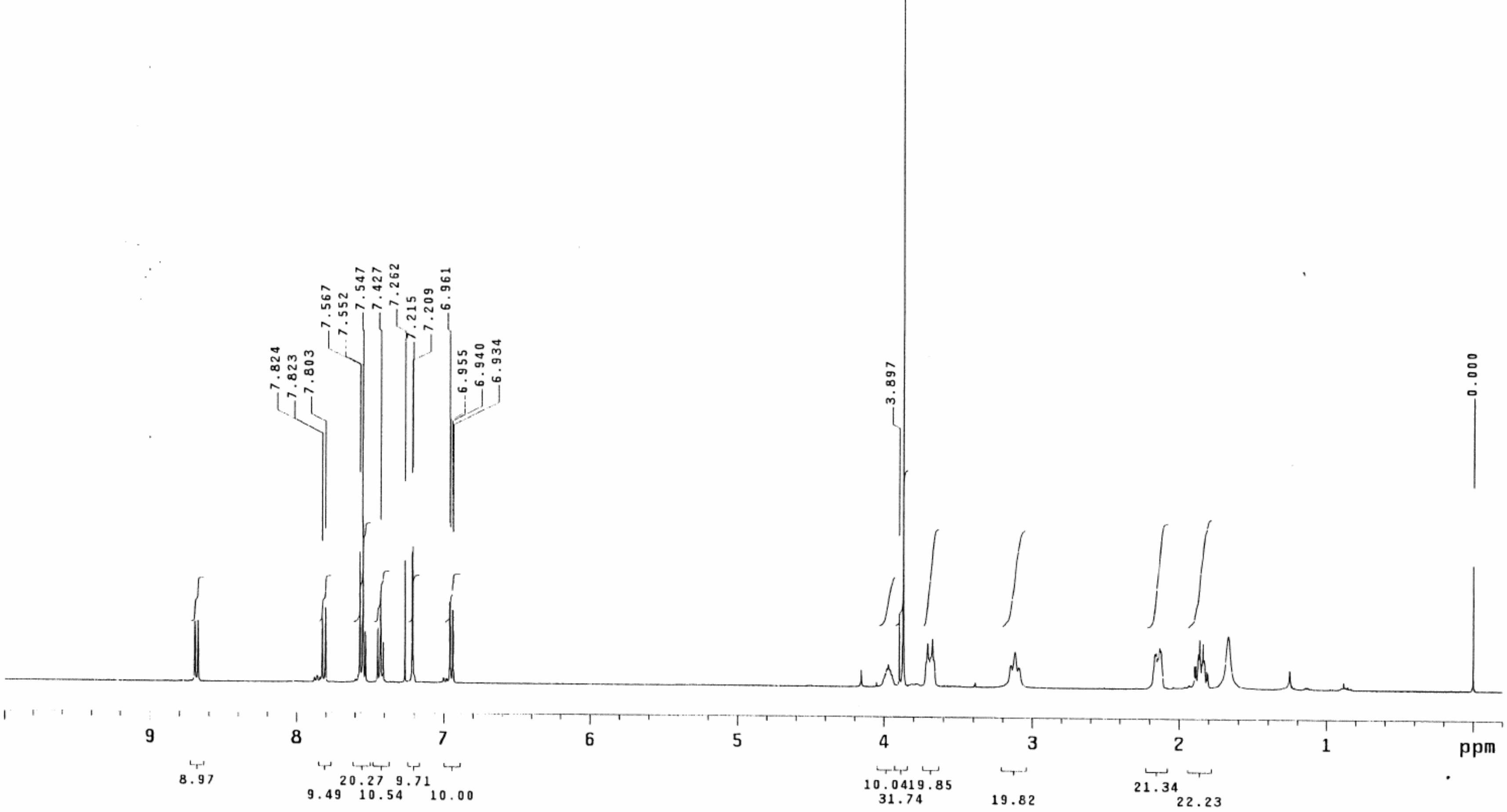

H,OMe-4OH-piper

Mercury-400BB "Mercuryplus400"
Date: Apr 12 2012
Solvent: CDCl3
Ambient temperature
Total 5280 repetitions

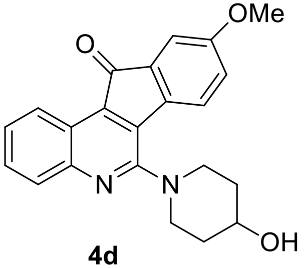

4d

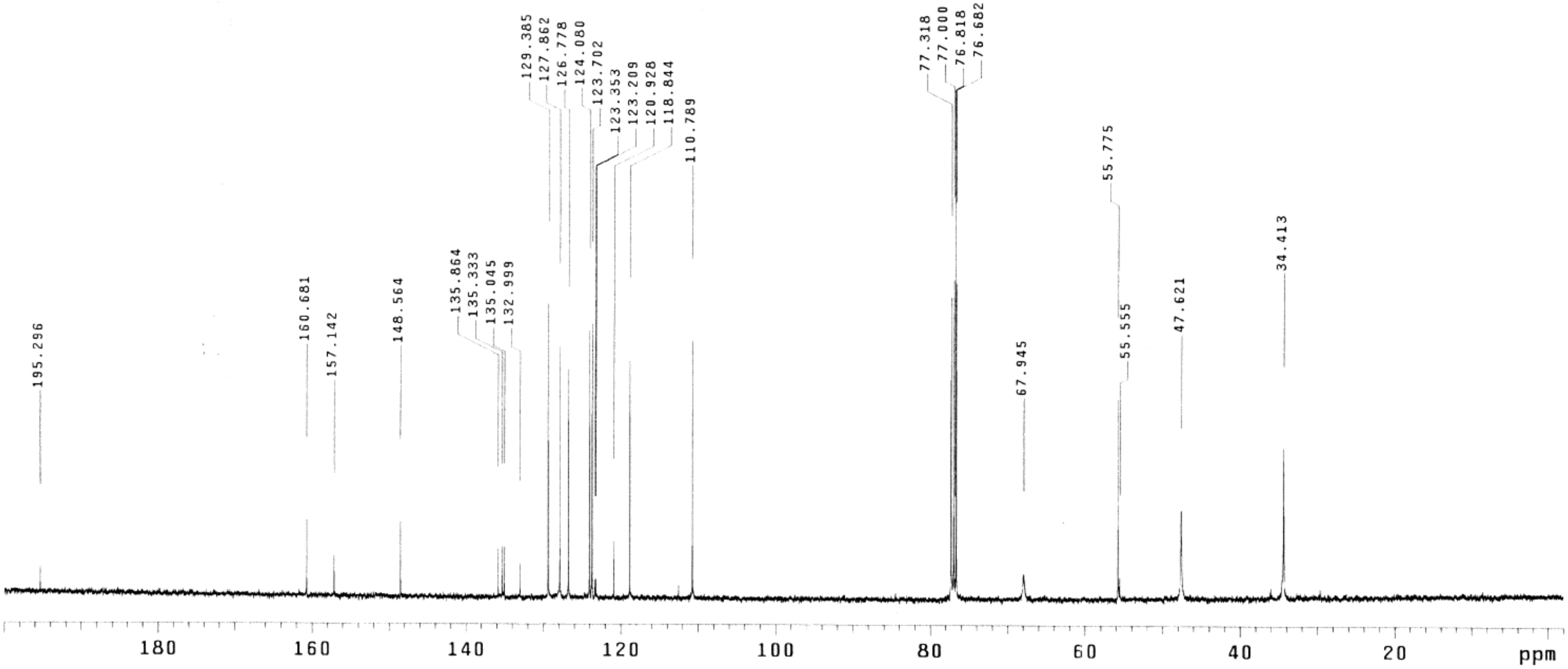

H1-3OH-PIPERID

Mercury-400BB "Mercuryplus400"
Date: Nov 2 2007
Solvent: CDCl3
Ambient temperature
Total 21 repetitions

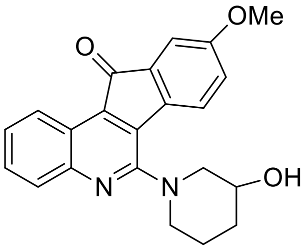

4e

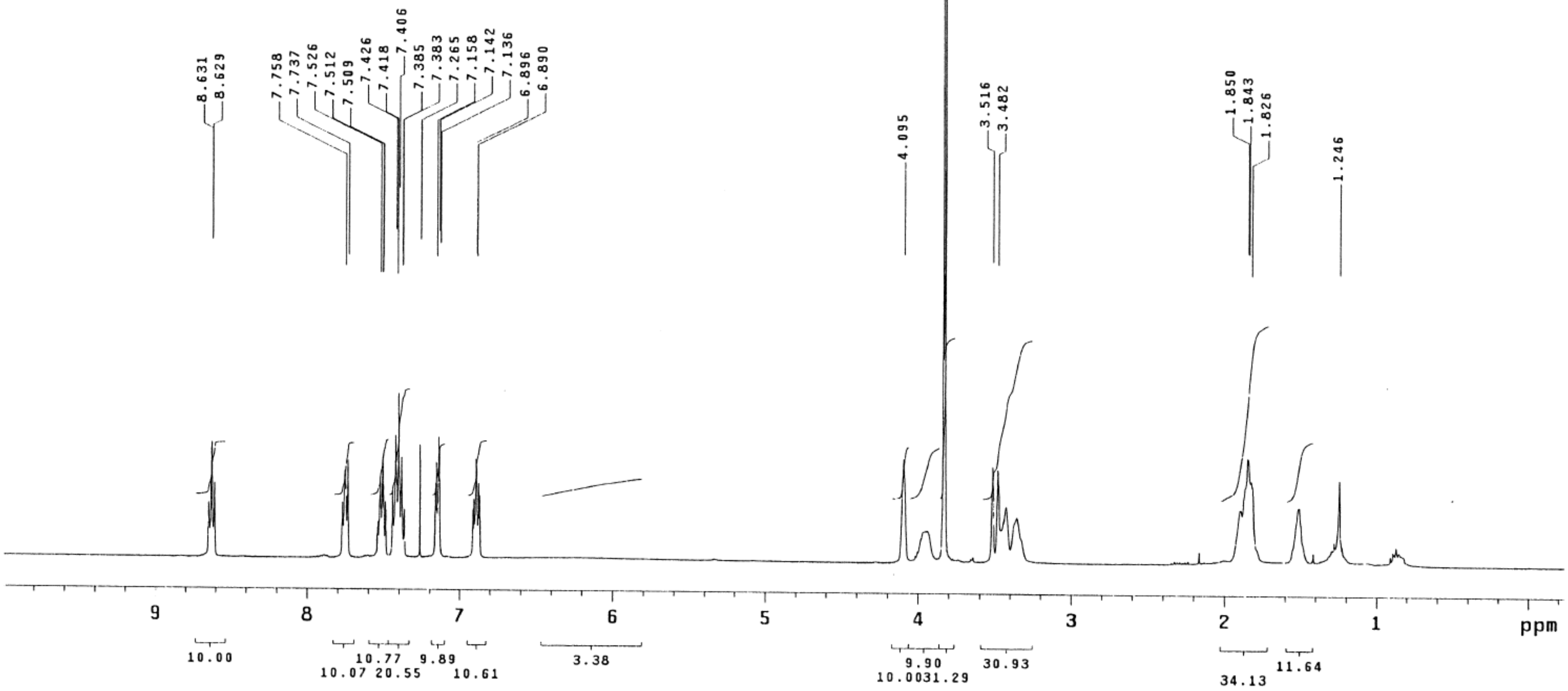

H1-3OH-PIPERID

Mercury-400BB "Mercuryplus400"
Date: Nov 2 2007
Solvent: CDCl3
Ambient temperature
Total 711 repetitions

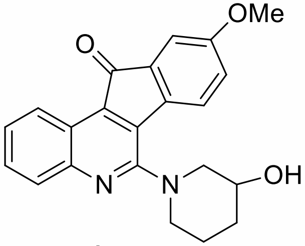

4e

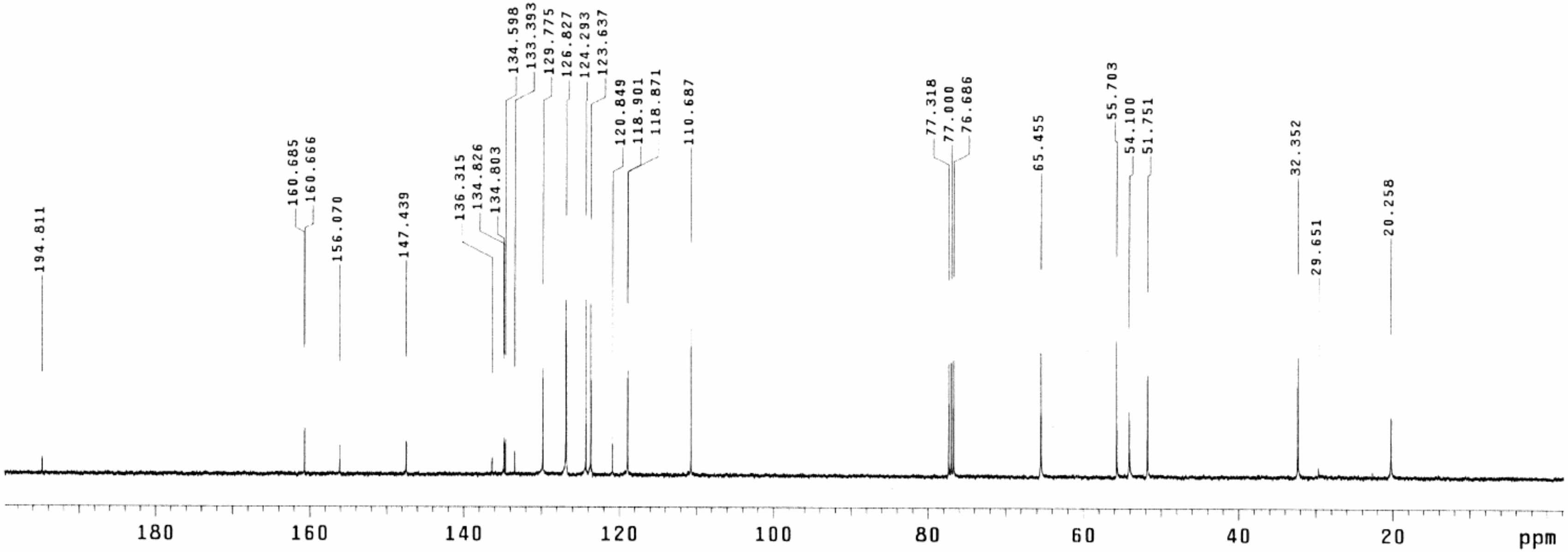

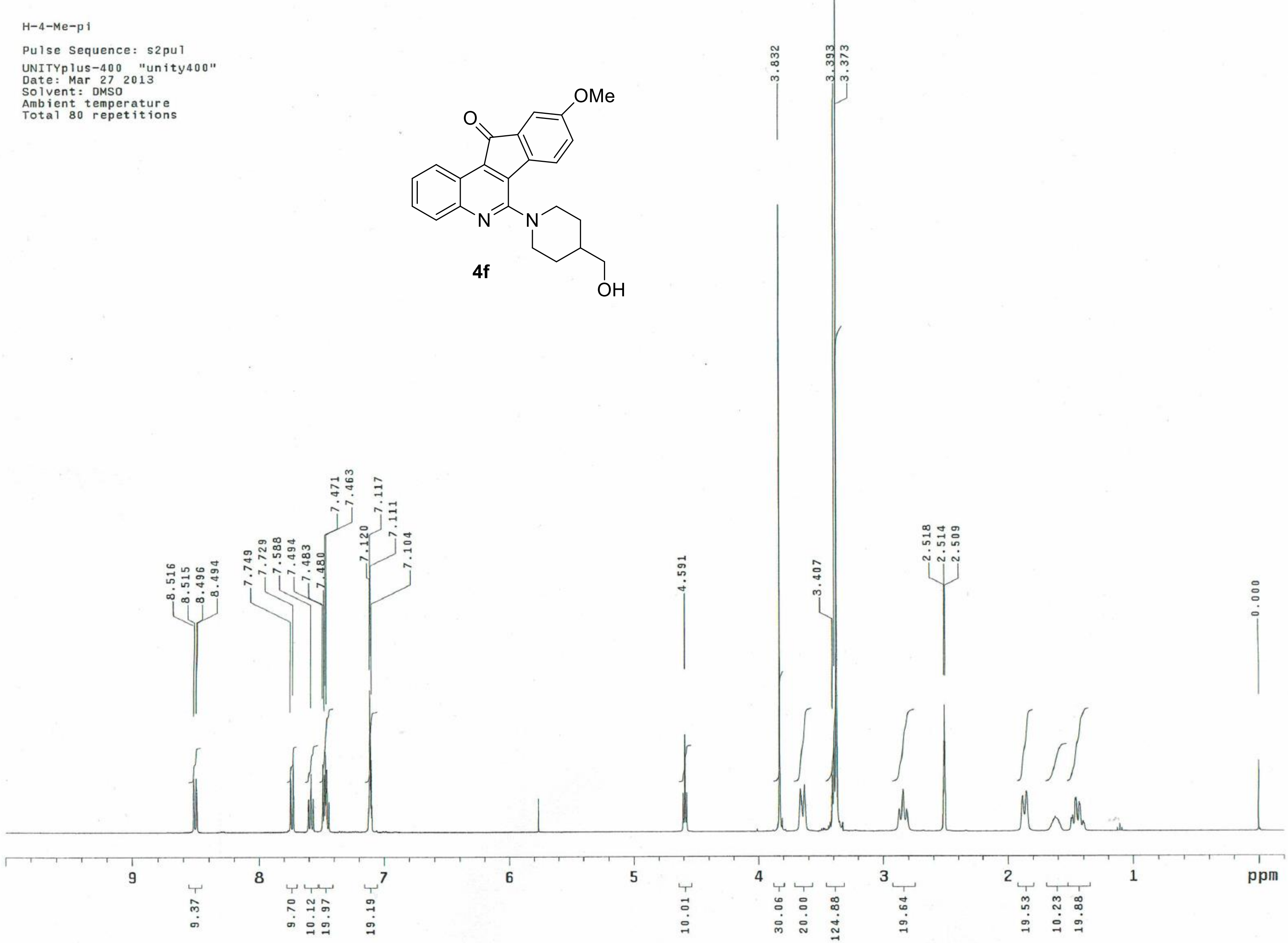
H-4-Me-pi
Pulse Sequence: s2pul
UNITYplus-400 "unity400"
Date: Mar 27 2013
Solvent: DMSO
Ambient temperature
Total 80 repetitions
OMe
O
N
N
OH
4f
3.832
3.393
3.373
8.516
8.515
8.496
8.494
7.749
7.729
7.588
7.494
7.483
7.480
7.471
7.463
7.120
7.117
7.111
7.104
4.591
3.407
2.518
2.514
2.509
0.000
9
8
7
6
5
4
3
2
1
ppm
9.37
9.70
10.12
19.97
19.19
10.01
30.06
20.00
124.88
19.64
19.53
10.23
19.88

H-4-Me-pi

Pulse Sequence: s2pul
UNITYplus-400 "unity400"
Date: Mar 27 2013
Solvent: DMSO
Ambient temperature
Total 3696 repetitions

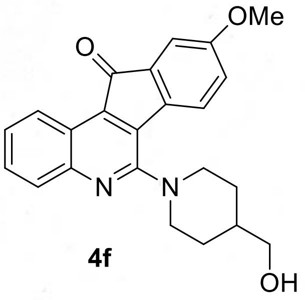

4f

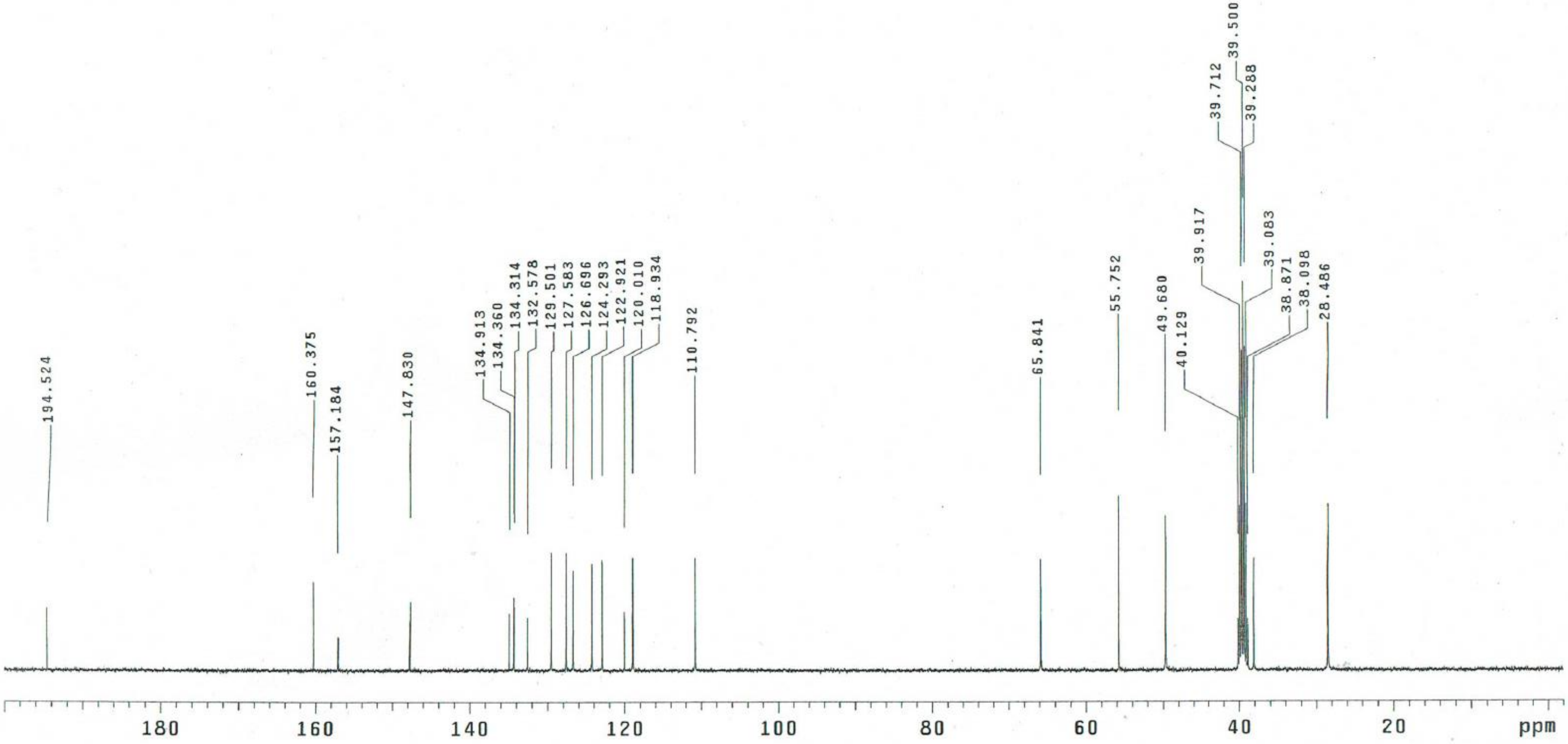

Pulse Sequence: s2pul
UNITYplus-400 "unity400"
Date: Mar 26 2013
Solvent: DMSO
Ambient temperature
Total 160 repetitions

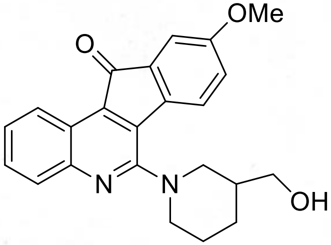

4g

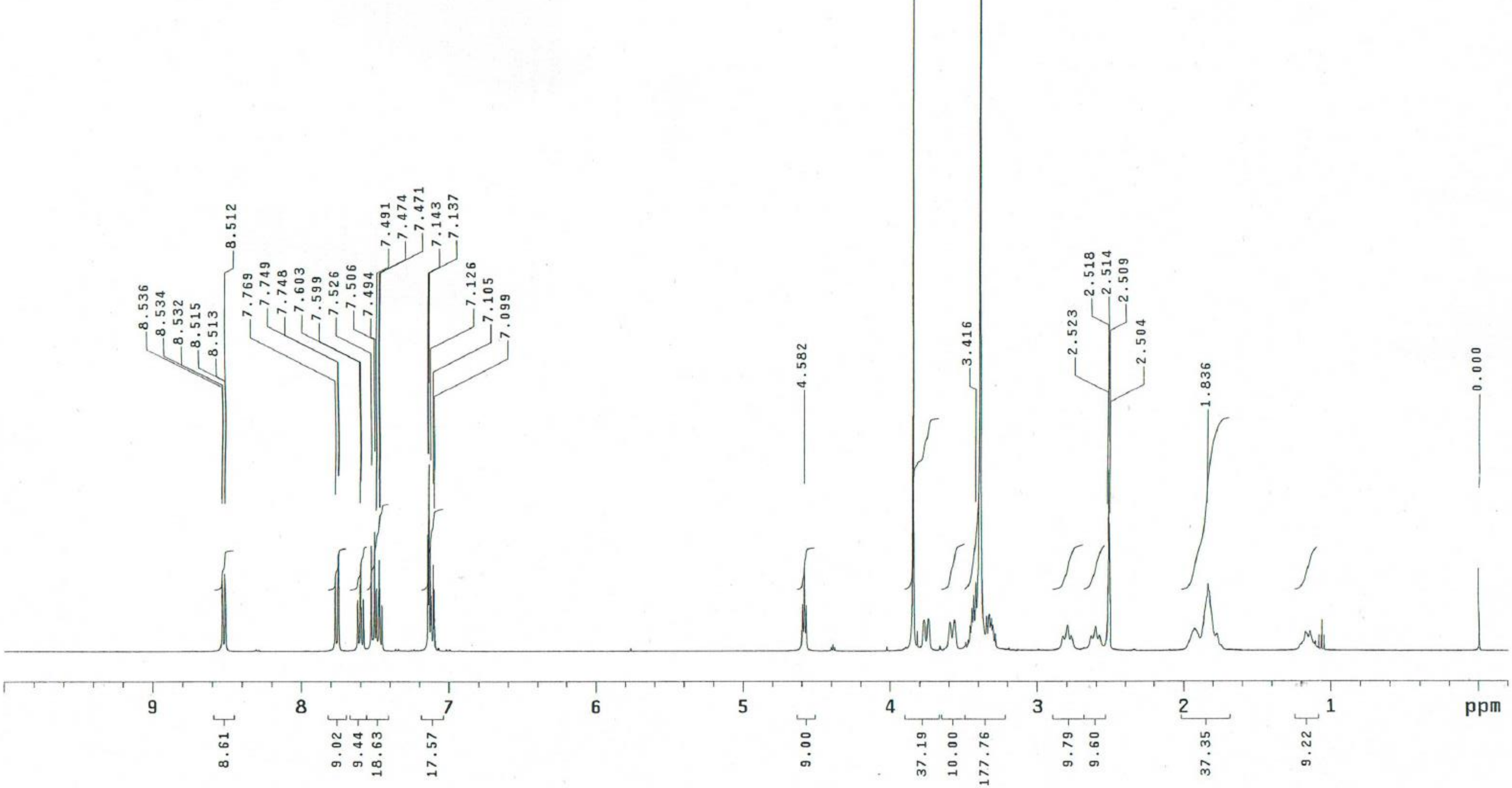

H-3-Me-pi

Pulse Sequence: s2pul
UNITYplus-400 "unity400"
Date: Mar 26 2013
Solvent: DMSO
Ambient temperature
Total 64000 repetitions

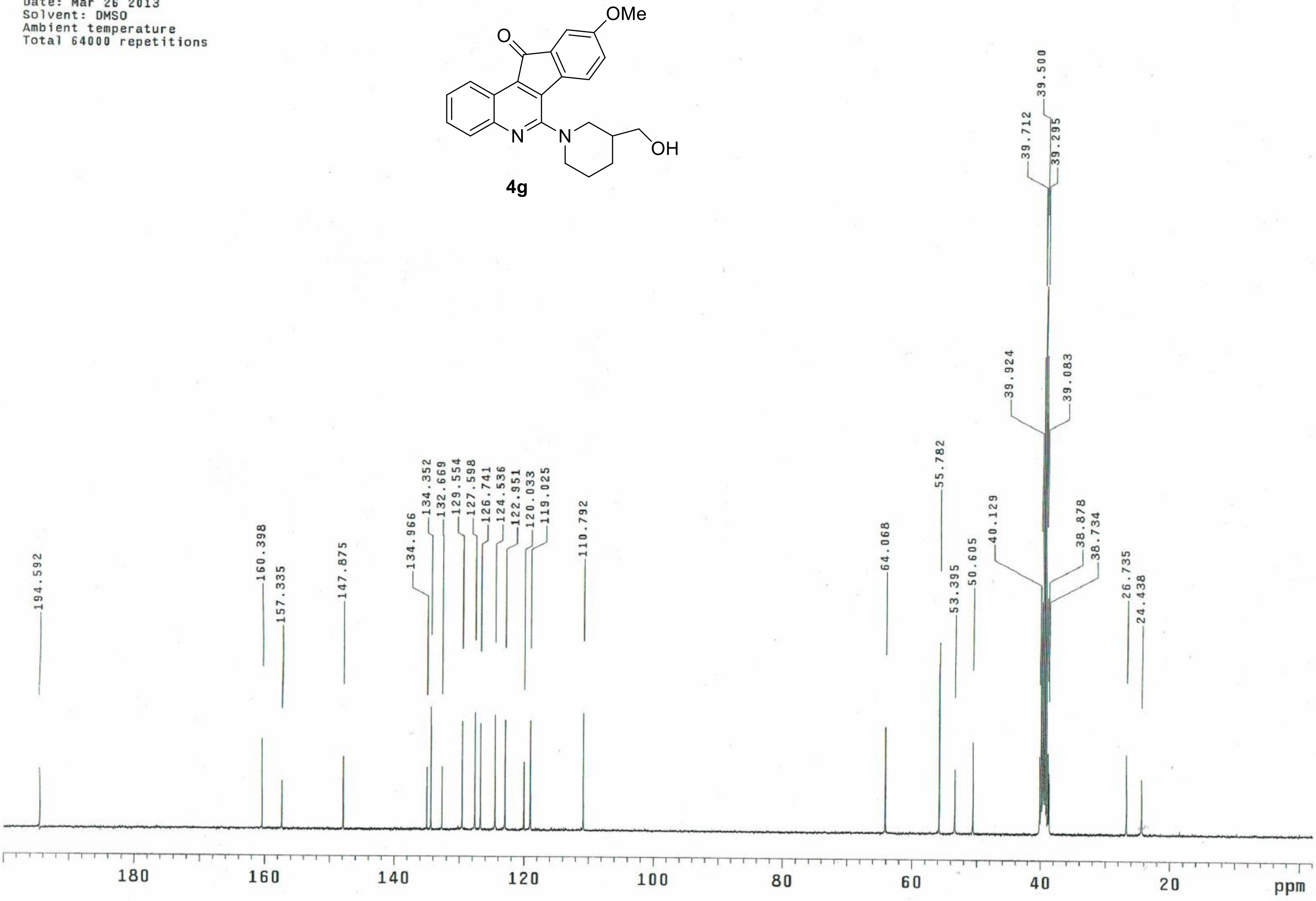

TCH-RE

Pulse Sequence: s2pul
Solvent: CDCl3
Ambient temperature
Mercury-400BB "MercuryPlus400"

Pulse 48.1 degrees
Acq. time 4.002 sec
Width 5995.2 Hz
32 repetitions
OBSERVE H1, 400.3978991 MHz
DATA PROCESSING
FT size 65536
Total time 2 min, 33 sec

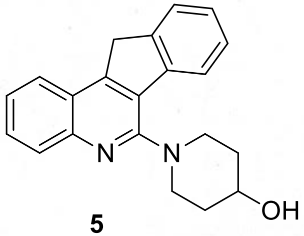

5

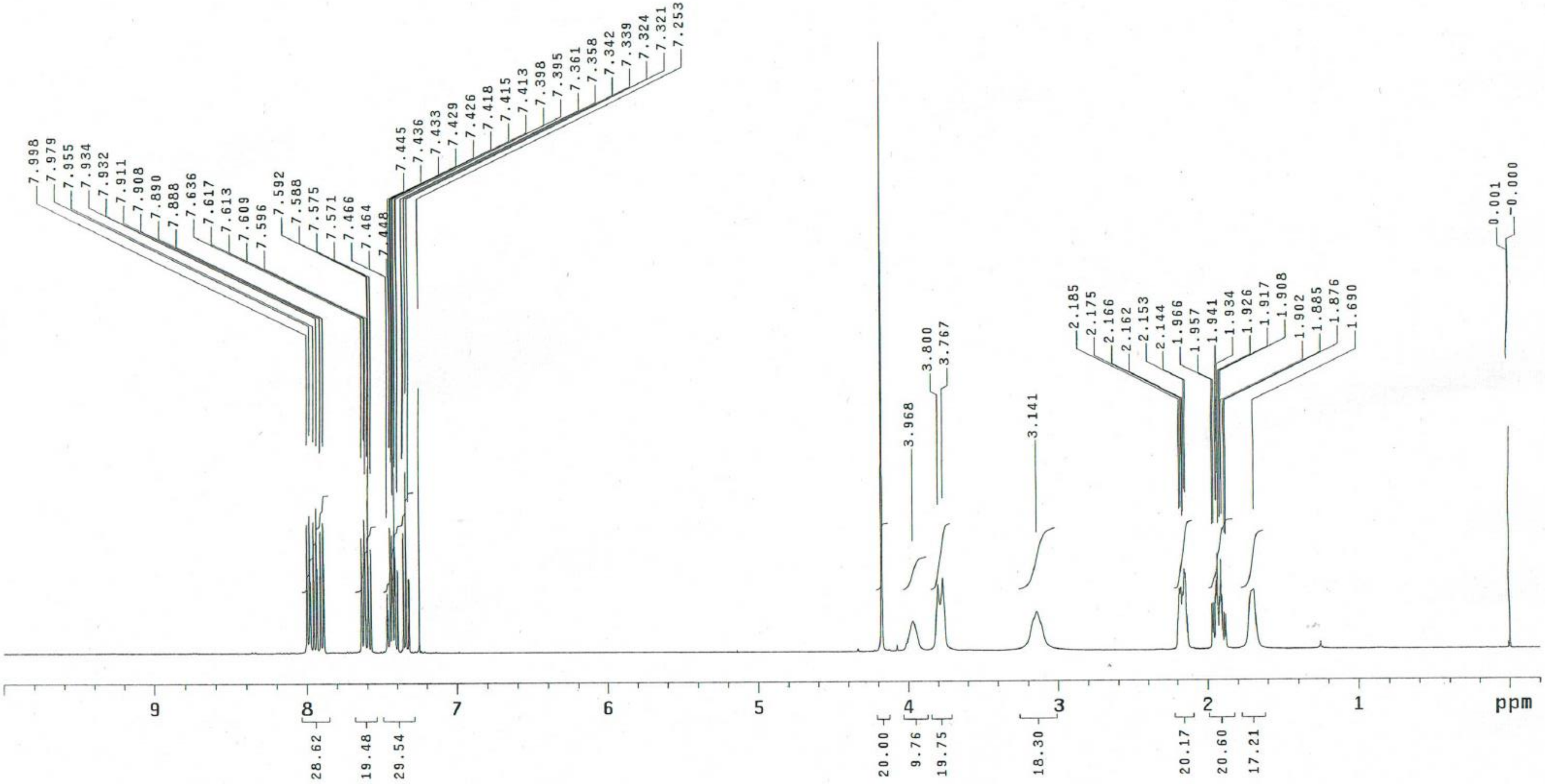

TCH-RE

Pulse Sequence: s2pul

Solvent: CDCl3
Ambient temperature
Mercury-400BB "MercuryPlus400"

Pulse 68.7 degrees
Acq. time 1.000 sec
Width 25000.0 Hz
5552 repetitions
OBSERVE C13, 100.6801338 MHz
DECOUPLE H1, 400.3999572 MHz
Power 38 dB
continuously on
WALTZ-16 modulated
DATA PROCESSING
Line broadening 1.0 Hz
FT size 65536
Total time 21 hr, 51 min, 34 sec

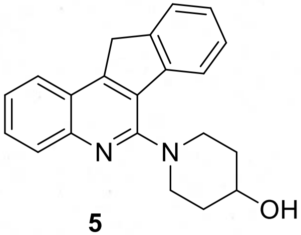

5

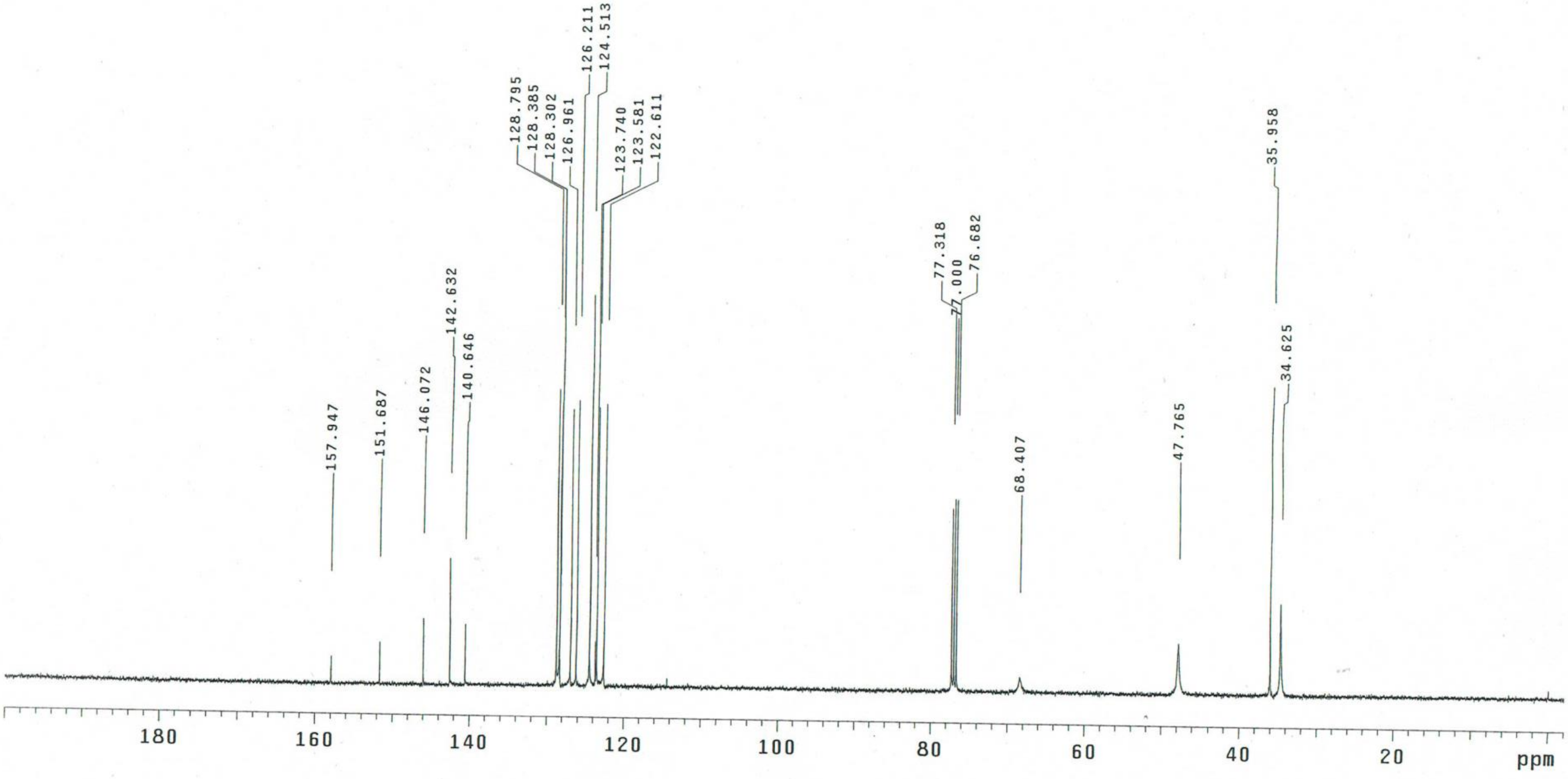

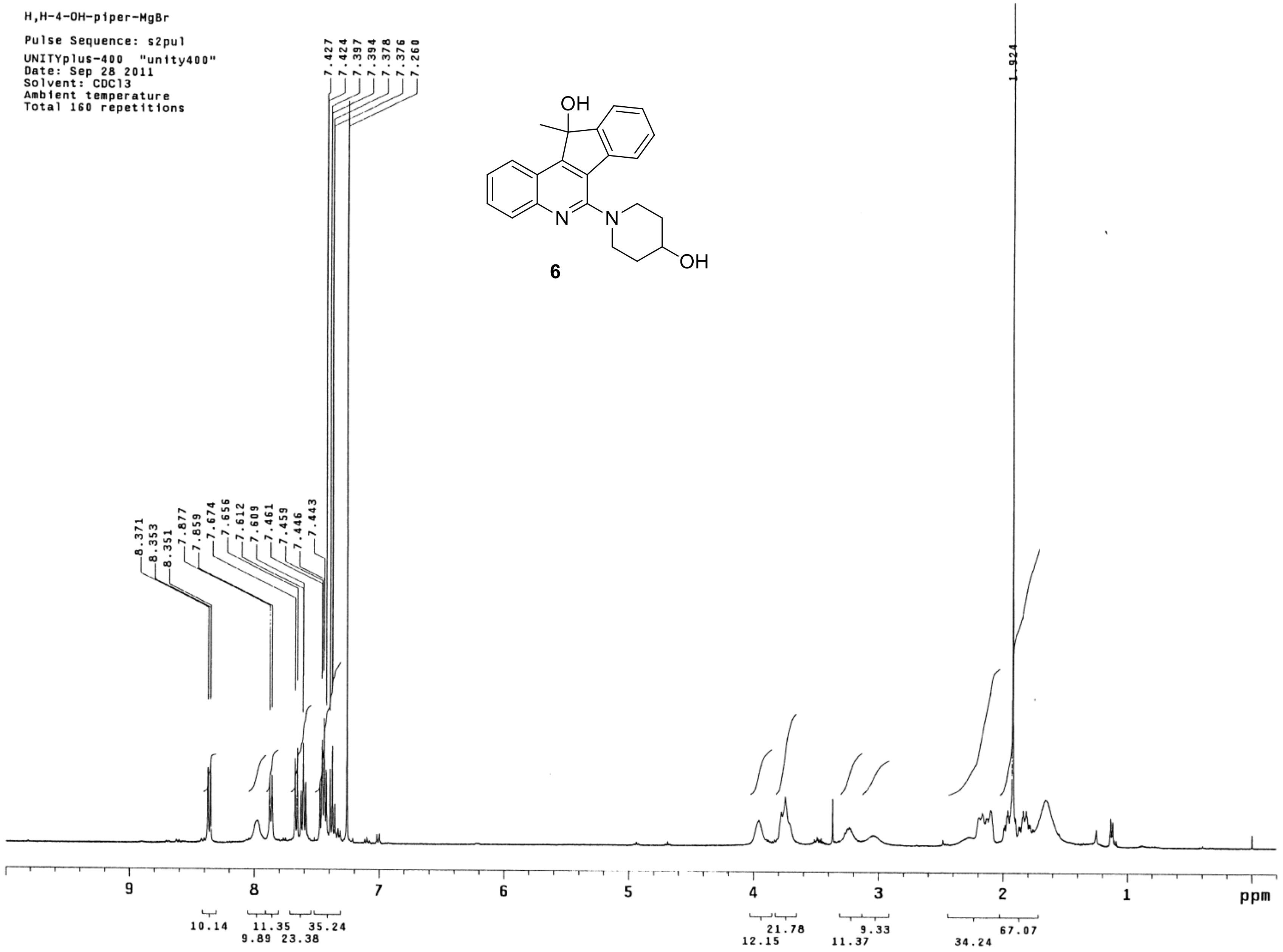
H,H-4-OH-piper-MgBr
Pulse Sequence: s2pul
UNITYplus-400 "unity400"
Date: Sep 28 2011
Solvent: CDCl3
Ambient temperature
Total 160 repetitions
7.427
7.424
7.397
7.394
7.378
7.376
7.260
1.924
OH
N
N
OH
6
8.371
8.353
8.351
7.877
7.859
7.674
7.656
7.612
7.609
7.461
7.459
7.446
7.443
9
8
7
6
5
4
3
2
1
ppm
10.14
11.35
35.24
9.89
23.38
21.78
12.15
9.33
11.37
67.07
34.24

H,H-4-OH-piper-MgBr

Pulse Sequence: s2pul

UNITYplus-400 "unity400"
Date: Sep 27 2011
Solvent: CDCl3
Ambient temperature
Total 40624 repetitions

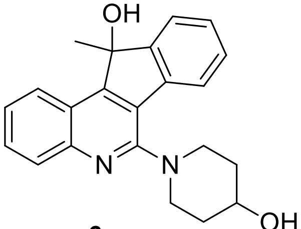

6

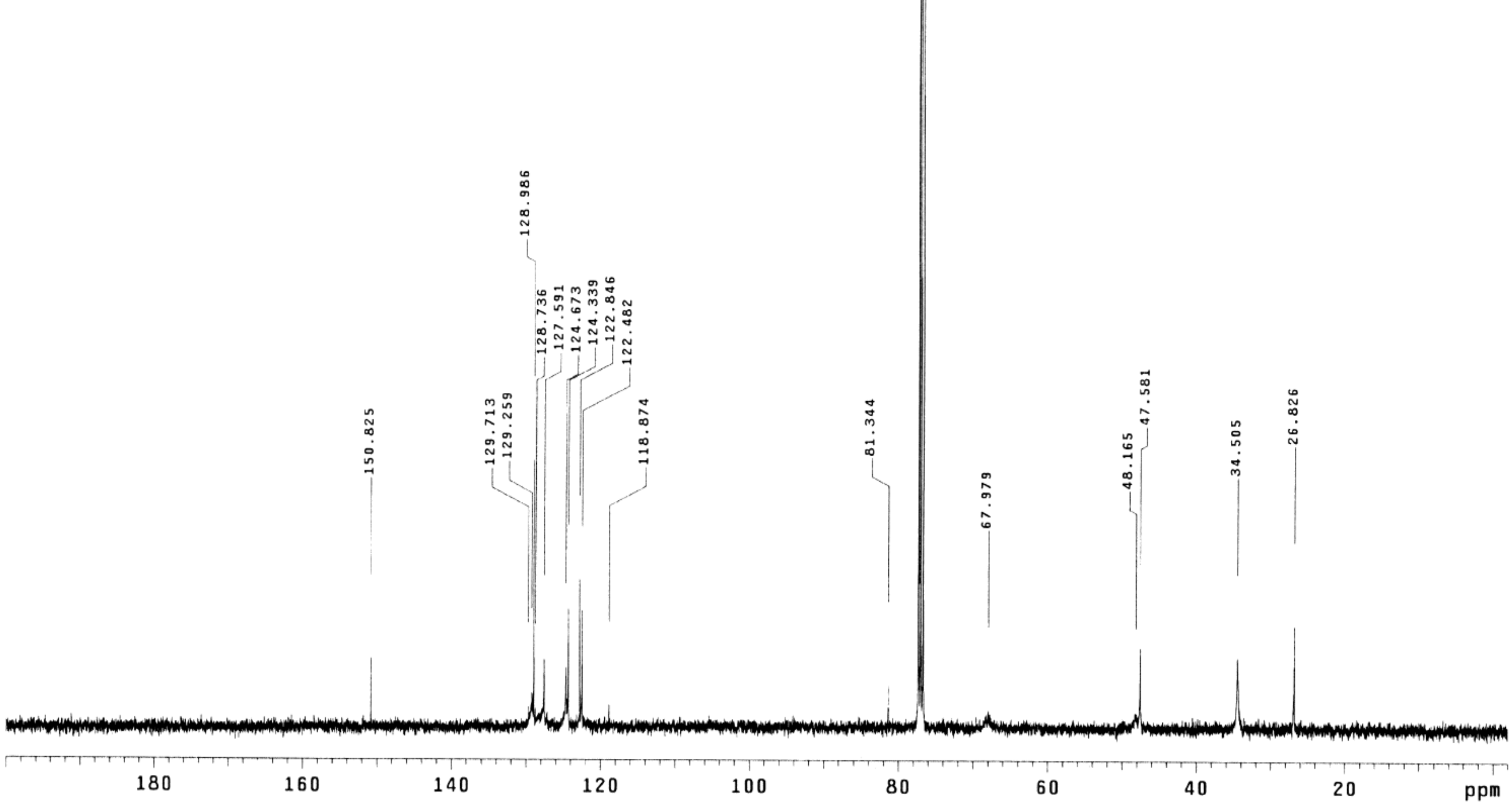

H,H-NOH

Pulse Sequence: s2pul
UNITYplus-400 "unity400"
Date: Oct 12 2011
Solvent: DMSO
Ambient temperature
Total 64 repetitions

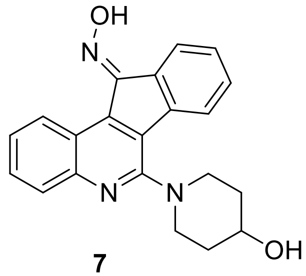

7

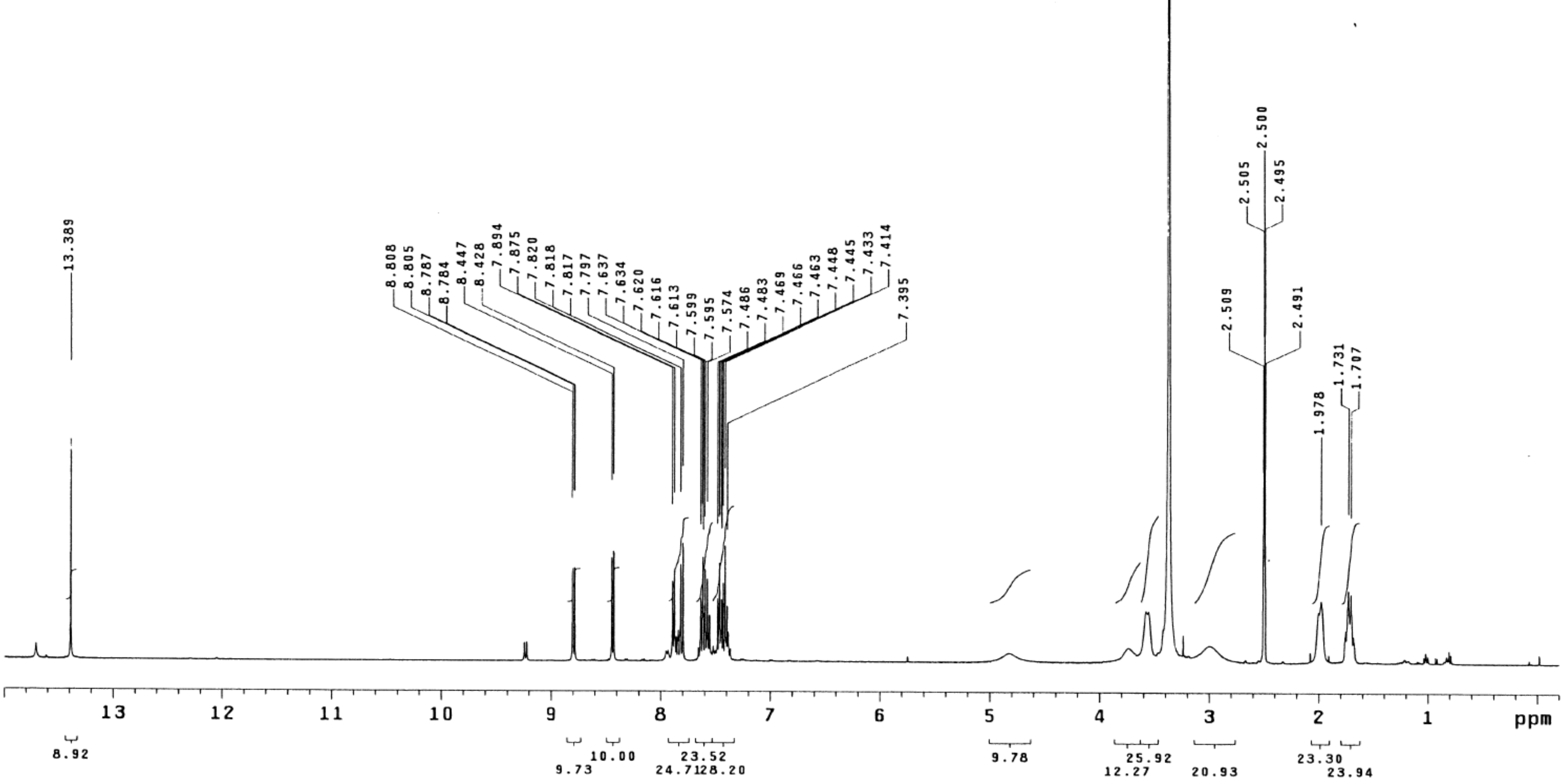

H,H-NOH

Pulse Sequence: s2pul
UNITYplus-400 "unity400"
Date: Oct 12 2011
Solvent: DMSO
Ambient temperature
Total 5328 repetitions

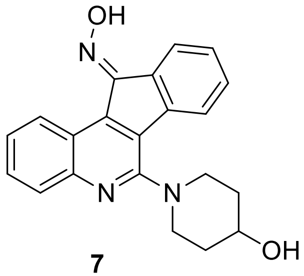

7

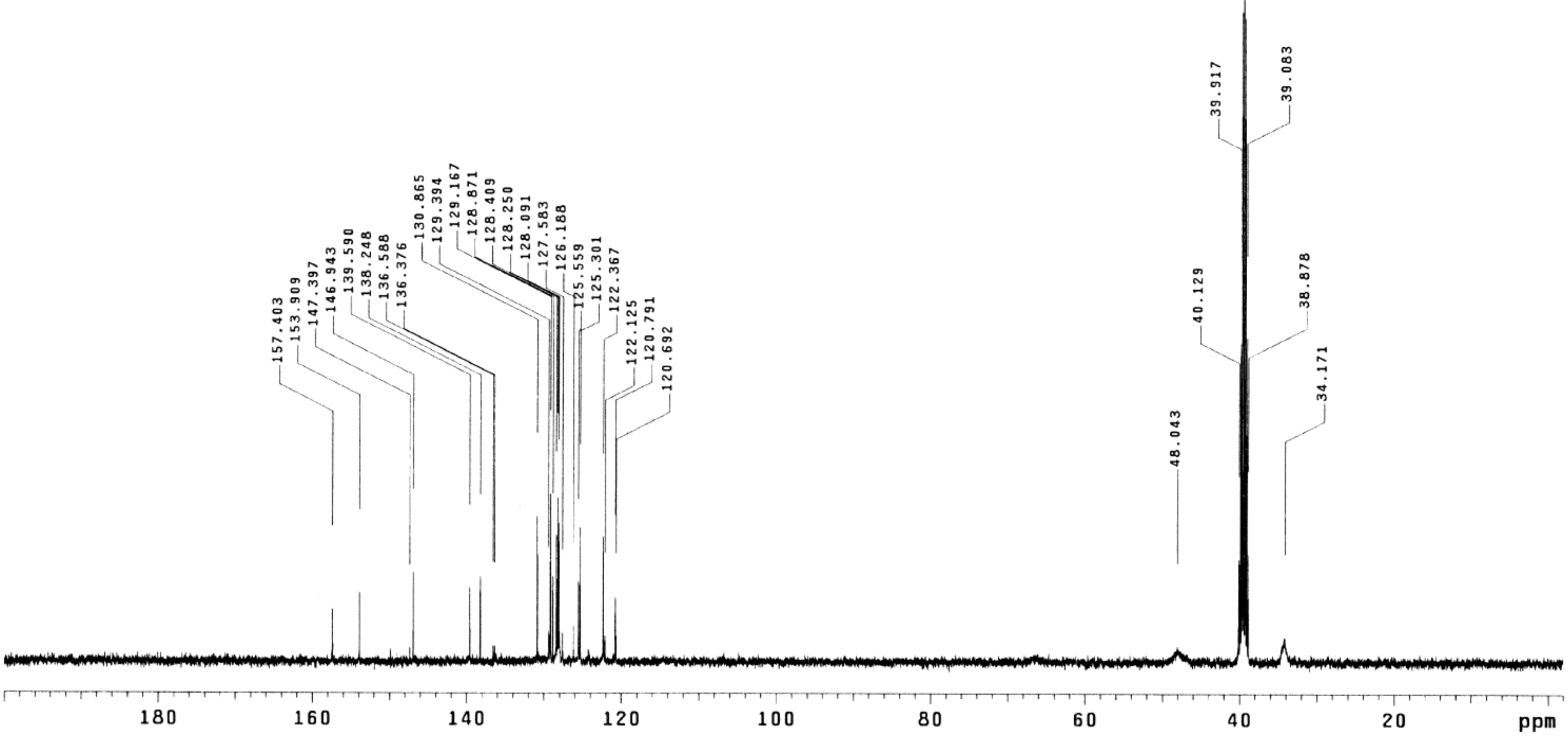

WCH-4376z

Pulse Sequence: s2pul
UNITYplus-400 "unity400"
Date: May 8 2012
Solvent: DMSO
Ambient temperature
Total 64 repetitions

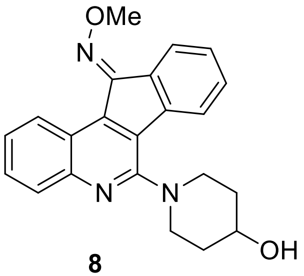

8

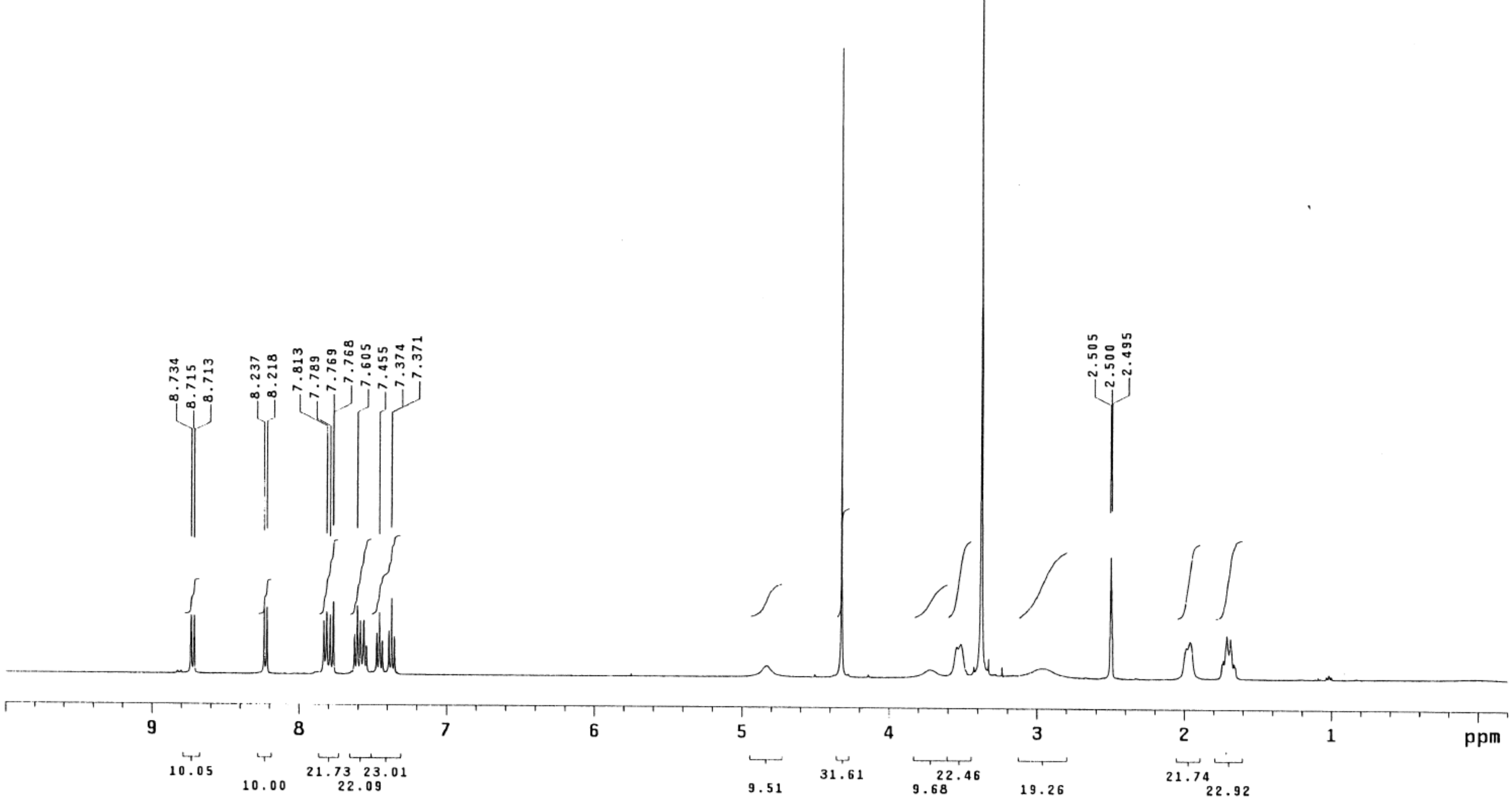

WCH-4376z

Pulse Sequence: s2pul
UNITYplus-400 "unity400"
Date: May 8 2012
Solvent: DMSO
Ambient temperature
Total 26720 repetitions

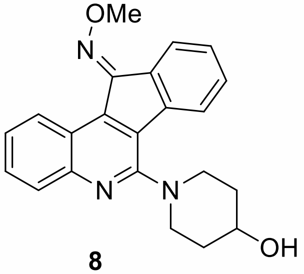

8

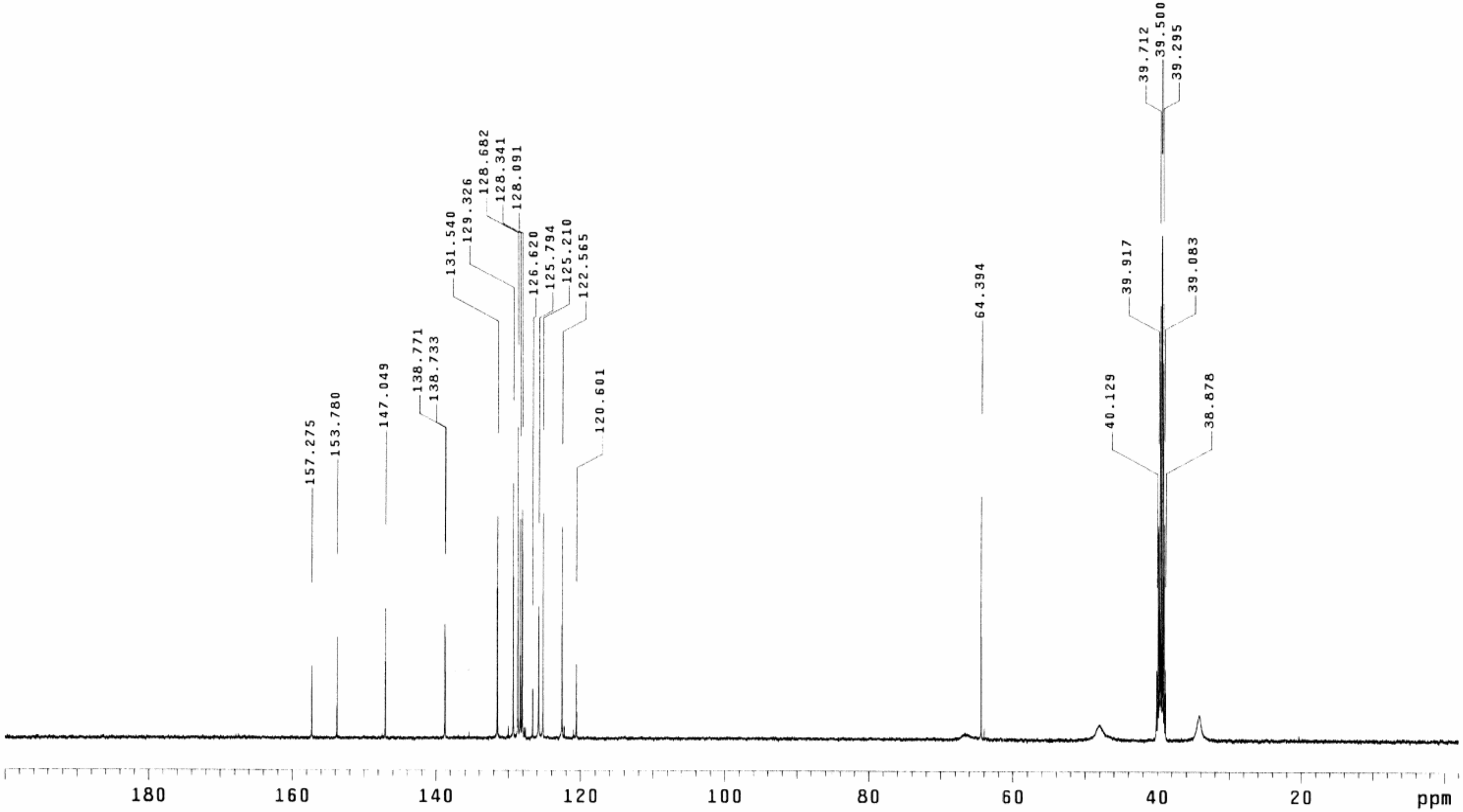

H,H-6-4OHpip-sem

Pulse Sequence: s2pul
UNITYplus-400 "unity400"
Date: Apr 30 2012
Solvent: DMSO
Ambient temperature
Total 64 repetitions

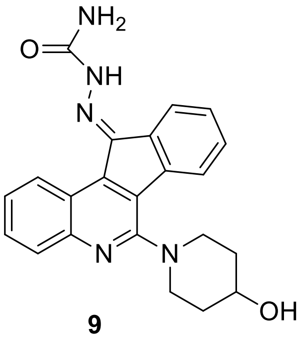

9

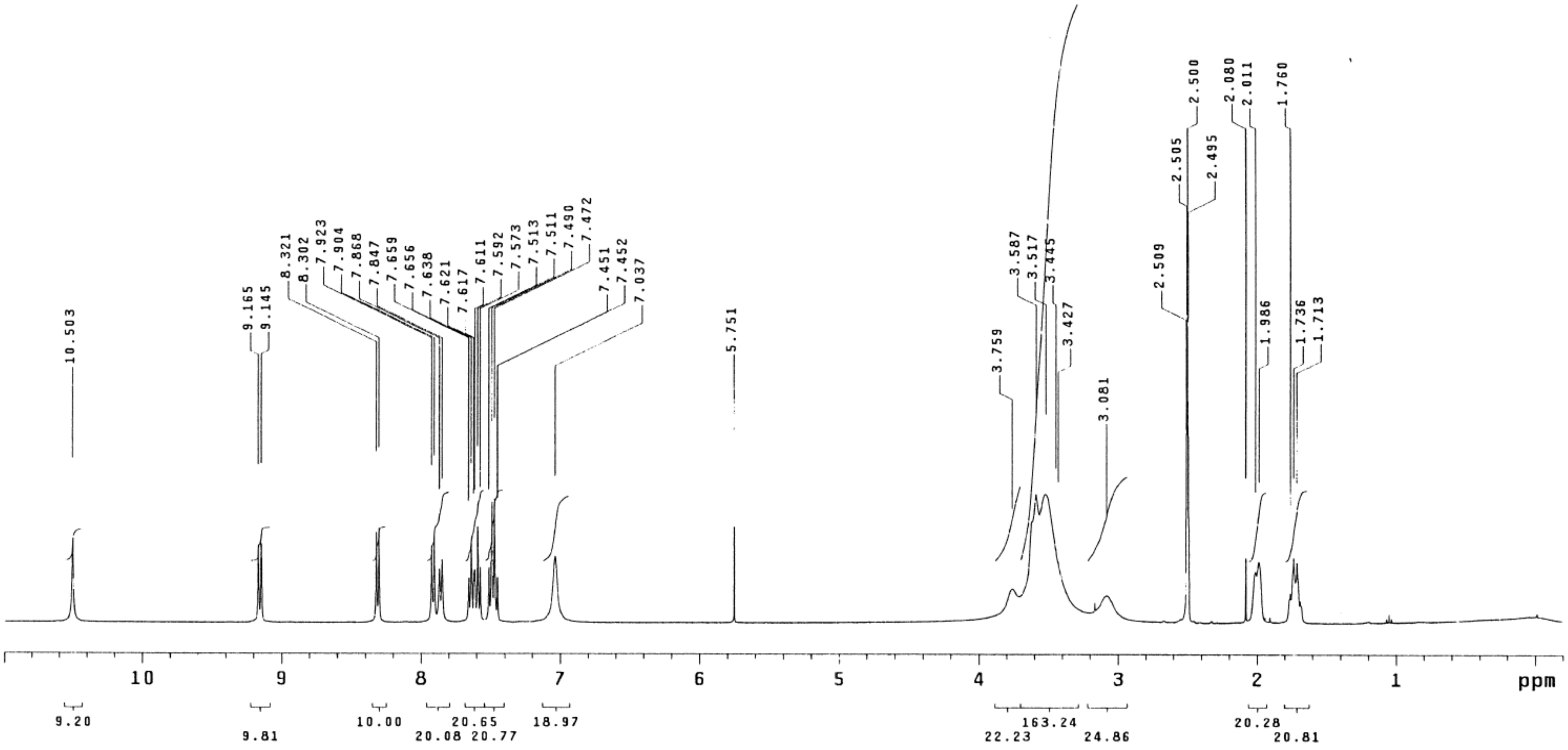

H,H-6-4OHpip-sem

Pulse Sequence: s2pul
UNITYplus-400 "unity400"
Date: Apr 30 2012
Solvent: DMSO
Ambient temperature
Total 14784 repetitions

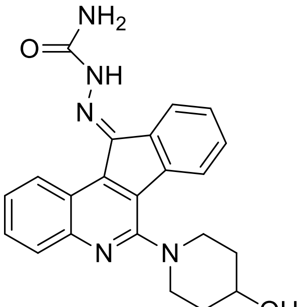

9

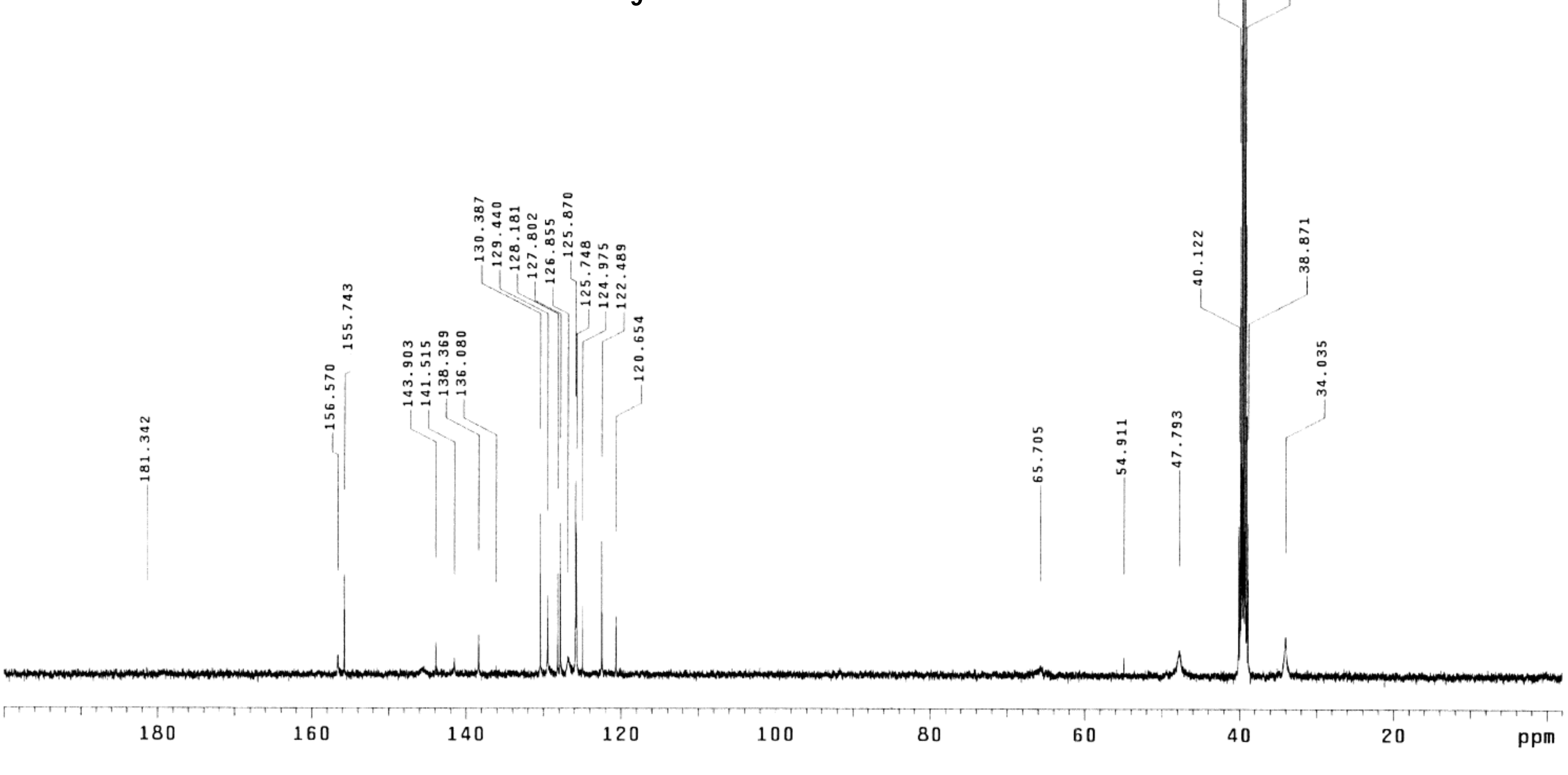

Thiosemicarbazide

Mercury-400BB "Mercuryplus400"
Date: Nov 1 2011
Solvent: DMSO
Ambient temperature
Total 48 repetitions

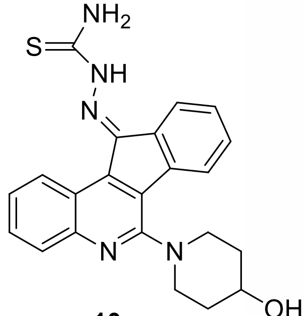

10

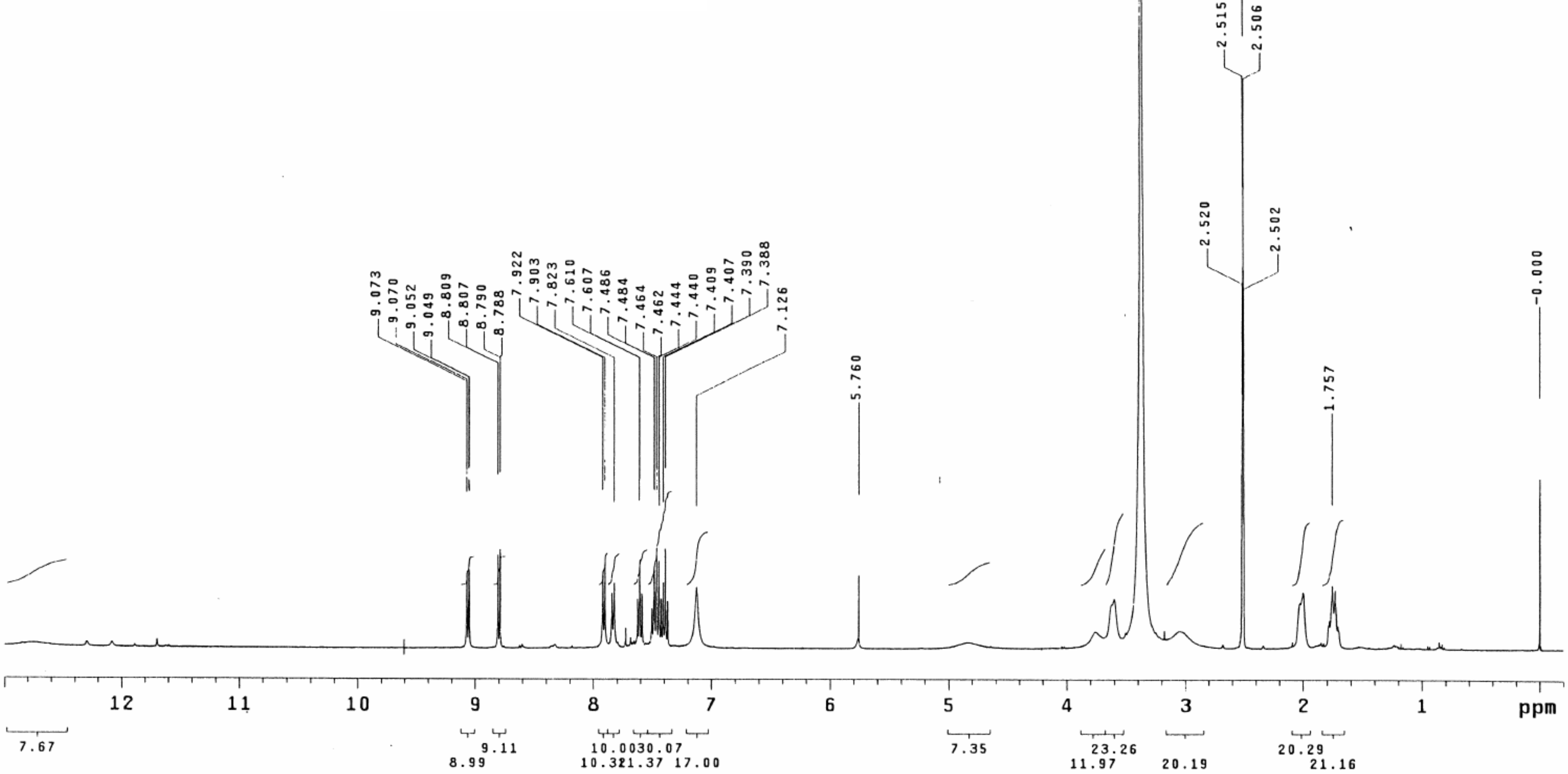

Thiosemicarbazide

Mercury-400BB "Mercuryplus400"
Date: Nov 1 2011
Solvent: DMSO
Ambient temperature
Total 13712 repetitions

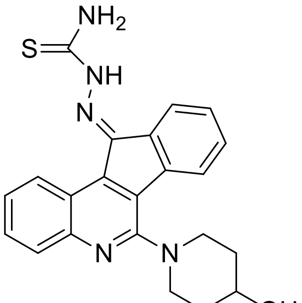

10

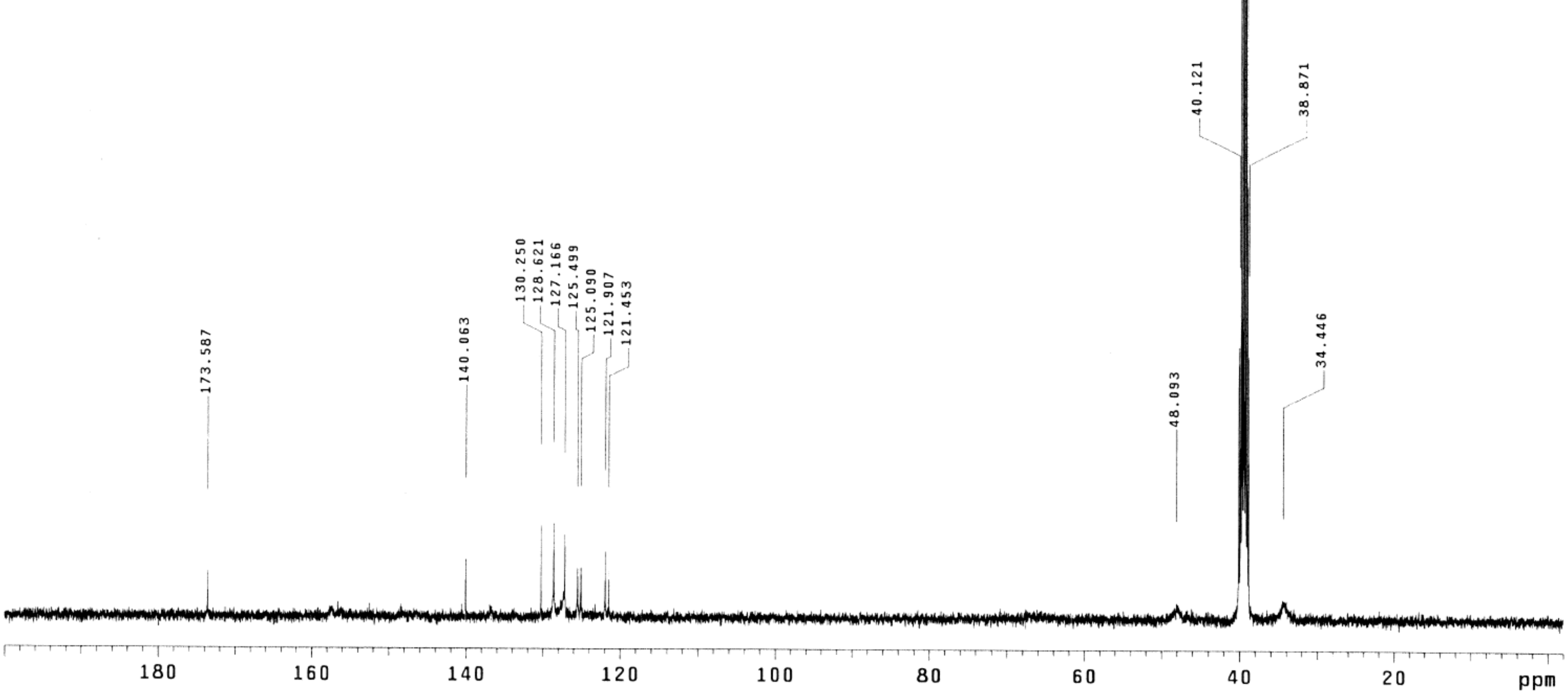

STANDARD 1H OBSERVE
H, H-4OH-piper-11-phenyl
Mercury-400BB "Mercuryplus400'
Date: Oct 12 2011
Solvent: DMSO
Ambient temperature
Total 80 repetitions

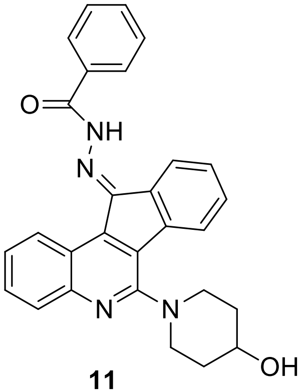

11

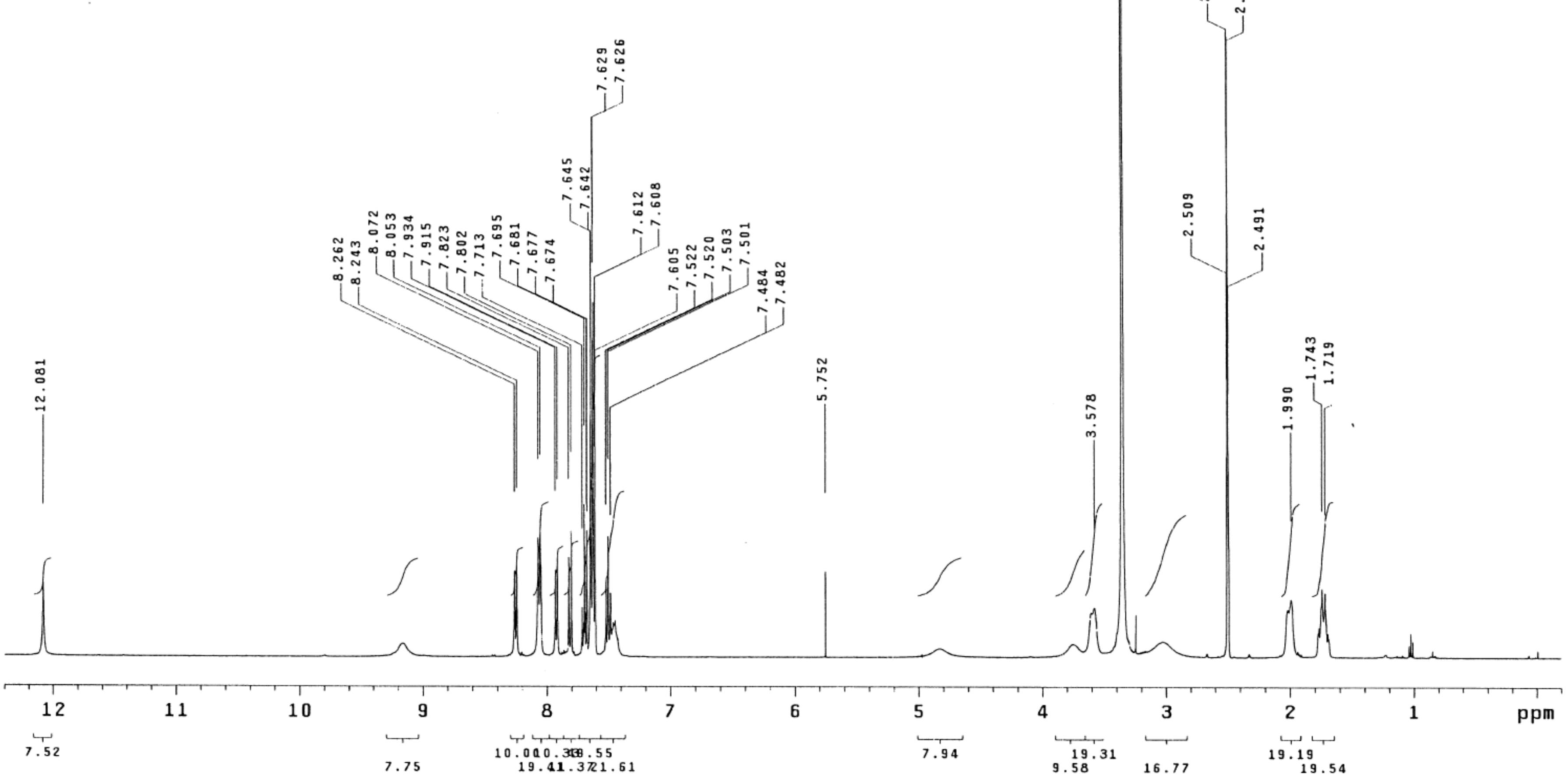

H,H-4OH-piper-11-phenyl

Mercury-400BB "Mercuryplus400"
Date: Oct 12 2011
Solvent: DMSO
Ambient temperature
Total 6512 repetitions

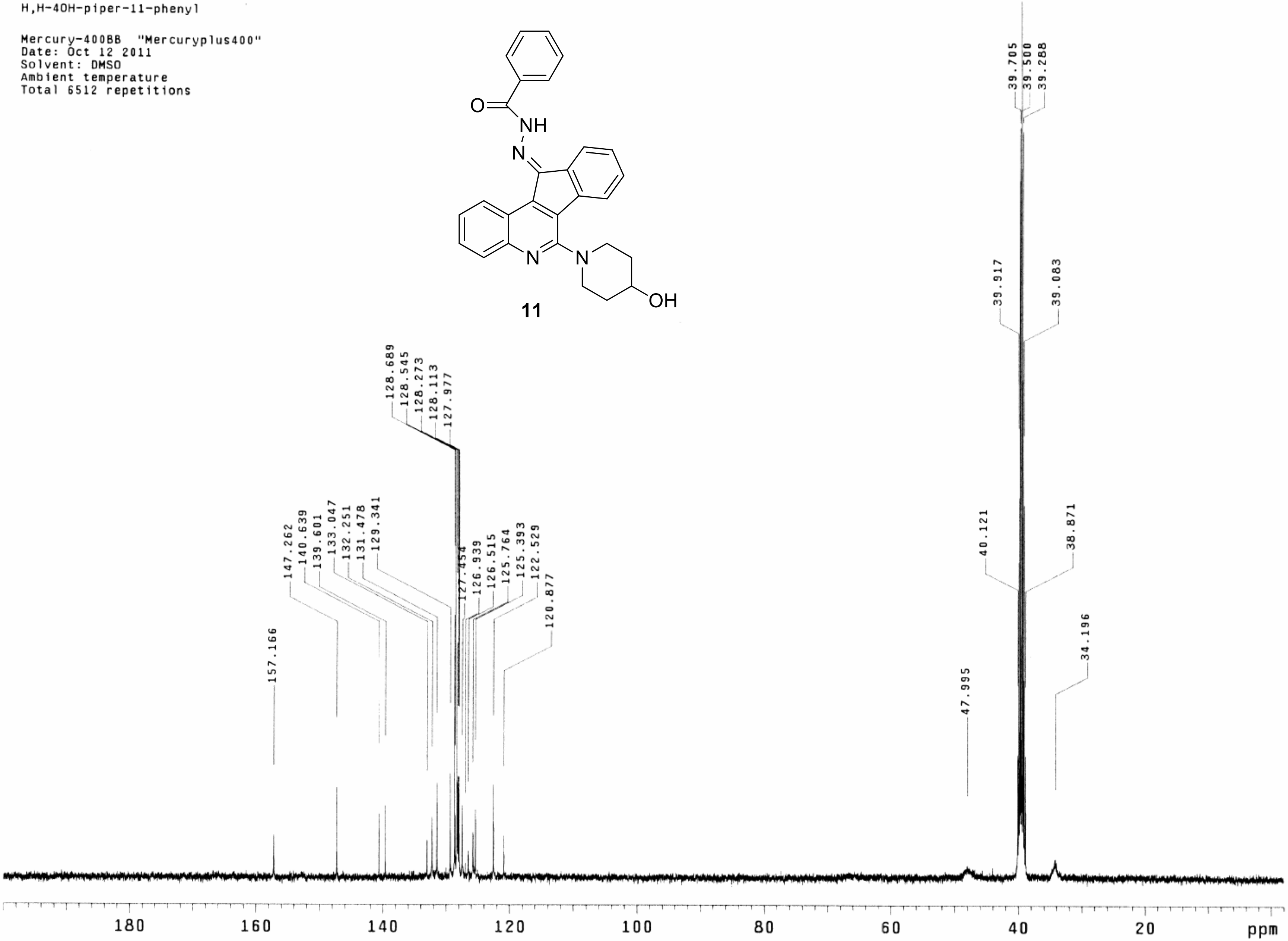

11

H,H-4OH-piper-11-iso

Mercury-400BB "Mercuryplus400"
Date: Oct 12 2011
Solvent: DMSO
Ambient temperature
Total 80 repetitions

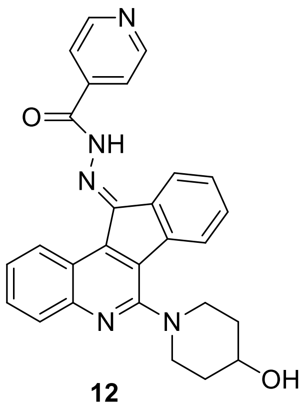

12

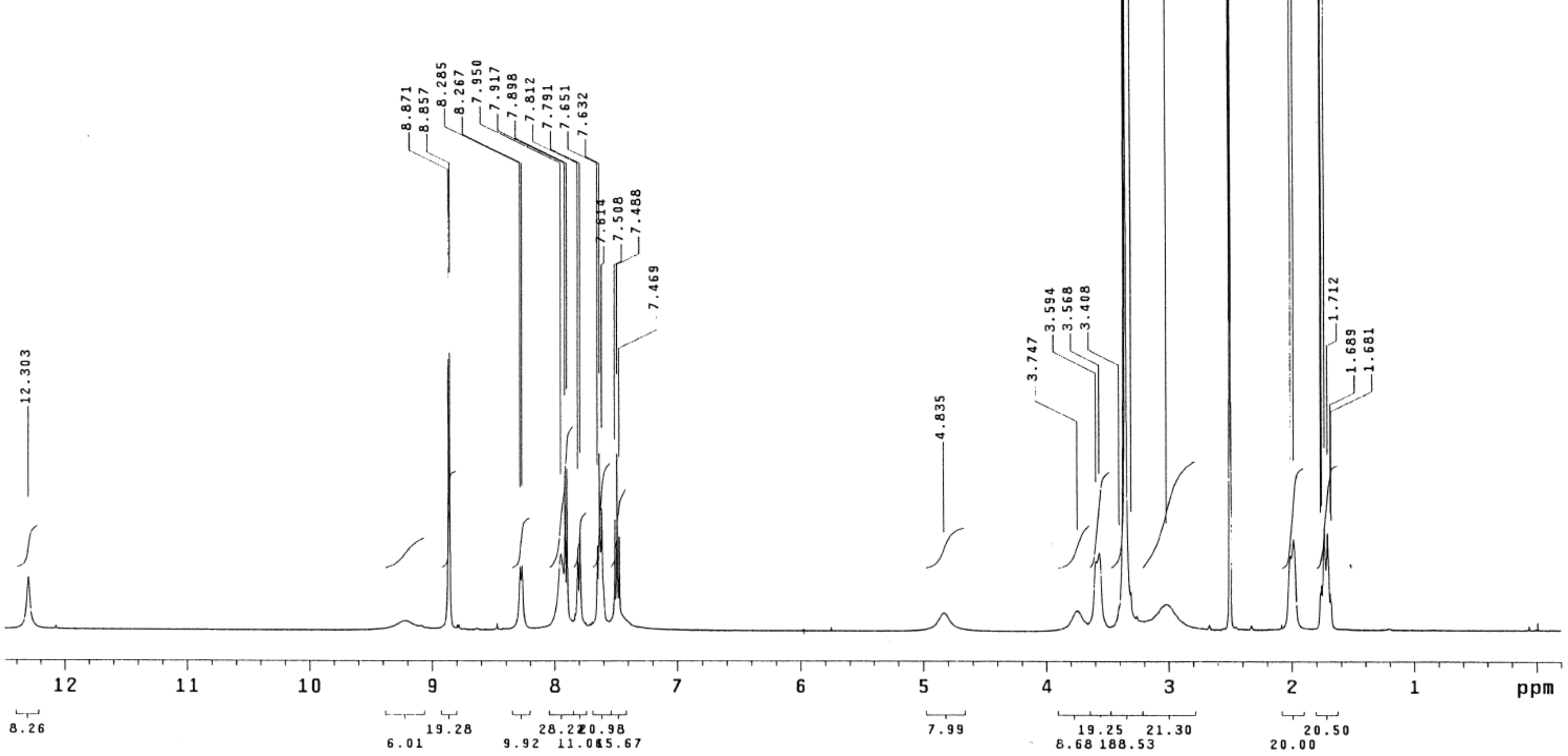

H,H-4OH-piper-11-iso

Mercury-400BB "Mercuryplus400"
Date: Oct 12 2011
Solvent: DMSO
Ambient temperature
Total 9536 repetitions

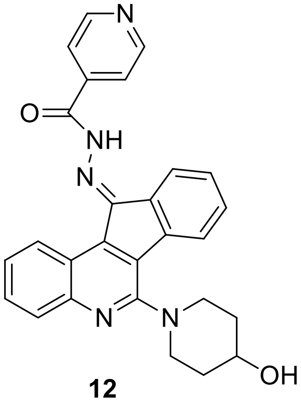

12

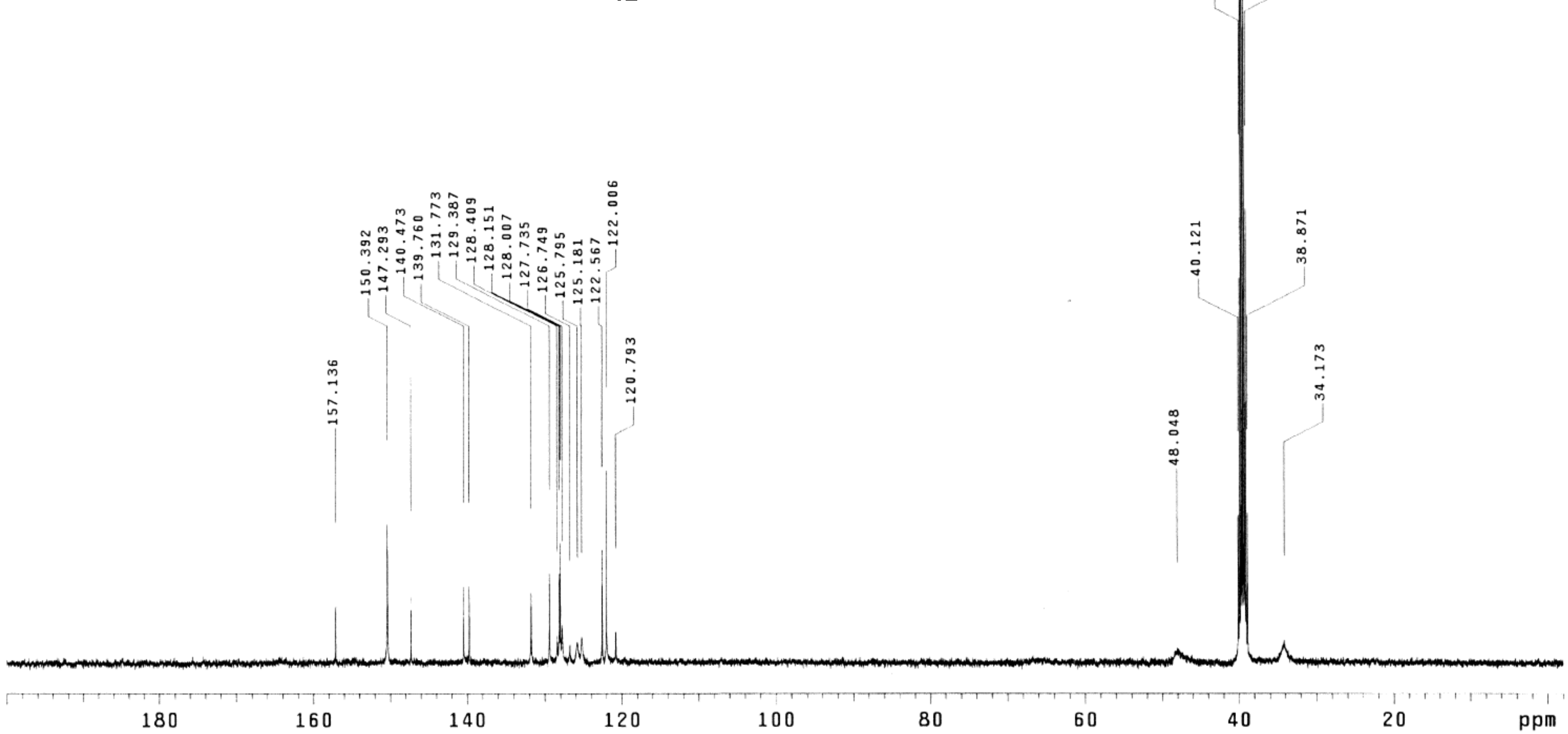

Supplement: Supplementary File 1 [file molecules-22-01001-s001.pdf]
